# An Organ‐on‐Chip Platform for Strain‐Controlled, Tissue‐Specific Compression of Cartilage and Mineralized Osteochondral Interface to Study Mechanical Overloading in Osteoarthritis

**DOI:** 10.1002/adhm.202501588

**Published:** 2025-06-25

**Authors:** Andrea Mainardi, Anastasiya Börsch, Paola Occhetta, Robert Ivanek, Martin Ehrbar, Lisa Krattiger, Philipp Oertle, Marko Loparic, Ivan Martin, Marco Rasponi, Andrea Barbero

**Affiliations:** ^1^ Department of Biomedicine University Hospital Basel University of Basel Hebelstrasse 20 Basel 4031 Switzerland; ^2^ Department of Electronics, Information and Bioengineering Politecnico di Milano Via Golgi 39 Milan 20133 Italy; ^3^ Department of Biomedical Engineering University of Basel Gewerbestrasse 14 Allschwil 4123 Switzerland; ^4^ Swiss Institute of Bioinformatics Elisabethenstrasse 43 Basel 4051 Switzerland; ^5^ Department of Obstetrics University Hospital Zurich Frauenklinikstrasse 10 Zurich 8091 Switzerland; ^6^ Zurich Centre for Integrative Human Physiology Winterthurerstrasse 190 Zürich 8057 Switzerland; ^7^ ARTIDIS AG Hochbergerstrasse 60C Basel 4057 Switzerland

**Keywords:** mechanotransduction, organ‐on‐chip, osteoarthritis, osteochondral unit, single cell RNA sequencing

## Abstract

Altered joint loading due to articular malalignment, instability, or trauma is an important risk factor for osteoarthritis (OA), the most prevalent musculoskeletal disease worldwide. However, the molecular links between aberrant mechanics and OA initiation/progression remain unclear due to the lack of human models capturing the interplay of joint tissues in a mechanically active environment. Replicating the strain gradient across the osteochondral interface remains an unmet challenge. Here, an OsteoChondral Unit (OCU)‐on‐Chip platform is engineered and functionally validated where composite hyaline cartilage‐mineralized subchondral microtissues are exposed to strain‐controlled, tissue‐specific compression levels akin, respectively, to those of cartilage and of the mineralized tissue at the osteochondral interface in vivo. Upon hyperphysiological loading of the OCU‐on‐Chip, an increase in the release and accumulation of calcium crystals, as reported in OA patients, is observed. Using single‐cell RNA sequencing, the role of the mineralized subchondral layer in sustaining chondrocyte subpopulations implicated in OA is demonstrated, and an overview of the transcriptional machinery activated by mechanical overstimulation is provided. The OCU‐on‐Chip captures clinically observed changes including alterations in ribosome biogenesis and apoptosis‐related pathways. Thus, it represents a valuable model for investigating mechanisms upstream of cartilage degeneration and may facilitate the identification of novel druggable biological pathways.

## Graphical abstract

A mechanically active OsteoChondral Unit (OCU)‐on‐Chip platform mimicking the OCU's functional anatomy and the strain gradient across the osteochondral interface is presented. Upon compartment‐specific hyperphysiological compression, the model replicates mechanisms observed in osteoarthritis (OA) progression, such as calcium crystal accumulation, chondrocyte subpopulation dynamics, and transcriptional responses to mechanical overstimulation, thereby providing a robust system for investigating OA pathophysiology and identify therapeutic targets.

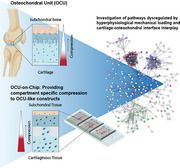

## 1 Introduction

Osteoarthritis (OA) is a highly prevalent, whole‐joint, and multi‐etiological disease with no available pharmacological cure.^[^ ^1^, ^2^ ^]^ OA's pathological alterations include cartilage erosion, synovial inflammation,^[^ ^3^ ^]^ and subchondral bone modifications.^[^ ^4^, ^5^ ^]^ However, the cause‐effect relationships of these processes are still not understood, and the precise origin of the disorder remains unknown. Together, the crosstalk among different articular compartments and joints’ mechanically active environment introduce a multiplicity of confounding factors. Thus, innovative preclinical OA models balancing complexity and strict experimental control could be invaluable in pinpointing the contributors to OA's pathogenesis.

In this framework, Organs‐on‐Chip (OoCs) are gaining consensus as tools for modeling OA and screening anti‐OA drugs by leveraging the intrinsic advantages of the microscale (e.g. diffusion‐driven transport phenomena and faster reaction/differentiation kinetics),^[^ ^6^, ^7^, ^8^, ^9^ ^]^ and the architectural flexibility of microfabrication. This enables the mimicking of tissues’ microstructure and organs’ functionalities with otherwise unachievable fidelity.^[^ ^10^ ^]^

Pioneering OoC systems for OA studies focused on modeling cartilage.^[^ ^11^, ^12^, ^13^, ^14^ ^]^ Nonetheless, the whole‐joint nature of OA makes it necessary to study tissue interplay to understand its driving mechanisms. For this purpose, different multi‐tissues OoCs have been introduced, including osteochondral‐synovial‐adipose miniature‐tissues analogs,^[^ ^15^ ^]^ systems enabling chondrocytes – synovial cells crosstalk^[^ ^16^ ^]^ or capturing monocytes extravasation,^[^ ^17^ ^]^ as well as osteochondral models with various degrees of cellular complexity.^[^ ^18^, ^19^, ^20^, ^21^ ^]^ Yet, none of these joint‐on‐chip models incorporate mechanical stimuli, a fundamental factor of joint pathophysiology.

OA is recognized as a multifactorial pathology with molecular, genetic, and environmental influences, and it has been defined as a heterogeneous collection of diseases with overlapping clinical phenotypes and different molecular endotypes.^[^ ^22^ ^]^ One of the most recognized and diffused phenotypes is the so‐called ‘mechanical phenotype’ – induced by trauma or joint malalignment/destabilization.^[^ ^23^, ^24^ ^]^ While various chondrocytes’ mechano‐sensing mechanisms have been discovered (e.g. integrins, the primary cilium, and ion channels such as TRPV4^[^ ^25^ ^]^ and PIEZO1/2^[^ ^26^ ^]^), the molecular links between aberrant mechanics and OA progression remain unclear. This grants the need for investigations that inherently necessitate mechanically active systems.

We previously introduced a Cartilage‐on‐Chip (CoC) model, demonstrating that applying a 30% hyperphysiological confined compression (HPC) to human articular chondrocytes (hACs) leads to recapitulation of OA traits encompassing inflammation, a shift in cartilage homeostasis toward catabolism, and the onset of a chondrocyte hypertrophic phenotype.^[^ ^14^ ^]^ The model was also validated as a drug screening platform.^[^ ^7^, ^14^, ^27^ ^]^ Similarly, others introduced OoC platforms to provide chondrocytes with different compression levels^[^ ^12^ ^]^ or with multimodal^[^ ^13^ ^]^ and multi‐directional^[^ ^28^ ^]^ mechanical stimuli.

**Figure 1:**
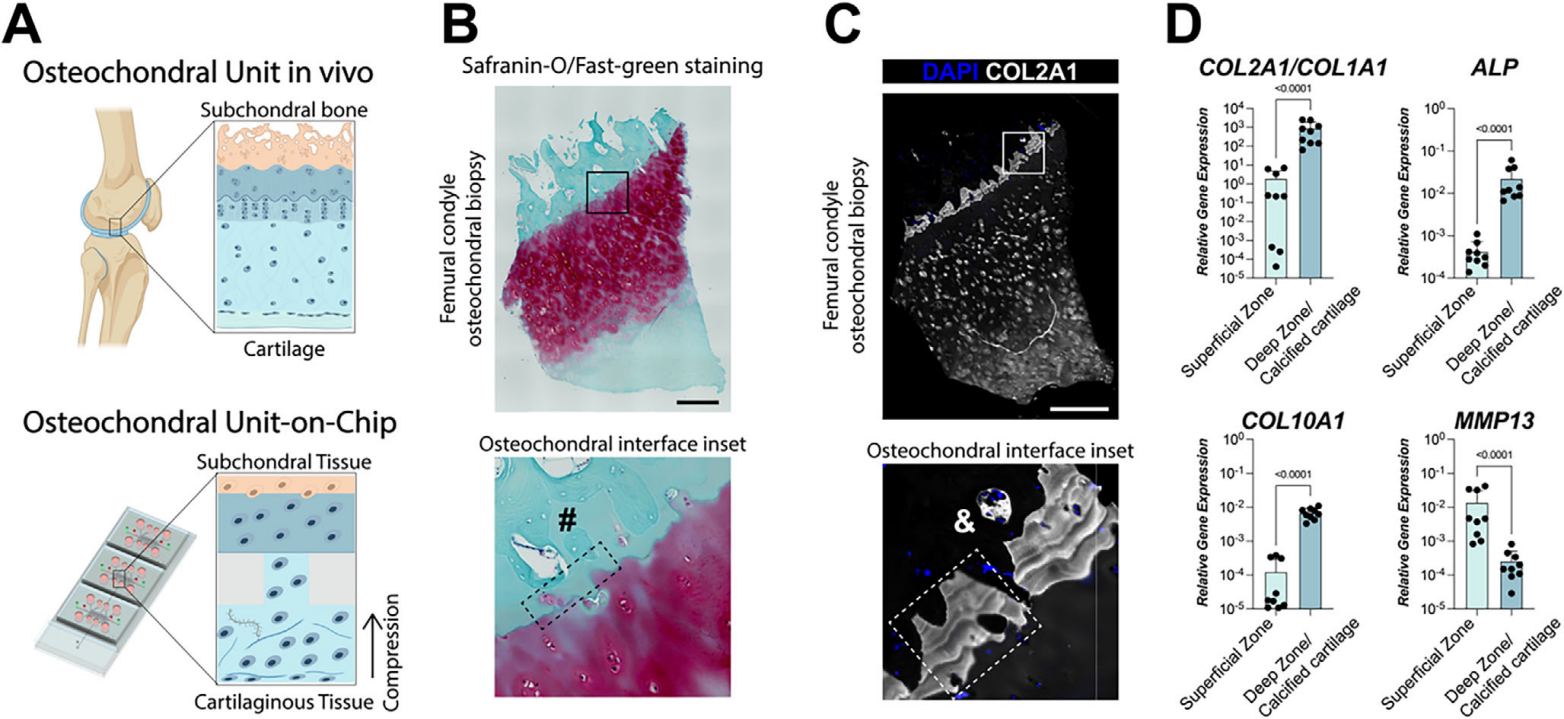
OCU‐on‐Chip concept and the osteochondral interface in human knees. A) Schematization of the knee OCU in vivo and of the OCU‐on‐Chip. B) Safranin‐O/fast‐green staining of an osteochondral biopsy from the femoral condyle of an OA patient undergoing knee replacement. The rectangle highlights the inset area, where the hashtag (#) marks an osteon‐like structure in the calcified cartilage layer, and the hashed rectangle highlights the osteochondral interface. Scale bar 500 µm. C) Immunofluorescence image of an osteochondral biopsy. The rectangle indicates the inset area, where the sign (&) indicates a cartilaginous area within the subchondral bone plate, and the hashed rectangle points to the osteochondral interface area. Scale bar 500 µm. D) RT‐qPCR quantification of the gene expression in different zones of osteochondral samples. Cartilage samples were harvested from the femoral condyles of patients undergoing knee replacement for OA, from areas without signs of cartilage degradation or surface fibrillation. Cartilage from the superficial zone and from the deep zone/calcified cartilage were collected and analyzed separately (*n* = 3 patients, for each patient *n* = 3 separate areas were harvested). Statistical significance was determined by paired *t*‐test (normal populations) and Wilcoxon test (non‐gaussian populations). (Adjusted) *p*‐values < 0.05 are reported on the graph. Expression levels of all genes were normalized to GAPDH expression. Values are reported as mean + s.d. Populations’ normality was assumed if both Shapiro‐Wilk and Kolmogorov‐Smirnov tests resulted positive.

Nevertheless, load transfer in diarthrodial joints is not mediated by cartilage only, but rather by the osteochondral unit (OCU), a bio‐composite constituted by hyaline cartilage, calcified cartilage, and subchondral cortical and trabecular bone^[^ ^1^ ^]^ (**Figure** **1**A).

During OA's pathogenesis, the composition, functional properties, and structure of the whole OCU are altered due to cartilage‐bone crosstalk and impaired OCU mechanics,^[^ ^2^, ^5^, ^29^ ^]^ though the timing and causal relationships of these events remain controversial. Different studies investigated the inter‐relation between cartilage and bone,^[^ ^18^, ^21^, ^30^ ^]^ while very limited attention was granted to the mineralized tissues at the osteochondral interface,^[^ ^31^ ^]^ even if these layers (e.g. calcified cartilage) have a predominant role in transmitting forces and providing a direct interface between cartilage and bone.^[^ ^4^ ^]^ Moreover, the osteochondral interface is highly affected by OA with modification including thickening and hypermineralization,^[^ ^32^ ^]^ and the re‐activation of developmental programs leading to chondrocytes hypertrophy and endochondral ossification processes (which affect the whole OCU).^[^ ^1^, ^4^ ^]^ In OA joints, structures similar to osteons can be detected in the calcified cartilage layer (Figure 1B), and cartilaginous collagen type II (COL2A1)‐rich zones in the subchondral bone plate (Figure 1C).

An OoC system that replicates the cartilage‐bone interface in a mechanically competent environment (as conceptualized in Figure 1A) is therefore highly needed to better understand these dynamic processes. Yet, its development is associated with specific engineering challenges. Cartilage, calcified cartilage, and bone have a macroscale Young's modulus that ranges from a few MPa to GPa and depends on the tissues’ complex multi‐scale hierarchical organization.^[^ ^33^, ^34^ ^]^ Consequently, strain levels vary by at least one order of magnitude across OCU tissues, and recapitulating this gradient with a device that is versatile and easy to use remains an unmet need.

Moreover, the cartilage surface and its deep zone/calcified cartilage have expression levels of matrix‐associated genes and of markers of Wnt and BMP activation,^[^ ^35^ ^]^ hypertrophy, fibrosis, ossification processes, and degradation/inflammation that vary up to 2–3 orders of magnitude, as we verified analyzing human knee joints (Figure 1D; Figure S1, Supporting Information). These differences need to be considered in assessing the interplay of hyaline cartilage and subchondral mineralized tissues.

Here, we propose an OCU‐on‐Chip providing tissue‐specific mechanical stimuli and demonstrate how the model can be used to investigate overload‐induced biological processes related to interactions at the osteochondral interface.

First, we introduce and functionally validate a novel microfluidic structure, the Vertical capillary Burst Valve (VBV), through which bi‐phasic micro constructs replicating the OCU functional anatomy can be easily obtained and subjected to strain‐controlled, compartment‐specific compression levels.

Subsequently, we achieve OCU‐like 3D human cellular constructs with gene expression levels and ECM composition comparable to human joint tissue counterparts – whereby with OCU we intend hyaline cartilage and the calcified cartilage layer at the osteochondral interface (insets in Figure 1B,C). We show that by applying hyperphysiological compression to the cartilage layer while simultaneously applying a physiological, almost null, compression to the mineralized layer, the OCU‐on‐Chip recapitulates hypermineralization, chondrocalcinosis, and the release of calcium crystals observed in OA patients.^[^ ^36^ ^]^

Finally, through scRNA‐seq, we demonstrate i) the critical role of the mineralized layer in the development of chondrocyte subpopulations implicated in OA pathogenesis and ii) the further regulation of the system upon exposure to mechanical loading, ultimately mirroring fundamental transcriptional changes recently described in OA (e.g., alterations in the expression of genes encoding for ribosomal proteins^[^ ^37^ ^]^).

We thus propose the OCU‐on‐Chip as a tool for in vitro modeling of various aspects of the mechanical phenotype of human OA, which might aid the identification of molecular targets for disease‐modifying therapies.

## 2 Results and Discussion

### 2.1 Devising an OoC Device for Compression of Bi‐Layered 3D Microtissues

We conceived a microscale device that can host two directly interfaced, superimposed 3D cellular constructs, and subject them to tissue‐specific mechanical compression.

**Figure 2:**
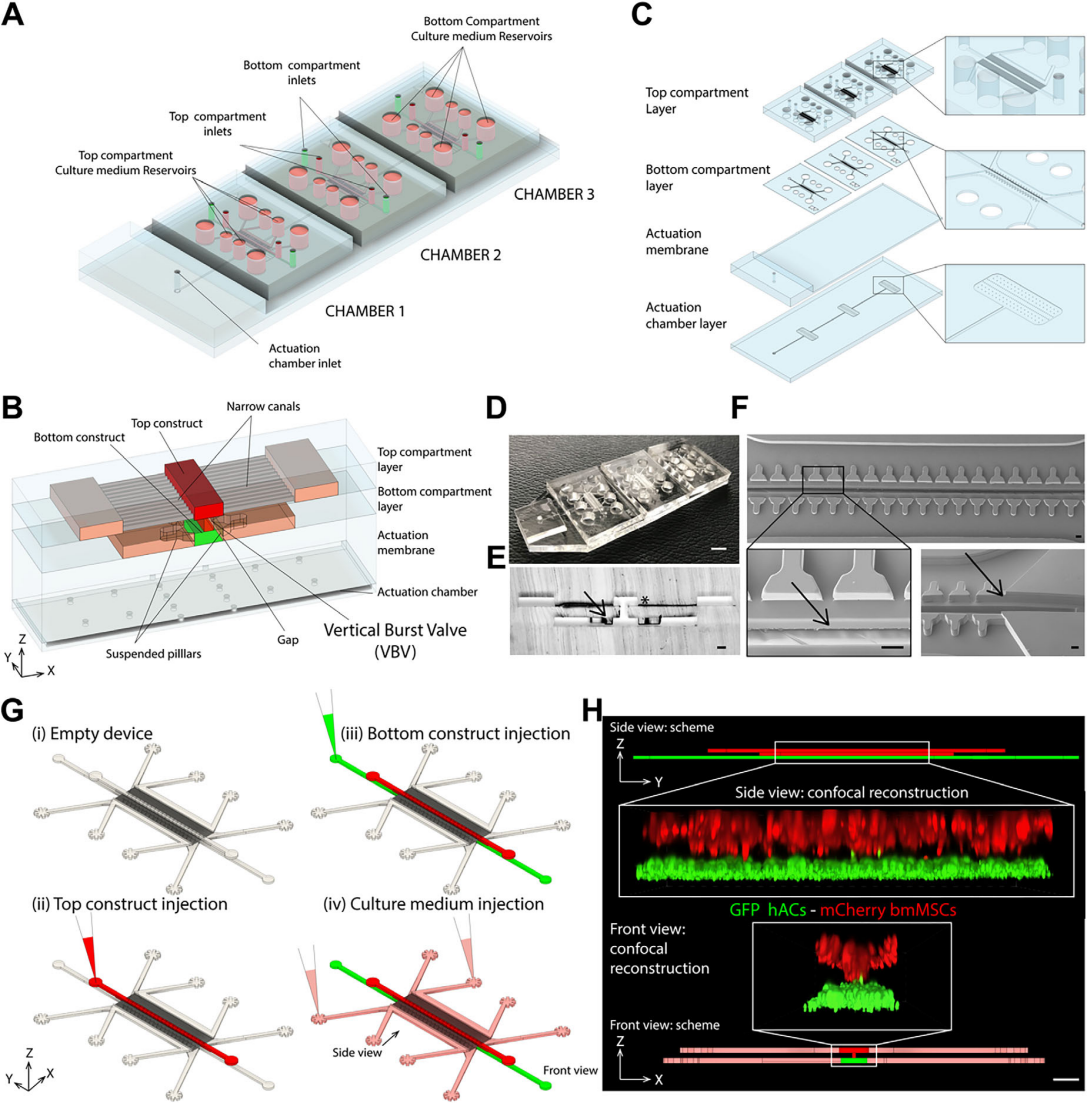
Microfluidic device to provide bi‐layer 3D constructs with tissue‐specific mechanical compression: geometry and production. A) Device 3D schematization. B) Schematic detailing the inner structure of the device. C) Sketch of the 4 PDMS layers composing the device. A Thick PDMS slab (height 3 mm) is added to the actuation membrane to increase retention of the tube providing pressure to the actuation compartment. D) Photograph of a device. Scale bar, 4 mm. E) Photograph of a device section, the arrow points to the suspended pillars, and the asterisk indicates the VBV geometry. Scale bar, 100 µm. F) Scanning Electron Microscopy (SEM) images of the bottom compartment observed from below. Arrows point to the necking connecting bottom and top compartment (left) and to the gap below the T‐shaped pillars (right). Scale bar, 100 µm. G) Schematic of the injection procedure. H) Confocal microscopy reconstruction of cell‐laden constructs seeded in the two culture compartments. mCherry expressing bone marrow derived mesenchymal stromal cells (bmMSCs) and green fluorescent protein (GFP) expressing hACs were embedded in an enzymatically cross‐linkable and degradable 2% w/v polyethyleneglycole (PEG)‐based hydrogel^[^ ^44^ ^]^ and seeded, respectively, in top and bottom compartments. Images and 3D schematics (at the top and at the bottom) in the YZ and XZ planes highlight the direct contact interface between the two constructs across one‐third of their width (along the X‐axis) and along the entire tissue length (along the Y‐axis). Scale bar, 100 µm. The VBV necking area constitutes the only contact point between the top and bottom compartment, as can be observed in the XZ plane.

The device comprises three independent functional units connected to a single pneumatic actuation chamber (**Figure** **2**A; Figure S2A,B, Supporting Information). Each functional unit is constituted by a bottom compartment for the cartilaginous tissue and a top compartment for the subchondral tissue (Figure 2B).

The bottom compartment is based on our previously introduced CoC platform:^[^ ^14^ ^]^ a central channel that hosts a 3D cell‐laden hydrogel is divided by two rows of overhanging pillars from lateral culture medium channels (Figure 1B). A flexible membrane separates it from an underlying actuation chamber. Upon pressurization of the actuation chamber, the membrane bends upward until it abuts against the pillars. The bottom compartment was dimensioned to obtain a precise 30% hyperphysiological compression (HPC), which we previously demonstrated to be sufficient to elicit an OA‐like phenotype in 3D cartilaginous micro constructs.^[^ ^14^ ^]^

The top compartment is constituted by a single channel hosting the subchondral tissue, connected to culture medium channels and reservoirs by two arrays of narrow canals (Figure 2B; Figure S2C, Supporting Information). The geometry of the canals was designed to delay the diffusion of solutes from the bottom compartment to the top compartment's reservoirs.

The top and bottom compartments are linked through a structure with a cross‐section shaped like a tilted “H” (Figure 2B) that we named “Vertical capillary Burst Valve” (VBV). By VBV we indicate a geometric striction in the section of a microfluidic channel that extends for the whole channel's length (width_striction_: width_channel_ = 1:3). If appropriately dimensioned, the striction allows to selectively fill either the bottom or top half of the channel and thus to obtain a biphasic construct constituted by two superimposed cell‐laden hydrogels. Details of the device and the dimensions of the VBV structure are provided in Figure S2D,E (Supporting Information).

The concept of the VBV was inspired by classic capillary burst valves (CBV), which are designed to stop an advancing front from going further on, left or right, and whose principle has been described before.^[^ ^38^, ^39^ ^]^ Leveraging the limited role played by gravitational forces at the microscale, we devised a geometry able to stop an advancing fluid from moving upward or downward. A schematic illustrating the CBV and VBV working principle is reported in Figure S3A,B (Supporting Information). Additionally, the VBV creates a discontinuity in the strain field of the two constructs. While the compression of the cartilaginous construct is determined by the dimension of the gap underneath the pillars in the bottom compartment (which regulates the actuation membrane's stroke), the deformation of the top subchondral construct is limited by the geometrical constraint introduced by the VBV. Such a strain field reflects the OCU deformation during gait, in which a high compressive strain on the cartilage surface corresponds to minimal deformations of underlying subchondral tissues.^[^ ^40^ ^]^

The device was realized in polydimethylsiloxane (PDMS), through a multi‐layer assembly procedure (Figure 2C). The sequence of steps necessary to obtain the VBV structure is detailed in Figure S4A–C (Supporting Information); a complete description of the fabrication procedure is reported in Supplementary Methods. The dimensions of the top compartment were optimized to maximize the ease and precision of fabrication (Supplementary Note S1, Figure S5A,B, Supporting Information). Due to the multi‐layer fabrication procedure, the top compartment can be tailored according to the specific experimental needs. Figure 2D–F represents details of a physical device, demonstrating a high adherence to the nominal geometry.

To functionally validate the platform, first, we showed that a bi‐layer tissue can be obtained with high reproducibility by simply injecting two cell‐laden hydrogels in the top and bottom compartments (Figure 2G,H). More than 80% of devices (N > 200 devices) could successfully be injected without leakage in neighboring channels. This success rate corresponds to the one of injecting two of our single‐compartment CoCs, demonstrating the robustness of the VBV. Second, the pressure necessary to achieve contact between the actuation membrane and pillars (i.e. 0.4 Atm) was experimentally determined as previously described^[^ ^14^ ^]^ (Supplementary Note S2, Figure S6A,B, Supporting Information). The thickness of the actuation membrane (800 µm) was optimized so that the actuation pressure depends exclusively on the bending stiffness of the membrane itself, preventing it from varying because of alterations in tissues’ mechanical properties during culture.

Finally, we assessed the diffusion kinetic of dextran (as a surrogate molecule for morphogens with a molecular weight in the range of 20 kDa, such as TGFβ^[^ ^41^ ^]^ and BMPs^[^ ^42^ ^]^) between the bottom and top compartments. To account for changes in tissue permeability due to cellular constructs remodeling over time, experiments were performed both immediately after injection of cells‐laden hydrogels in the device, and after 14 days of static maturation (Figure S7A, Supporting Information). Considering mature constructs, the top compartment design slowed‐down inter‐compartmental diffusion (dextran measured_top‐compartment‐reservoirs_/dextran provided: Day 0_24h_ = 0.43 ± 0.09, Day 14_24h_ = 0.21 ± 0.14, Figure S7B,C, Supporting Information), making the device suitable to discriminate if a solute is primarily secreted by the top or the bottom construct within a 12–24 h timeframe.

Overall, these data confirm the VBV's functionality, successful fabrication, and versatility in obtaining vertically stacked biphasic 3D constructs that can be mechanically stimulated through a pneumatic compartment. Previous strategies to achieve osteochondral micro constructs using hydrogels required coatings and sacrificial molds.^[^ ^21^, ^43^ ^]^ Our VBV approach limits the interface between compartments to 1/3 of their area but eliminates the necessity for laborious operations which impede  high experimental throughputs and the integration of pressure‐dependent components essential for mimicking the mechanically active joint environment.

### 2.2 Providing Compartment‐Specific Compression Levels: Device Mechanical Validation

Next, we tested if our OoC platform could actually provide constructs hosted in the top and bottom compartments with compression levels indicative of those experienced by OCU tissues in vivo (ranging from ‐35% in the superficial zone, to ‐1% in the deep zone/calcified cartilage layer,^[^ ^40^ ^]^ and to negligible levels at the subchondral interface).

We specifically aimed at introducing a strain‐controlled device in which compartment‐specific compression is dictated solely by the platform's geometry, independently from tissues’ mechanical properties. This ensures consistent mechanical stimulation throughout the culture period and makes the device suitable for various materials. Accordingly, in the validation phase, we assessed the strain fields of bi‐layer constructs with uniform mechanical properties, to confirm whether geometry alone could induce distinct compression levels.

**Figure 3:**
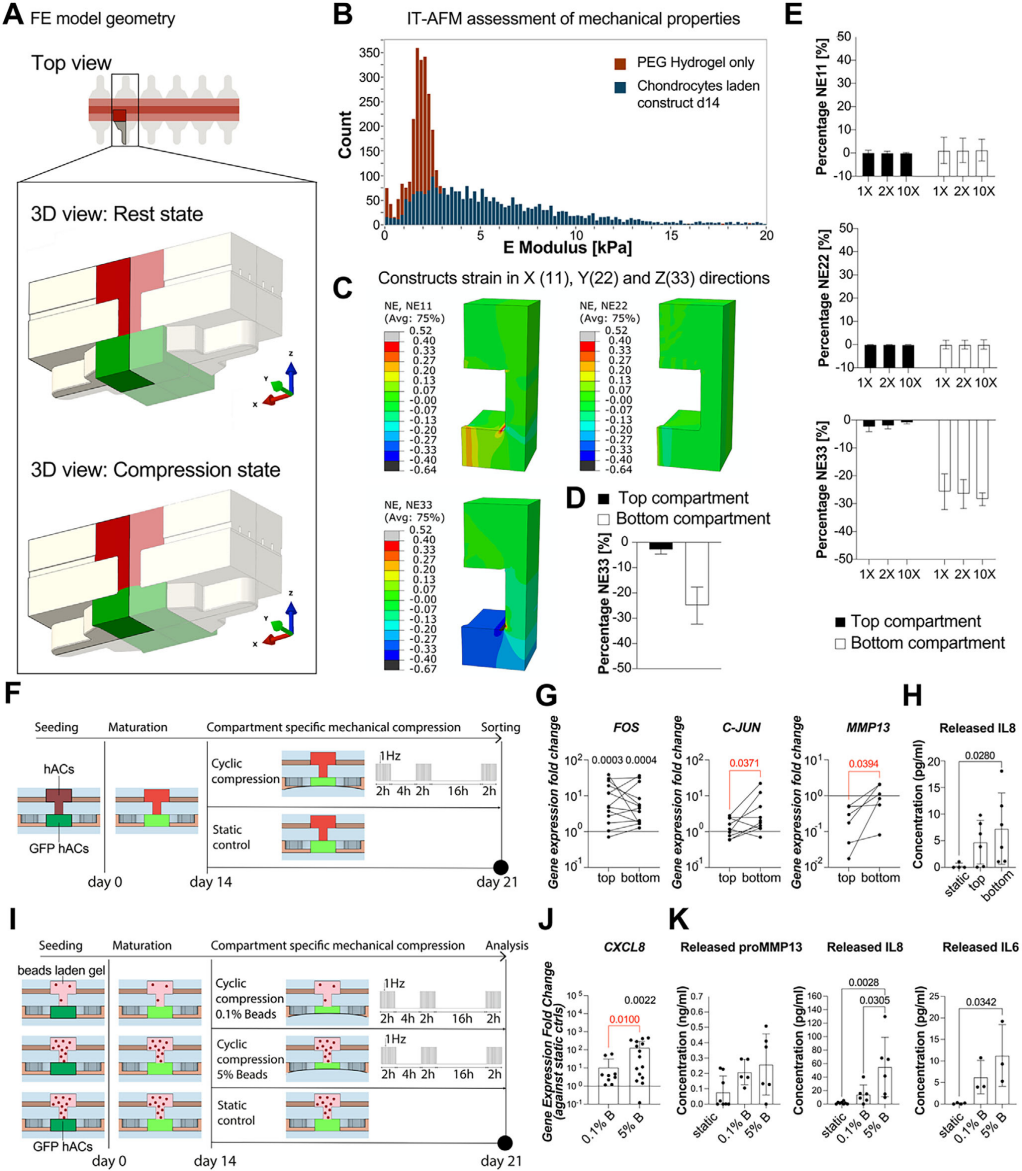
The OCU‐on‐Chip provides compartment‐specific compression levels: strain field estimation and biological validation. A) Schematization of the 3D model adopted for FE simulation. Top figure: top view of the constructs and of the T‐shaped pillars; the black rectangle encloses the minimal repetitive unit i.e. a volume that repeats itself periodically in the device. Bottom figure: 3D FE reconstructions of the minimal repetitive unit in rest state and upon compression. The shaded area represents the region of interest (ROI) i.e. the volume effectively adopted in simulations. Adopting a repetitive ROI volume allows to reduce the computational cost and increases the simulation accuracy by introducing a higher number of elements in a restricted region and accounting for the entire device through symmetry boundary conditions; B) IT‐AFM‐based assessment of the mechanical properties of the PEG hydrogel (*n* = 3 replicates) and of cartilaginous constructs cultured statically for 14 days in the device (*n* = 3 independently cultured samples from *n* = 1 donor). The histogram represents the number (i.e. the count) of indentation spots that resulted in having a Young's modulus of a given range. C) Contour plots of constructs’ strain field upon loading. The 3D volume represents the ROI volume of the cartilaginous construct upon compression, i.e. when the actuation membrane touches the bottom surface of the pillars. Both top and bottom compartments were considered filled with cartilaginous constructs (E = 3.66. kPa). NE11, NE22, and NE33 refer, respectively, to strains aligned with the X, Y, and Z axes. Strains are representative of the difference in dimension between the rest state and the compressed state divided by the dimension in the rest state. NE33, for instance, indicates the compression level in the Z direction D) Quantifications (mean ± s.d.) of compression levels in top and bottom constructs referred to simulations reported in C. E) Quantifications (mean ± s.d.) of top and bottom constructs nominal strains upon loading when varying the constructs’ mechanical properties. 1X, 2X, and 10X, refer to the modulus adopted for the hydrogel in the top compartment with respect to the one of the hydrogel in the bottom compartment (i.e. 1.9 kPa). The top construct was considered comprehensive of the volume in correspondence of the VBV necking. F) Experimental procedure adopted to verify that constructs in top and bottom compartments are subjected to different mechanical stimuli. G) RT‐qPCR quantification of the gene expression of hACs from top and bottom constructs upon loading (*n* ≥ 6 independently cultured samples from *n* ≥ 2 donors). When comparing cells from top and bottom constructs, statistical significance was determined by paired t‐test for normal populations and by Wilcoxon test for non‐gaussian populations ((adjusted) *p*‐values < 0.05 are indicated in red). When comparisons were performed with respect to static controls (indicated by the horizontal lines in black), statistical significance was determined by Kruskal‐Wallis test with Dunn's test for multiple comparisons ((adjusted) *p*‐values < 0.05 are indicated in black). For each donor, values were normalized for the expression of sorted static controls. Values are reported as mean + s.d. H) Quantification (mean ± s.d) of IL8 released in the culture medium (*n* = 6 independently cultured samples from *n* = 2 donors). Statistics by Kruskal‐Wallis test with Dunn's multiple comparison test. I) Experimental procedure used to verify if the OCU‐on‐Chip could be used to study the relationship between the mechanical properties of the subchondral layer and hACs’ response to loading. Polystyrene beads (either 0.1% or 5% in volume, 10 µm in diameter) loaded PEG hydrogels were seeded in the top compartment, hACs in the bottom compartment. J, K) An increase in the number of stiff inhomogeneities in the subchondral layer enhances chondrocytes response to pathological loading. J) Gene expression quantification (mean + s.d) through RT‐qPCR (*n* ≥ 9 independently cultured samples from *n* = 3 donors). Statistical significance was determined by Mann‐Whitney test to compare two populations (red), or by Kruskal‐Wallis test with Dunn's test for multiple comparisons with respect to static controls (indicated by horizontal black lines). Values were normalized for the expression of each donor's static controls. K) Quantification (mean ± s.d) of cytokines and degradative enzymes released in the culture medium. proMMP13 and IL8 concentrations were determined by ELISA (*n* = 6 independently cultured samples from n = 2 donors); IL6 concentration by Luminex analysis (*n* ≥ 3 independently cultured samples from n = 1 donor). Statistical significance was determined by one‐way ANOVA with Tukey's multiple comparison test for normal populations and by Kruskal‐Wallis test with Dunn's multiple comparison test for non‐normal distributions. For all graphs, populations’ normality was assumed if both Shapiro‐Wilk and Kolmogorov‐Smirnov tests resulted in positive. (Adjusted) *p*‐values < 0.05 are reported on the graph. GAPDH was used as a housekeeping gene for all RT‐qPCR data.

First, we estimated the constructs’ strain field through a 3D Finite Element (FE) numerical model (**Figure** **3**A; Supplementary Methods). A Biphasic Poroelastic (BPE) constitutive relation, which accurately predicts the non‐linear and deformation‐rate‐dependent strain of cartilage and hydrogels,^[^ ^45^, ^46^, ^47^ ^]^ was assumed for the constructs. Preliminary simulations to optimize the 3D model's geometry and mesh were performed considering both top and bottom compartments as filled only with the enzymatically cross‐linkable and degradable 2% w/v PEG‐based hydrogel^[^ ^14^, ^44^ ^]^ used throughout the entire study (Figure S8A–D, Supporting Information). Subsequent computations were executed using the mechanical properties of mature cartilaginous constructs obtained after 14 days of hACs chondrogenic differentiation in the device. The Young's modulus (E) of the hydrogel (1.9 ± 0.5 kPa) and that of mature cartilaginous constructs (3.66 ± 4.16 kPa) were measured through Indentation‐Type Atomic Force Microscopy (IT‐AFM) (Figure 3B). After 14 days of static culture, resulting microtissues exhibited a wider E distribution, indicating a significant amount of newly formed cartilaginous extracellular matrix.

FE simulations estimated that discrete, compartment‐specific confined compression could be achieved. Predicted strains were homogeneous and close to null in the X and Y directions while the average compression in the Z direction resulted, respectively, equal to −2.9% ± 1.6% and −25% ± 7% in the top and bottom constructs (Figure 3C,D; Figure S8E, Supporting Information). The VBV geometry allows therefore to obtain a discrete strain field which, depending on the distance from the actuation membrane, is consistent with the hyperphysiological deformations of cartilage surface associated with the onset of OA,^[^ ^23^, ^48^, ^49^ ^]^ and with the low strain experienced by calcified cartilage and bone in vivo.^[^ ^40^, ^50^ ^]^ Notably, the simulations revealed very limited lateral expansion in both top and bottom constructs. Impeding the lateral expansion (i.e. achieving confined compression) induces an outflow of cartilage's (and cartilaginous constructs’) interstitial fluid, which enhances chondrocytes mechanotransduction due to transient hypo‐osmotic stresses.^[^ ^51^ ^]^

Thereafter, we conducted a conceptual experiment in which the bottom compartment was filled with 2% PEG gel and the top compartment contained constructs with moduli 1, 2, or 10 times higher, based on reports that the macroscale modulus of calcified cartilage at the osteochondral interface is over 10 times greater than that of the overlying cartilage (at least in bovines^[^ ^4^ ^]^). Independently from the constructs’ mechanical properties, their deformations remained stable and discrete between the top and bottom compartments (Figure 3E), demonstrating the robustness of our strain‐controlled technology.

Numerical results were then validated experimentally by investigating whether cartilaginous constructs under compartment‐specific compression in the top and bottom compartments exhibited distinct biological responses (Figure 3F). To this aim, GFP+ hACs and non‐labeled hACs were embedded in 2% w/v PEG hydrogels, and respectively seeded in the bottom and top compartments (or vice‐versa, to exclude possible donor‐ or transduction‐dependent effects). hACs were used in both chambers to ensure that disparities in cellular responses depended solely on the different compression levels experienced by the constructs in top and bottom compartments rather than from intrinsic differences between cell types.

After two weeks of chondrogenic culture, chondrocytes formed compact tissues in both compartments while maintaining a well‐defined bi‐layer structure (Figure S9A,B Supporting Information), and showed a significant upregulation of genes associated with hyaline cartilage (Figure S9C, Supporting Information). We previously demonstrated that these characteristics are necessary to obtain clinically relevant load responses.^[^ ^14^ ^]^ Cyclic compression was applied following the loading pattern detailed in Figure 3F. Cyclical deformation of the actuation membrane was achieved by connecting the devices to a compressed air source through a pressure regulator, employing a setup that we have described previously.^[^ ^52^ ^]^ At the end of the experiment, constructs were enzymatically digested and cells were sorted based on GFP expression, to independently assess the gene expression of the two compartments (Figure S10A–C, Supporting Information).

Following cyclic compression, a significant upregulation of *FOS* was registered for hACs isolated from both compartments with respect to static controls (*p*‐values in black in Figure 3G). Moreover, the expression of the transcription factor *C‐JUN*, which is involved in articular cells’ fate specification,^[^ ^53^ ^]^ and that of the OA‐associated, collagen‐degrading enzyme *MMP13* (which is differently expressed in cartilage surface and deep zones, Figure 1D) were significantly increased in hACs from the bottom compartment with respect to those of the top one (p‐values in red in Figure 3G). *FOS* and *C‐JUN* are established mechanotransduction markers and belong to the signaling pathway of the transient receptor potential vanilloid 4 (TRPV4), which has a key role in the physiologic response of articular cartilage to loading.^[^ ^51^ ^]^ These modulations of mechanoresponsive genes suggest therefore that cyclic compression affects both compartments, with compartment‐specific effects due to varying compression levels. Of note, the gene expression of constructs cultured in top and bottom compartments of static controls did not differ (Figure S11A, Supporting Information), excluding a possible geometry‐dependent effect, nor it depend on GFP‐expression (Figure S11B, Supporting Information), excluding effects due to transduction operations.

By taking advantage of the diffusion‐delaying top compartment geometry, we also measured the compartment‐specific release of IL8, an inflammatory cytokine involved in OA inflammatory processes. Upon loading, a significantly higher concentration of IL8 was measured in the culture medium collected from the bottom (and not from the top) compartment (Figure 3H), further confirming the induction of different biological processes.

In summary, FE numerical models indicate that the mechanical stimulations applied by the proposed device closely mimic the compression levels experienced by OCU tissues in vivo. Additionally, differences in gene expression and cytokine release between hACs cultured in top and bottom compartments demonstrate that the proposed platform effectively captures tissue‐specific responses to these distinct mechanical stimulations.

### 2.3 Subchondral Layer's Mechanical Properties Affect hACs Response to Loading

Next, as a last functional validation, we investigated if the OCU‐on‐Chip device could be used to examine, in vitro, how the mechanical properties of the subchondral layer affect cartilaginous constructs’ loading response (Figure 3I).

For this purpose, we conducted an experiment in which the bottom compartment was seeded with hACs‐laden PEG hydrogels, while the top compartment was filled with PEG hydrogels loaded with polystyrene beads with volumetric fractions of 0.1% (0.1% B) or 5% (5% B), respectively (Figure 3I; Figure S12A, Supporting Information). The stiff plastic beads (E modulus of 3250 MPa^[^ ^54^ ^]^) alter the hydrogel's local E value proportionally to their volumetric fractions (Figure S12B,C, Supporting Information). These were purposely selected so that the E modulus of 0.1%B and 5%B hydrogels approximated, respectively, the median E modulus of glycosaminoglycan (GAG)‐rich and GAG‐poor areas at the osteochondral interface in biopsies from OA patients (Figure S12D–F, see Supplementary Note S3, Supporting Information for details).

Upon HPC, a higher volumetric fraction of beads in the subchondral layer led to hACs overexpressing the inflammatory cytokine *CXCL8* (both with respect to static controls and comparing the 0.1% B and the 5% B substrates, Figure 3J) and to an increase in the release of the degradative and inflammatory factors proMMP13, IL8, and IL6, with the concentration of IL8 and IL6 being significantly higher for the 5% B substrate (Figure 3K).

These results are in accordance with clinical observations. OA has been associated with changes in the structure,^[^ ^5^ ^]^ mechanical properties,^[^ ^55^ ^]^ and extent of mineralization of calcified cartilage and subchondral bone^[^ ^1^, ^32^ ^]^ that, in turn, are hypothesized to drive the degradation of the overlaying cartilage.^[^ ^29^ ^]^ In vivo, however, these effects cannot be distinguished from those mediated by molecular signaling between cartilage and subchondral bone. By using plastic substrates and exerting strict control over experimental variables in a purposely oversimplified system, we decoupled the effect of mechanics from those of inter‐tissues cross‐talks. These outcomes showcase how the OCU‐on‐Chip device can be employed to interrogate the effects of individual elements in OCU degeneration.

### 2.4 Establishment of Osteochondral Biphasic Cellular Constructs On‐Chip

Once we had validated the technological platform, as a further step toward a proper OCU‐on‐Chip model, we aimed to achieve a biphasic 3D cellular construct effectively mimicking the cellular and extracellular matrix (ECM) constitution of both hyaline cartilage and mineralized osteochondral interface.

For the cartilage surface layer, we adopted hACs, which we demonstrated to be capable of forming hyaline cartilage‐like micro constructs in our CoC;^[^ ^14^ ^]^ for the subchondral mineralized layer, we used bmMSCs, which are inherently committed to endochondral ossification.^[^ ^56^ ^]^ Both cell types were primary and obtained from donors with no history of OA (See Methods for details). Both constructs were obtained by embedding cells in an enzymatically degradable, PEG‐based hydrogel^[^ ^45^ ^]^ that promotes cartilage formation by competent cells.^[^ ^14^ ^]^

**Figure 4:**
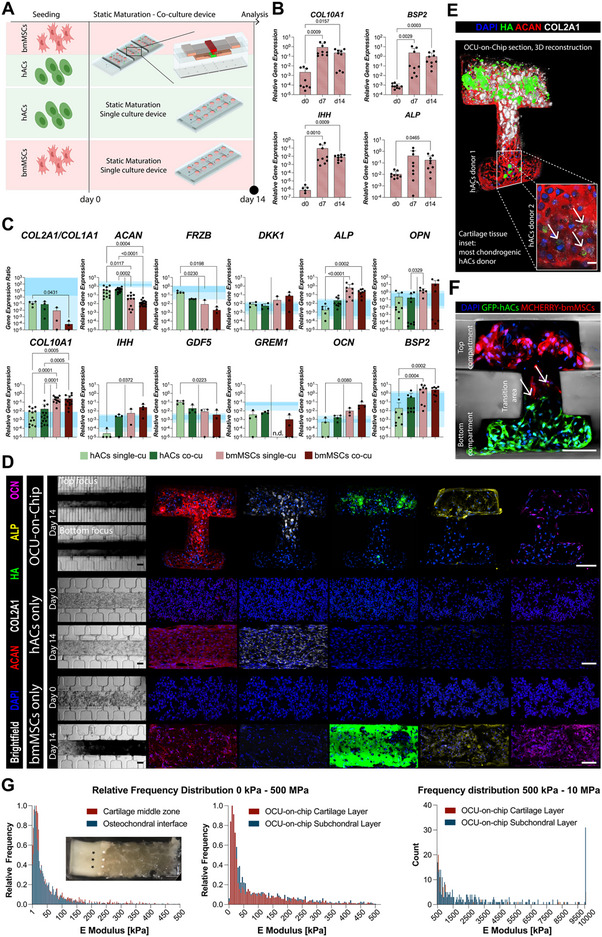
Achievement of bi‐phasic osteochondral cellular constructs on‐chip. A) Schematic of the OCU‐on‐Chip maturation experiment. B) bmMSCs maturation in single culture. Gene expression was quantified through RT‐qPCR (*n* = 9 independently cultured samples from *n* = 3 donors). Statistics by Kruskal‐Wallis test with Dunn's post hoc test for multiple comparisons. Expression levels of all genes were normalized to GAPDH expression. Values are reported as mean + s.d. C) Assessment of hACs and bmMSCs gene expression in single‐ and co‐culture. OCU‐on‐Chip constructs composed of GFP hACs and bmMSCs were enzymatically digested and sorted, and the two cell populations were analyzed separately. Results were also referred to the gene expression of cartilage surface and deep zone/calcified cartilage from human knee samples (darker color = average range, lighter color = standard deviation range) (*n* = 4 independently cultured samples from *n* = 2 bmMSCs donors and *n* = 1 GFP+ hACs donor). Statistical significance by Kruskal‐Wallis test with Dunn's post hoc test for multiple comparisons. Population normality was assessed through Shapiro‐Wilk and Kolmogorov‐Smirnov tests. (Adjusted) *p*‐values < 0.05 are reported on the graph. Expression levels of all genes were normalized to GAPDH expression. Values are reported as mean + s.d. D) Brightfield and immunofluorescence images of single cultures (top view) and OCU‐on‐Chip constructs (sections) (*n* ≥ 6 independently cultured samples from *n* ≥ 3 donors/experiments). Scale bar, 100 µm. E) 3D confocal reconstruction of an OCU‐on‐Chip section after 14 days of static maturation. The inset represents the same area highlighted in the main figure but from a different donor. Arrows in the 2D inset point to HA‐positive cells in the bottom compartment. Scale bars: section 100 µm, inset 10 µm. F) Immunofluorescence image of osteochondral constructs composed of GFP+ hACs and mCherry bmMSCs after 14 days of static maturation (*n* = 6 independently cultured constructs). Migrating cells in the transition area are indicated by arrows. Scale bar, 100 µm. G) Young's modulus of osteochondral biopsies (left) and of OCU‐on‐Chip samples (right) after 14 days of maturation as determined by IT‐AFM. Histograms represent the number (i.e. the count) of indentation spots that resulted in having a Young's modulus of a given value. The relative frequency was obtained by dividing the count per value by the total count. The inset illustrates an example of an osteochondral biopsy. White and black asterisks indicate, respectively, representative indentation areas for the osteochondral interface and hyaline cartilage. Scale bar 1 mm. In the 500 kPa −10 MPa range graph on the right, values >10 MPa were aggregated. *n* = 4 donors were considered for osteochondral biopsies, *n* = 3 donors for OCU‐on‐Chip samples. *n* = 2 independently cultured constructs were considered for each donor.

The experimental setup is depicted in **Figure** **4**A, bi‐phasic OCU‐on‐Chip constructs were compared to single culture controls (using our published CoC platform^[^ ^14^ ^]^).

Constructs were cultured with an osteochondral medium (OCM) containing both chondro‐inductive factors and beta‐glycerophosphate (β‐GP), which serves as a source of inorganic phosphate when hydrolyzed by alkaline phosphatase (ALP) during ossification processes.^[^ ^57^ ^]^ The OCM formulation was optimized (using both our single‐culture CoC platform and cell aggregates models, Figure S13, Supporting Information) so that (i) it induced a significant upregulation of hypertrophy (*COL10A1* and *IHH*), and ossification (*ALP* and *BSP2*) markers in bmMSCs, which formed mineralized constructs (Figure 4B; Figure S13A–C, Supporting Information), while (ii) it did not affect hACs’ chondrogenic capacity and gene expression with respect to an established chondrogenic medium (CHM^[^ ^58^ ^]^) (Figure S13A–E, Supporting Information).

Bi‐layered OCU‐on‐Chip constructs cultured in OCM were characterized after 14 days of differentiation in static conditions. First, we verified whether the two cellular populations maintained well‐distinct gene expression signatures. The gene expression of hACs and bmMSCs retrieved either from single culture or co‐culture samples were analyzed separately and compared to the ones of respective clinical samples' counterparts (reported in Figure S1, Supporting Information). RT‐qPCR did not allow to detect differences in gene expression between single‐ and co‐cultures. Modulations in the expression of the *COL2A1/COL1A1 ratio* and of the hypertrophic brake *GREM1* were detected comparing bmMSCs from single and co‐cultures, and an increased expression of the hypertrophy marker IHH was registered for hACs. Differences were however not significant and expression values were in range with clinical references (blue band in Figure 4C). Significant differences were instead observed between co‐cultured hACs and bmMSCs (Figure 4C). hACs expressed higher levels of the chondrogenic markers *COL2A1/COL1A1* and *ACAN*, which (together with the cartilage markers *SOX9, COMP*, and *COL3A1*) increased more than two orders of magnitude with respect to their levels before the co‐culture on‐chip (i.e. the level of 2D expanded primary hACs at Day 0, Figure S14, Supporting Information) and reached in vivo levels (blue bands in Figure 4C; Figure S14, Supporting Information). bmMSCs expressed higher levels of the hypertrophy markers *COL10A1* and IHH, lower levels of the Wnt antagonist *FRZB* (which was defined as a hypertrophy brake^[^ ^35^, ^59^ ^]^), and higher levels of the ossification markers *BSP2*, *ALP*, and *OPN* (Figure 4C). These latter were comparable (or even higher) to respective expression levels in the calcified cartilage of patients (which cannot be completely distinguished from that of cartilage deep zone when processing tissue biopsies).

We then investigated the constructs’ structural composition. OCU‐on‐Chips were characterized by a dark mineralized matrix observable through brightfield imaging (Figure 4D, left) and were therefore analyzed considering vertical sections to visualize the inter‐tissue interface. Immunofluorescence images (Figure 4D,E) revealed the formation of osteochondral‐like constructs with a cartilaginous layer (derived from hACs, bottom compartment) positive for ACAN and COL2A1 (whose positivity remained also in the bmMSCs layer), and a mineralized layer (derived from bmMSCs, top compartment) positive for hydroxyapatite (HA) and for the osteoblasts markers ALP and osteocalcin (OCN).^[^ ^60^ ^]^ This composition well represents the one of clinical osteochondral biopsies (Figure 1 B,C) and it is consistent with previous results of macroscale osteochondral constructs obtained by the co‐culture of animal‐derived hACs and bmMSCs.^[^ ^61^ ^]^

A co‐culture‐mediated effect could be detected. hACs‐based cartilaginous constructs in a single culture remained completely negative for mineralization (Figure 4D), while HA‐positive spots were detectable in the cartilaginous layer of co‐cultures (Figure 4D,E). The phenomenon was evident even with hACs from highly chondrogenic donors (Figure 4E, inset). To assess if this was due to cell migration or co‐culture‐mediated hACs differentiation, we repeated the experiment using GFP+ hACs and mCherry bmMSCs. Evidence of cell migration between compartments could only be detected in the transition area (i.e. the VBV striction, Figure 4F). Therefore, HA‐positive cells located deep within cartilaginous constructs are most likely hACs which underwent phenotypical changes induced by an active crosstalk. This result aligns with the pathological cartilage calcification observed in OA.^[^ ^36^ ^]^ Notably, although no OA‐inducing stimulus was applied during maturation, our hydrogel‐based constructs are likely more diffusion‐permissive than healthy cartilage, facilitating the cartilage‐osteochondral interface crosstalk hypothesized to play a role in calcification processes in OA.^[^ ^1^, ^36^, ^62^ ^]^

Finally, we used IT‐AFM to compare the nano/microscale mechanical properties of the OCU‐on‐Chip with those of respective equivalents in osteochondral biopsies taken from the femoral condyles of patients undergoing knee replacement (Figure 4G). Native hyaline cartilage and osteochondral interface demonstrated Young's modulus distributions centered around 20–30 kPa, lower values than those reported for macroscale samples (0.8‐1.3 MPa for cartilage,^[^ ^63^, ^64^ ^]^ 11–14 GPa for subchondral bone^[^ ^65^ ^]^). This is consistent with the fact that the macroscale mechanical properties of biological tissues depend both on the properties of their constituent materials and on their hierarchical organization. Instead, nanoscale IT‐AFM assesses only the constituents’ properties. Our findings are in line with IT‐AFM values previously reported for proteoglycans.^[^ ^64^ ^]^

Both cartilaginous and subchondral OCU‐on‐Chip layers demonstrated E distributions well in accordance with that of clinical counterparts. Moreover, indentation spots with E values higher than 10 MPa, possibly corresponding to HA crystals, could be registered for the mineralized subchondral layer.

Collectively, these findings indicate the achievement of mature OCU‐like bi‐layer constructs with gene expression, ECM constitution, and microscale mechanical properties approaching those of native tissues, and point toward a cross‐talk between hACs and bmMSCs.

### 2.5 Hyperphysiological Compression Induces Synergistic Responses in OCU‐on‐Chip Layers and Drives OA‐Like Chondrocalcinosis

OA is characterized by changes in the whole OCU, which receiving and dissipating the stresses associated with movement and loading is continuously biomechanically challenged.^[^ ^1^, ^66^ ^]^ We set to discriminate if hyperphysiological, compartment‐specific loading could elicit OA traits in OCU‐on‐Chip constructs.

**Figure 5:**
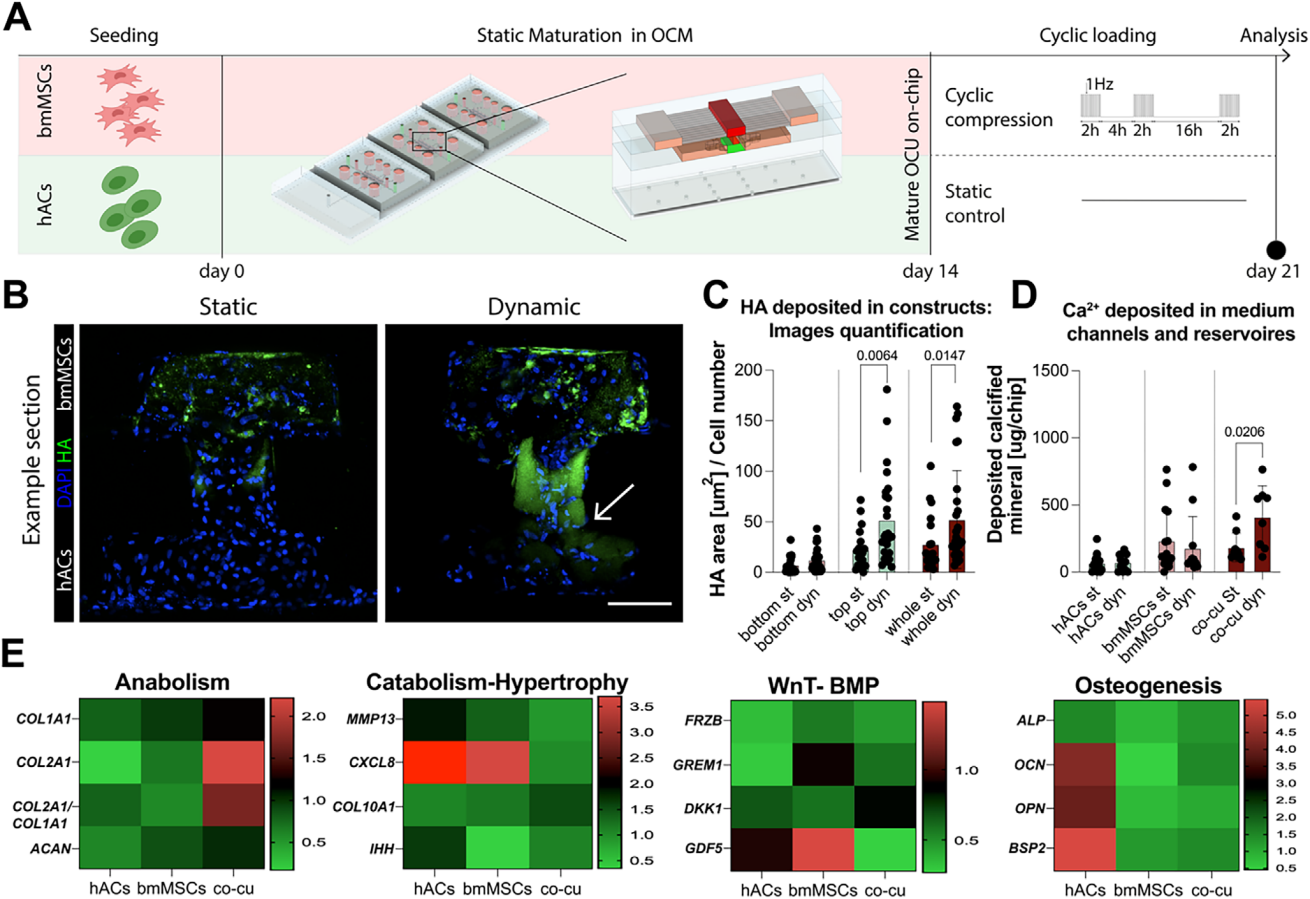
Hyperphysiological cyclical loading elicits synergistic responses in OCU‐on‐Chip layers. A) Experimental plan schematic. The same experiment was performed with hACs and bmMSCs cultured separately (i.e., in single culture) in the CoC device. B) Representative immunofluorescence images of sections of static (left) and cyclically loaded (right) OCU‐on‐Chip constructs at day 21. hACs‐based cartilaginous constructs are located at the bottom, bmMSCs‐based mineralized tissues at the top (n≥20 sections from *n* = 3 hACs and bmMSCs donors/experiments). The white arrow points to a calcified cartilage region. Scale bar, 100 µm. C) Quantifications of HA stainings in OCU‐on‐Chip constructs. Top areas were considered comprehensive of the VBV necking area. Statistical significance between static and cyclically loaded constructs was determined by two‐tailed unpaired *t*‐test for normal populations and by Mann‐Whitney test for non‐gaussian populations (*n* ≥ 20 sections from *n* = 3 hACs and *n* = 3 bmMSCs donors/experiments). Values are reported as mean ± s.d. D) Quantification of calcium deposits in culture medium channels and reservoirs as determined by alizarin red stainings (*n* ≥ 9 independently cultured samples from *n* = 3 experiments). Statistical significance between static and cyclically loaded samples was determined by two‐tailed unpaired *t*‐test for normal populations and by Mann‐Whitney test for non‐gaussian populations. Values are reported as mean ± s.d. E) Comparison of the effect of cyclic mechanical loading on hACs and bmMSCs single cultures and on OCU‐on‐Chip constructs. All tissues were cultured statically in OCM for 14 days and then subjected to cyclic mechanical loading for 7 days. Mechanical compression was applied with the pattern described in A. Single cultures were performed using our previously published COC device.^[^ ^14^ ^]^ The gene expression of OCU‐on‐Chip constructs was analyzed without separating cartilaginous and subchondral layers. Heatmaps represent the fold change in gene expression of cyclically loaded samples with respect to static controls (*n* = 9 independently cultured samples from *n* = 3 hACs donors and *n* = 3 bmMSCs donors). In all cases, population normality was assessed through Shapiro‐Wilk and Kolmogorov‐Smirnov tests.

First, we assessed whether HPC influenced the deposition of calcium phosphate crystals. Changes in subchondral bone mineral content have been reported both in OA animal models^[^ ^67^ ^]^ and in OA patients.^[^ ^1^, ^36^ ^]^ OA was also associated with chondrocalcinosis^[^ ^36^ ^]^ and hyper‐calcification of calcified cartilage.^[^ ^32^ ^]^ Some authors posited that calcium particles function as stress concentration points, preceding and driving cartilage degeneration.^[^ ^4^, ^31^, ^32^, ^55^ ^]^ For this purpose, cyclic HPC was applied on mature OCU‐on‐Chip constructs with a stimulation pattern that we previously showed to induce OA traits in cartilage microtissues without inducing direct cell death^[^ ^14^ ^]^ (**Figure** **5**A), thus mimicking degeneration rather than acute injury.

In accordance with clinical data,^[^ ^1^, ^36^, ^67^ ^]^ immunofluorescence imaging (Figure 5B) revealed a significant increase in the HA content of OCU‐on‐Chip's top compartment upon HPC (HA‐area/cell‐number – average ± Std.Dev – bottom_st_ = 7.3µm^2^ ± 8.5µm^2^; bottom_dyn_ = 12.3 µm^2^ ± 11.6 µm^2^; top_st_ = 22.3 µm^2^ ± 19.9 µm^2^; top_dyn_ = 51.8 µm^2^ ± 44.4 µm^2^). We could also observe cases in which the mineralization front showed an advancement into the cartilage layer (Figure 5B, Figure S15A, Supporting Information). Furthermore, while the overall deposition of COL2A1 and ACAN was not significantly diminished due to loading (Positive‐area/cell‐number – average ± Std.Dev. – COL2A1: bottom_st_ = 134.9 µm^2^ ± 152.1 µm^2^; bottom_dyn_ = 76.2 µm^2^ ± 91.8 µm^2^; top_st_ = 202.2 µm^2^ ± 219.7 µm^2^; top_dyn_ = 142.7 µm^2^ ± 116.0 µm^2^; ACAN: bottom_st_ = 196.2 µm^2^ ± 112.3 µm^2^; bottom_dyn_ = 167.6 µm^2^ ± 79.8 µm^2^; top_st_ = 117.9 µm^2^ ± 93.2 µm^2^; top_dyn_ = 183.3 µm^2^ ± 119.8 µm^2^;), HA positive cartilage areas were negative for both COL2A1 and ACAN (Figure S15A,B, Supporting Information). These observations reflect the chondrocalcinosis and the progression of calcified cartilage toward hyaline articular cartilage detected in osteochondral biopsies from OA patients.^[^ ^32^, ^36^ ^]^ Additionally, we registered a significant increase in the amount of calcium salts released in the culture medium and deposited in medium channels and reservoirs of mechanically stimulated samples (Figure 5D). This phenomenon, which is consistent with the detection of calcium crystals in the synovial fluid of OA patients,^[^ ^68^ ^]^ was only observed with OCU‐on‐Chip constructs, not with single culture counterparts (Deposited calcium salts/chip – average ± Std.Dev – hACs_st_ = 65.7 µg ± 64.6 µg; hACs_dyn_ = 71.3 µg ± 60.47 µg; bmMSCs_st_ = 232.0 µg ± 239.8 µg; bmMSCs_dyn_ = 177.2 µg ± 235.3 µg; co‐cu_st_ = 182.6 µg ± 106.9 µg; co‐cu_dyn_ = 411.2 µg ± 231.6 µg) thus excluding that observed modulations were simply due to bmMSCs’ physiological responses to loading.

Of note, in our strain‐controlled OoC platform, exclusively the cartilage layer is subjected to HPC, the subchondral layer is exposed to an almost null compression level. These data highlight therefore a synergistic response of OCU tissues to aberrant mechanical stimuli.

To further highlight this interplay, we compared the loading response of the OCU‐on‐Chip to one of hACs’ and bmMSCs’ single cultures subjected to the same cyclical HPC. Figure 5E depicts a heatmap of the fold change in gene expression of loaded constructs with respect to model‐specific static controls (non‐normalized values are reported in Figure S16, Supporting Information). Single culture cartilaginous constructs responded to hyperphysiological loading as previously reported in our CoC model,^[^ ^14^ ^]^ with a decrease in anabolism markers and an increase in inflammatory, degradative, and hypertrophic markers. We also observed an increase in the fold change of the osteoblast markers *BSP2* and *OCN*. However, hACs’ *OCN* and *BSP2* expression remained three orders of magnitude lower than that of bmMSCs or OCU co‐cultures (Figure S16, Supporting Information). Loading had a minimal effect on bmMSCs in a single culture, limited to a decrease in *IHH* expression.

OCU‐on‐Chip constructs expressed a high level of hypertrophy and bone markers due to the presence of the subchondral layer and, upon compartment‐specific HPC, exhibited an overall upregulation of anabolic (e.g. *COL2A1/COL1A1* ratio) and hypertrophy (e.g. *IHH*) markers. At the same time, they showed a downregulation of the chondrogenic stem cell marker *GDF5* (Figure S16, Supporting Information), which characterizes synovial progenitors involved in osteochondral defect repair mechanisms.^[^ ^69^, ^70^ ^]^ Hence, co‐cultures demonstrated a distinct response to loading that was different from the simple average of single‐culture controls.

These observations – while preliminary – might indicate that the OCU‐on‐Chip reflects an early OA signature, characterized by an initially anabolic response that transitions into a degenerative process when coupled with chondrocytes hypertrophy, depletion of progenitor markers, and alterations of subchondral bone mineral content. Upregulation of *COL2A1* was previously observed in a mechanically induced OA animal model^[^ ^71^ ^]^ and COL2A1 characterizes the osteochondral interface of clinical biopsies (Figure 1C). Moreover, while OA is characterized by overall cartilage erosion, an increase in anabolic processes in the initial phases of the pathology has been reported.^[^ ^66^ ^]^

Overall, these results indicate that the OCU‐on‐Chip captures features of the cartilage‐subchondral tissues interplay happening in the response to pathological loading, and validate it as an effective model of pathological calcification processes.

### 2.6 hACs Sub‐Populations with Distinct Transcriptomic Profiles Characterize Loaded Cartilaginous and OCU‐on‐Chip Models

OA pathogenesis was recently connected to the lineage progression patterns of chondrocytes with distinctive roles and transcriptomic profiles.^[^ ^3^, ^72^, ^73^ ^]^ Moreover, promising cartilage repair strategies seem to depend on specific joint resident progenitor populations.^[^ ^69^, ^74^ ^]^

**Figure 6:**
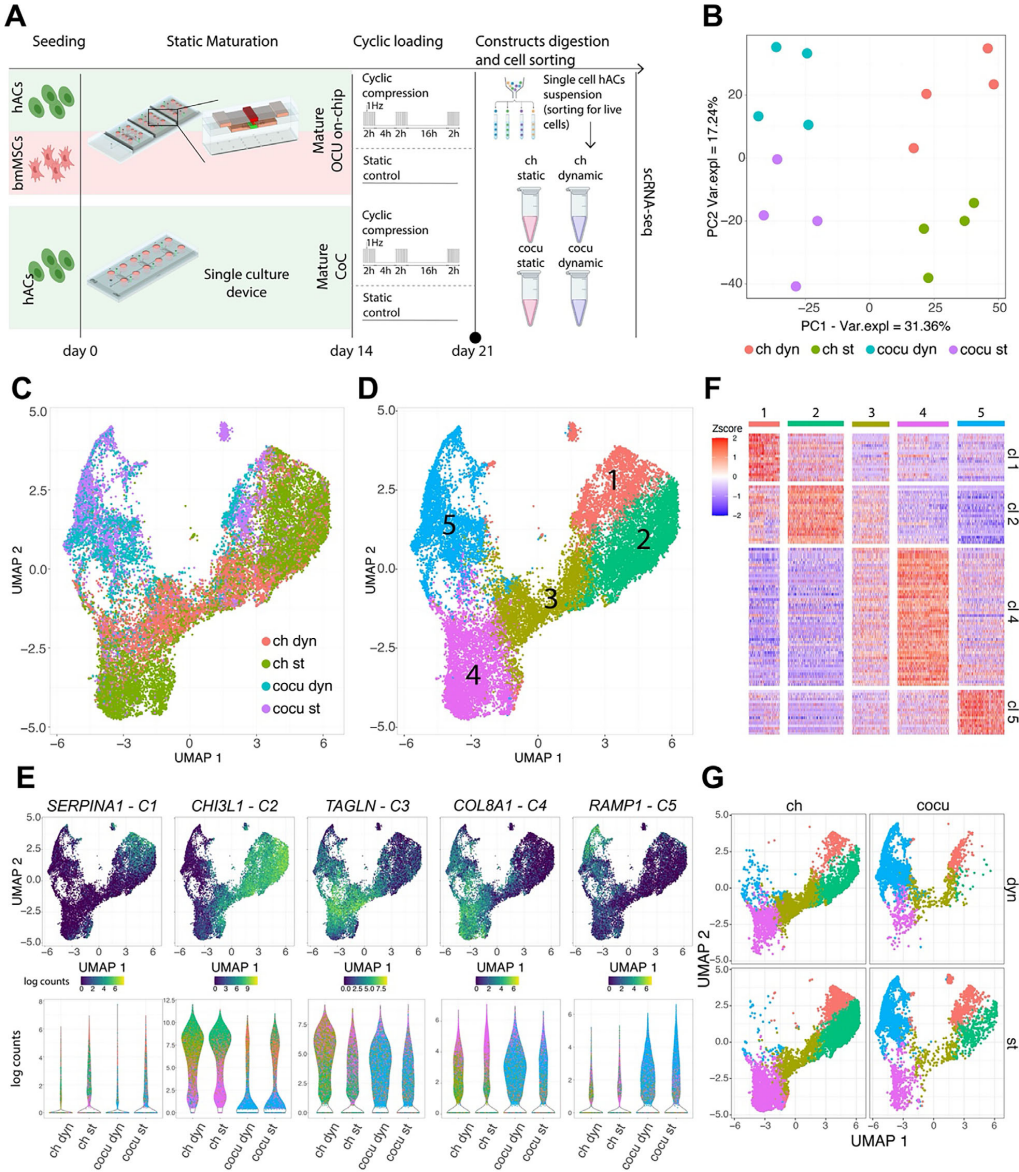
Co‐culture in OCU‐on‐Chip and hyperphysiological compression affect hACs subpopulations. A) Schematic of the scRNA‐seq experimental procedure. Cells were retrieved from OCU‐on‐Chip constructs through enzymatic digestion and enriched for live cells through FACS sorting. scRNA‐seq was performed on hACs only. B) PCA plot of in silico bulk samples. Each dot represents one of the 4 donors considered in scRNA‐seq analyses. Var.expl = variance explained. C) UMAP embedding of analyzed hACs colored according to stimulation state (i.e. static vs dynamic) and culture conditions (i.e. single vs co‐culture). D) UMAP embedding of analyzed hACs. The color code indicates the different cell clusters. E) Feature and violin plots of selected cluster markers. Data points referring to single cells in violin plots are colored according to the clusters’ color scheme of D. F) Heatmap reporting the Z‐score of differentially expressed genes in clusters defined in D. While specific cluster markers could be defined for clusters 1, 2, 4, and 5, cluster 3 had an intermediate state between cluster 2 and 4, without characteristic marker genes. Genes are represented in rows; columns refer to the gene expression of single cells. G) UMAP embedding representing cell clusters divided by stimulation state (static vs dynamic) and culture conditions (single vs co‐culture). Cells are colored based on their assigned clusters following the color scheme of panel D.

In this framework, and considering that our results suggested the presence of a crosstalk between tissue layers, we sought to determine the effects of co‐culture and compartment‐specific HPC on the diversity of hACs’ subpopulations. For this purpose, we performed single‐cell RNA sequencing (scRNA‐seq) on hACs from CoC and OCU‐on‐Chip cultures and exposed, or not, to cyclic (compartment‐specific) HPC (**Figure** **6**A). Constructs were subjected to an enzymatic digestion optimized to selectively isolate hACs (see Experimental Section). Additionally, residual bmMSCs were identified based on donors’ single nucleotide polymorphisms (SNPs) and excluded from downstream analyses (see Supplementary Methods). A total of 24579 cells from 4 human donors passed quality control steps and underwent further analysis. Mapping and counting statistics are reported in Figure S17A,B (Supporting Information). Gene expression was corrected for the individual donor effect (details in Supplementary Methods).

First, we performed Principal Component Analysis (PCA) on in silico bulk samples (i.e. samples obtained by summing up counts per gene across all cells from each condition), revealing that PC1 correlated with the culture condition (i.e. single‐ or co‐culture), while PC2 with the stimulation state (i.e. static or dynamic) (Figure 6B). We defined the top 100 genes contributing the most, either positively or negatively, to PC1 and PC2, and subjected them to Gene Ontology (GO) analysis^[^ ^75^, ^76^ ^]^ (Data S1, Figure S17C,D, Supporting Information). Biological Processes (BPs) enriched in genes aligned with PC1 included Skeletal System Development, Ossification, and Cartilage Development (PC1 negative), or Collagen Fibril Organization, Regulation of Cell Migration, and Regulation of Cell Population Proliferation (PC1 Positive). BPs enriched in genes aligned with PC2 included Defence Response, Response to Stress, and Inflammatory Response (PC2 negative), or Cell Cycle, Mitotic Cell Cycle, and Cell division (PC2 Positive). These data further validate that our model captures essential processes related to the OCU's mechanical pathophysiology.

Next, cells from all four experimental conditions were visualized together in the form of Uniform Manifold Approximation and Projection (UMAP) (Figure 6C), and cell clustering was applied to determine if hACs subpopulations were present in the dataset. Five cell clusters could be distinguished based on differential gene expression (Figure 6D). Cluster marker genes and associated GO terms for BPs are reported in Figure S18A,B, and Data S2 (Supporting Information). Feature and violin plots of selected marker genes are reported in Figure 6E. Among all differently expressed genes across clusters (See Supplementary Data 2 for a complete list) the following genes were of particular interest. Cluster 1 was characterized by an elevated expression of the genes *SERPINA1*, *SERPINA5*, *SERPINE2*, and *TNFAIP6*, which have been reported among the most upregulated genes in OA cartilage.^[^ ^77^ ^]^ Cluster 2 was marked by an elevated expression of *CHI3L1* and *CHI3L2*, which distinguish a specific chondrocyte subpopulation: Regulatory chondrocytes.^[^ ^72^, ^73^ ^]^ There was no marker gene uniquely assigned to cluster 3, consisting of cells expressing markers such as TAGLN, which were also expressed by clusters 2 and 4, indicating that cluster 3 was constituted by cells differentiating between these two clusters. Cluster 4 was characterized by marker genes such as *COL1A1*, *COL1A2*, *COL8A1*, and *FBN1*, thus representing a more fibroblastic or dedifferentiated chondrocyte phenotype. Finally, cluster 5 was categorized by *UNC5B*, *RAMP1, ENPP1*, and *S100A1*, associated with GO terms such as “Inorganic Diphosphate Transport”, “System Development”, and “Multicellular Organismal process”, which are linked to ossification processes.

Of note, while different clusters could be defined, hACs’ transcriptome indicated the presence of gradually differentiating cells rather than completely distinct populations (Figure 6F), in alignment with scRNA‐seq analyses of clinical samples.^[^ ^72^, ^73^, ^78^ ^]^

A clear correlation between clusters and culture conditions was detected (Figure 6G). The distribution of hACs from the different conditions across clusters is reported in Figure S18C (Supporting Information). hACs from OCU‐on‐Chip co‐cultures were mainly found in clusters 1 and 5, while cells from the CoC model characterized clusters 2, 3, and 4. hACs from HPC stimulated OCU‐on‐Chip constructs were assigned to cluster 5 in percentages ranging from 45% to 70%, depending on the donor (Figure S18C, Supporting Information), while co‐culture (slightly) and dynamic loading (majorly) seemed to have a depleting effect on cluster 2.

Collectively, these data provide a description of the transcriptomic profile of hACs in our model, which captures biological processes active in OCU pathophysiology, and reveals that hACs from the OCU‐on‐Chip co‐culture gave rise to a unique cell subpopulation not found in the CoC. Thus, scRNA‐seq analyses substantiate the presence of a cross‐talk between cartilage and subchondral layer in the OCU‐on‐Chip.

### 2.7 OCU‐on‐Chip Culture Better Preserves the Pathophysiological Variability of Chondrocytes Subpopulations In Vivo

Thereafter, we compared the gene signature of subpopulations highlighted in our dataset with the transcriptome of cartilage cells from OA patients^[^ ^72^, ^79^ ^]^ and healthy subjects.^[^ ^73^ ^]^

**Figure 7:**
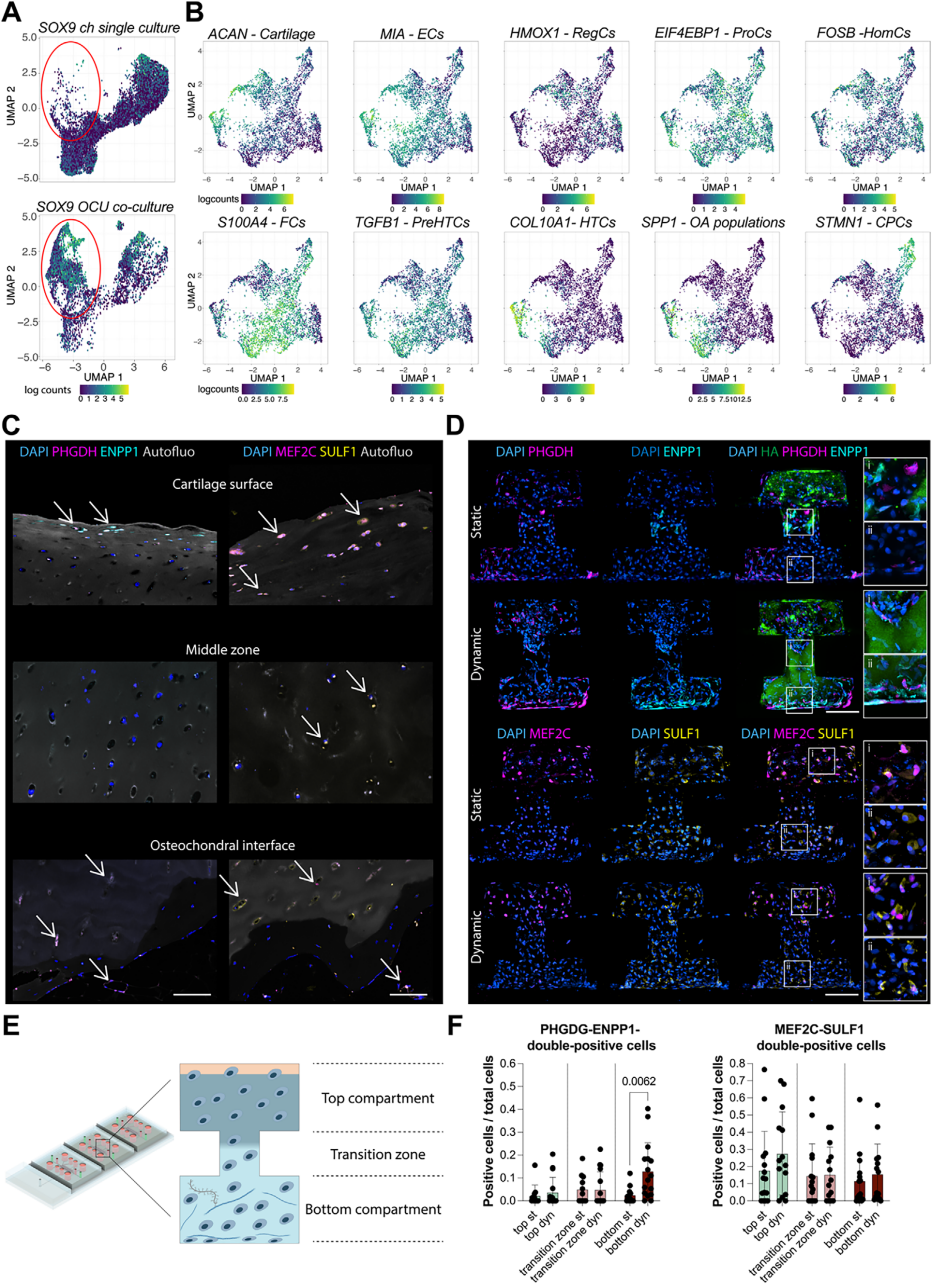
Characterization of hACs subpopulations and co‐culture dependent clusters. A) Feature plots of the classic chondrogenic markers *SOX9*. Panels are divided by culture conditions (CoC vs OCU‐on‐Chip). The red ellipses highlight a co‐culture‐dependent population highly expressing *SOX9*. B) Feature plots of marker genes of hACs subpopulations in re‐clustered cells from cluster 5. C) Immunofluorescence images of osteochondral biopsies taken from the distal femur of OA patients undergoing total knee arthroplasty (*n* = 2 sections from more degraded and more preserved areas for each of *n* = 4 donors). Scale bars, 100 µm. D) Localization of cells expressing cluster 5 markers in OCU‐on‐Chip constructs. Immunofluorescence images of hACs and bmMSCs co‐cultured for 14 days in OCM and then subjected (Dynamic) or not (Static) to 7 days of cyclical mechanical stimulation (*n* ≥ 2 independently cultured samples from *n* = 3 hACs and *n* = 3 bmMSCs donors). Scale bar, 100 µm. E) Schematic representation of an OCU‐on‐Chip section subdivided into the areas used for image quantification. F) Quantification of double‐positive PHGDH‐ENPP1, and double‐positive MEF2C‐SULF1 cells in OCU‐on‐Chip constructs. Statistics by two‐tailed unpaired *t*‐test for normal populations and by Mann‐Whitney test for non‐gaussian populations. Population normality was assessed through Shapiro‐Wilk and Kolmogorov‐Smirnov tests (*n* ≥ 11 sections from *n* = 3 hACs/bmMSCs donors). Values are reported as mean ± s.d.

First, we evaluated classic chondrogenic markers previously used in scRNA‐seq to distinguish hyaline cartilage chondrocytes from other joint cells.^[^ ^78^ ^]^ While *COMP* was universally expressed across clusters (Figure S19, Supporting Information), a significantly higher expression of the chondrogenic markers *SOX9* (**Figure** **7**A), *ACAN*, *COL2A1*, and *COL9A1* was detected in cells from OCU‐on‐Chip co‐cultures, particularly in cluster 5 and, to a lesser extent, in cluster 1 (Figure S19, Supporting Information). This indicates a beneficial effect of the OCU‐on‐Chip co‐culture on hACs’ phenotype.

A pioneer scRNA‐seq study on the cartilage of patients undergoing knee arthroplasty classified hACs into seven subpopulations:^[^ ^72^ ^]^ effector chondrocytes (ECs), regulatory chondrocytes (RegCs), proliferative chondrocytes (ProCs), pre‐hypertrophic chondrocytes (preHTCs), hypertrophic chondrocytes (HTCs), homeostatic chondrocytes (HomCs), and fibrocartilage chondrocytes (FCs). Being based on a restricted patients’ cohort,^[^ ^72^ ^]^ the study was later complemented with scRNA‐seq analyses performed on knee cartilage from healthy subjects, and on chondrocytes from different joints,^[^ ^73^, ^79^, ^80^, ^81^, ^82^ ^]^ which highlighted the presence of further subpopulations, such as chondroprogenitor cells (CPCs).^[^ ^73^ ^]^ It also became clear that hACs represent a continuum of differentiating cells rather than completely different populations, as cluster markers are often ubiquitously expressed, and different authors use the same markers for varied sub‐categorizations.

In this framework, we compiled a list of hACs subpopulations markers merging those of native chondrocytes from healthy individuals^[^ ^73^ ^]^ with those of OA patients,^[^ ^72^ ^]^ and evaluated their expression in our dataset (Figure S20, Supporting Information). A perfect correspondence between our clusters and hACs subpopulations could not be detected, in line with the fact that differences in gene expression induced by culture type (i.e. single‐ vs co‐culture) and stimulation (i.e. static vs HPC) are likely more pronounced than those between hACs subpopulations. However, cells from cluster 1 and cluster 5, enriched in hACs from OCU‐on‐Chip co‐cultures (Figure S18C, Supporting Information), showed an elevated expression of the preHTCs marker genes *TAGLN* and *COL9A3*, and of the HTCs marker genes *WWP2*, *SCIN*, and *S100A1*. Cells from cluster 5 had also an elevated expression of the preHTC marker gene *COL11A1* and of the HTCs marker genes *MEF2C*, *IBSP*, and *COL10A1* (Figure S20, Supporting Information).

A subset of cluster 5 cells showed a high expression of CPCs markers (Figures S20 and S21A, Supporting Information). We verified that this CPCs population was constituted by actively proliferating cells, as indicated by the expression of the proliferation markers *MKI67* and *E2F1* (Figure S21B, Supporting Information) and the assignment of these cells to the S and G2/M cell cycle phases (Figure S21C, Supporting Information), consistently with previously reported self‐renewal and proliferative properties of CPCs.^[^ ^83^ ^]^

The co‐culture‐dependent cluster 5 seemed therefore to possess the highest transcriptional heterogeneity of hACs populations. Accordingly, we isolated cells from cluster 5 and analyzed them separately. Well‐defined hACs sub‐sets expressing chondrocyte sub‐population markers could be highlighted (Figure 7B). These included cells positive for classic chondrogenic markers (e.g., *ACAN*), CPCs markers (e.g., *STMN1*), and previously defined OA hACs subpopulations markers (e.g. *MIA* for Effector chondrocytes (ECs), *FOSB* for HomCs*, TGFβ1* for preHTCs, and *COL10A1* for HTCs). Moreover, we could pinpoint a population of hACs expressing *SPP1*, the gene coding for osteopontin, in accordance with the identification of an OA‐specific population of cells expressing *SPP1* in clinical samples.^[^ ^79^ ^]^

These data indicate that the co‐culture in OCU‐on‐Chip constructs had the bivalent effect of enhancing the expression of classic chondrogenic markers in hACs and enriching them for CPCs, while, at the same time, it induced the differentiation of a subset of cells in OA‐associated preHTCs and HTCs.

Next, we assessed if cells expressing cluster 5 markers (Figure S18; Data S2, Supporting Information) could be localized in osteochondral biopsies from the knee joints of OA patients (Figure 7C; Figure S22, Supporting Information). Given the dependence of the cluster 5 transcriptome on co‐culture, we focused on markers known to be expressed by bmMSCs and that are implicated in hypertrophy and calcification processes, i.e. MEF2C,^[^ ^84^ ^]^ SULF1,^[^ ^85^ ^]^ and ENPP1.^[^ ^86^ ^]^ ENPP1 is specifically involved in chondrocalcinosis processes during OA progression.^[^ ^36^ ^]^ Additionally, we performed staining for the cluster 5 marker PHGDH, an enzyme involved in the conversion of 3‐phosphoglycerate to serine that had previously been connected to OA.^[^ ^87^ ^]^ MEF2c+ and SULF1+ cells were homogeneously distributed across cartilage layers and at the osteochondral interface, while PHGDH+ and ENPP1+ cells were mainly located at the interface between cartilage and subchondral bone, and in the superficial layer. Interestingly, a population of double positive PHGDH+ and ENPP1+ cells was detected on the surface of OA cartilage from mildly degenerated areas (i.e. areas presenting cartilage fibrillation but conserving the superficial zone with flattened chondrocytes) (Figure 7C; Figure S22, Supporting Information).

We sought therefore to localize cells from cluster 5 in OCU‐on‐Chip constructs (Figure 7D), to determine if their distribution reflected that of native samples or if they were mainly located in the transition zone between the top and bottom compartments, likely more exposed to paracrine signals from the bmMSCs‐based subchondral layer. MEF2C+ and SULF1+ cells were present both in the top compartment, as expected for bmMSCs, and deep in the bottom compartment, indicating that possible secreted signals are homogenously diffused in the cartilage layer. The most interesting observation, however, was that PHGDH+ and ENPP1+ double positive cells co‐localized with mineralized cartilage areas both in the transition zone, at the interface with the subchondral layer, and on the cartilage surface in the bottom compartment (Figure 7D), and that such distribution depended on OCU‐on‐Chip constructs being exposed to compartment‐specific HPC (Figure 7E,F). Stainings were quantified by divining OCU‐on‐Chip sections in top compartment, transition zone, and bottom compartment (Figure 7E). PHGDH+ENPP1+ cells/ total cells – average ± Std.Dev: top_st_ = 0.03 ± 0.05; top_dyn_ = 0.04 ± 0.06; transition zone_st_ = 0.05 ± 0.06; transition zone_dyn_ = 0.05 ± 0.08; bottom_st_ = 0.03 ± 0.04; bottom_dyn_ = 0.13 ± 0.12).

Taken together, these data validate the OCU‐on‐Chip as representative of both OA‐associated populations and cells with a more progenitor‐like transcriptome, and indicate that the co‐culture is necessary to mimic the variety of hACs subpopulations found in vivo. Additionally, they imply that, upon loading, the localization in the OCU‐on‐Chip of hACs subpopulations implicated in chondrocalcinosis processes in OA mirrors their positioning in OA osteochondral knee biopsies (considering the limited patients’ cohort that we evaluated).

### 2.8 Analysis of Pathways Dysregulated by Compartment‐Specific HPC

Finally, we set to demonstrate the potential of our approach in providing a comprehensive overview of the cellular processes dysregulated by HPC in hACs.

**Figure 8:**
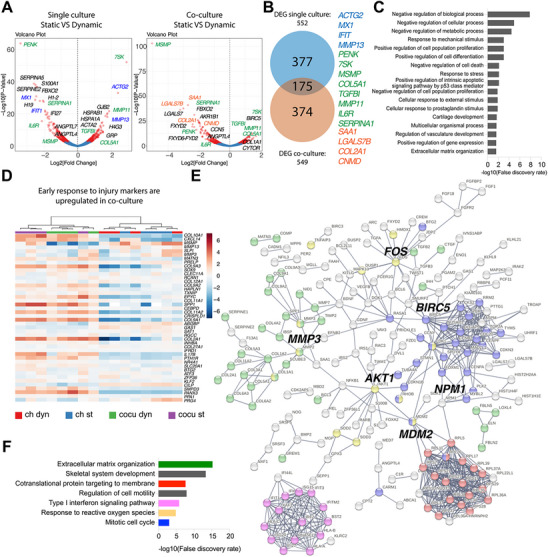
HPC dependent DEGs: pathways and interactions underlying hACs mechanotransduction. A) Volcano plots of DEGs obtained comparing static samples and samples subjected to HPC considering in silico bulk samples from single cultures (left) and co‐cultures (right). DEGs with adjusted *p*‐value < 0.05 are indicated in red. The color of highlighted DEGs refers to genes modulated in both cultures or only in one of the two, as indicated in panel B. B) Common and not common differentially expressed genes in single and co‐culture. A few representative genes are listed on the right. The number of common DEGs is indicated by the overlapping area between the two circles. C) GO terms for BPs related to DEGs in both single and co‐culture (GO terms were obtained through STRING). D) Samples hierarchical clustering based on DEGs 7 days after ACL damage in a mouse model of OA.^[^ ^78^ ^]^ E) STRING‐based protein‐protein interaction network resulting from cluster 5 DEGs. Nodes relative to GO terms highlighted in panel F are represented with the same color code. Nonconnected nodes were excluded from the visualization. Branches in the network are colored according to the interaction evidence (Minimum required interaction score 0.9). F) GO terms for BPs related to DEGs obtained comparing static and HPC samples and considering only cells from co‐cultures of cluster 5. GO terms were obtained through STRING.

To address this question, at first, we applied differential expression (DE) analysis to in silico bulk samples obtained by summing counts per gene across all cells from each condition. CoC and OCU‐on‐Chip samples subjected to cyclic loading were compared to corresponding static controls (**Figure** **8**A). Respectively, 552 and 549 differentially expressed genes (DEGs, FDR<0.05) were detected in single culture and co‐culture, with 175 common DEGs (Figure 6B; Data S3, Supporting Information). KEGG pathways enriched in the two cases are reported in Figure S23 (Supporting Information). We started by focusing on common DEGs that characterized both single‐ and co‐cultures, to highlight processes intrinsically connected to HPC. These comprised genes associated with the onset of hypertrophy (e.g. *FRZB* ^[^ ^35^ ^]^); *TGFBI*, whose signaling is affected by subchondral bone altered architecture in OA;^[^ ^29^ ^]^ and *SERPINE*s, that were among the most modulated genes highlighted in the RAAK study^[^ ^77^ ^]^ (Data S3, Supporting Information). We also detected the modulation of markers of matrix degradation and deposition (e.g. *MMP11*, *COL1A1*, *COL6A2*) including an increase in the expression of *COL5A1*, which correlates with OA progression as reported in a transcriptomic study on clinical samples.^[^ ^72^ ^]^ Corresponding enriched GO terms included Response to Stress, Response to Mechanical Stimulus, and Extracellular Matrix Organization (Figure 8C), validating that both CoC and OCU‐on‐Chip models recapitulate fundamental loading‐induced cartilage responses.

Next, we examined, in our samples, the expression of a set of genes that were modulated seven days post‐intervention in an anterior cruciate ligament injury OA mouse model.^[^ ^78^ ^]^ The rationale for this analysis was to confirm our preliminary assessment of the OCU‐on‐Chip loading response (performed on whole OCU‐on‐Chip constructs, not on hACs alone; see Figure 5E), which appeared to indicate an early OA‐like behavior, characterized by anabolism coupled with the onset of hypertrophy. We could verify this observation. hACs from single cultures and osteochondral constructs clearly clustered separately, and OCU‐on‐Chip‐derived hACs expressed higher levels of ≈50% of early OA markers (Figure 8D).

Subsequently, we analyzed DEGs across clusters. A complete list of genes that were differentially expressed due to HPC in OCU‐on‐Chip and CoC models is provided in Data S3 (Supporting Information); volcano plots with selected DEGs across clusters are reported in Figure S24A (Supporting Information). These proved to be highly variable depending on the cluster, and included genes going from well‐known degradative enzymes (e.g. *MMP3, MMP7*, and *ADAMTSs* in cluster 1, *MMP13* in cluster 4), chemokines related to inflammatory processes (e.g. *CXCL2* and *CXCL3* in cluster 2), and genes associated to TNFα, MAPK, and interferon signaling (in cluster 1, cluster 3, and cluster 4). We could also detect the modulation of *VEGA* and *VEGFC* (cluster 1), which are associated with OA cartilage vascular invasion,^[^ ^4^ ^]^ despite not having vascular cells in our model.

These data establish the OCU‐on‐Chip as a valuable tool for studying mechanotransduction in OA and provide a useful dataset for future research. Notably, we adopted primary hACs from subjects with no history of joint disease, therefore our datasets could help to pinpoint load‐dependent processes in mechanistic studies.

Nevertheless, a comprehensive analysis of all HPC‐modulated pathways is beyond the scope of this study. Therefore, given that i) hACs from different clusters demonstrated an extremely limited number of common DEGs upon HPC (Figure S24B, Supporting Information), ii) cluster 5 better retained the transcriptional variability of native hACs (Figure 7), and iii) OCU‐on‐Chip co‐cultures express higher levels of early OA gene markers (Figure 8D) we focused only on hACs derived from co‐cultures and belonging to cluster 5, in order to investigate mechanisms possibly relating to OA mechanical initiation.

Plotting the protein‐protein interaction network constructed from HCP‐modulated genes in cluster 5 revealed the presence of gene bundles associated with specific biological processes (i.e. Extracellular Matrix Organization, Cotranslational Proteins Targeting to Membrane, Type I Interferon Signaling, and Mitotic Cell Cycle) and connected to interaction hubs corresponding to known mechanotransduction modulators (Figure 8E,F). These included *FOS*, which was previously associated with mechanotransduction in chondrocytes,^[^ ^51^ ^]^ and *AKT1*, which was associated with cartilage calcification during endochondral ossification,^[^ ^88^ ^]^ joint specification in embryonal development,^[^ ^89^ ^]^ and cartilage pathological calcification.^[^ ^36^ ^]^ The upregulation of *AKT1* aligns with the cartilage calcification processes that we observed in the OCU‐on‐Chip upon compartment‐specific HPC.

Of note, analyzing GO terms for BPs modulated by HPC (Data S3, Supporting Information) revealed a remarkable overlap with the top 20 overexpressed pathways enriched by effectors genes (i.e. genes that are very likely to be causal for OA development) as highlighted in a recent genome‐wide association study meta‐analyses performed on 1,962,069 individuals.^[^ ^90^ ^]^ These included Connective Tissue Development, Skeletal System Morphogenesis, Cartilage Development, Cellular Response To Transforming Growth Factor Beta Stimulus, Osteoblast Differentiation, BMP Signaling Pathway, Limb Development, Limb Morphogenesis, Biomineral Tissue Development, but also pathways related to Glial Cell Proliferation and Response To Retinoic Acid. This correspondence further validates the relevance of our model in studying pathways related to OA progression.

Remarkably, we observed that numerous genes coding for ribosomal proteins (e.g., *RPS29*, *RPS7*, *RPL26*, *RPL37*) were affected by HPC (Figure 8E, ribosomal‐related proteins are colored in red). Alterations in ribosome biogenesis, ribosomal RNA transcription, and preferential protein translation^[^ ^37^ ^]^ have been previously detected in clinical samples (e.g. *RPS29* is expressed at higher levels in OA patients.^[^ ^72^ ^]^) but – to the best of our knowledge – they were never before reported using in vitro models, nor were they specifically connected to mechanotransduction signaling. Our data demonstrate that the OCU‐on‐Chip can recapitulate these phenomena and suggest a formerly unestablished direct connection between mechanical overloading and ribosomal alterations.

The majority of modulated genes coding for ribosomal proteins were connected to nucleophosmin (*NPM1*) (Figure 8E), which is implicated in processes such as mitotic spindle assembly, chromatin remodeling, DNA repair, embryogenesis, and ribosome synthesis.^[^ ^91^, ^92^ ^]^ Moreover, cell cycle, interferon‐related signaling, and ribosomal protein gene bundles were all linked to *MDM2*, a ligase that mediates the ubiquitination of p53 and was associated with rheumatoid arthritis.^[^ ^93^ ^]^ *MDM2* downregulation (as detected in our samples) can lead to increased apoptosis, and the protein is known to interact with the ribosome biogenesis‐related *NPM1*.^[^ ^91^, ^92^ ^]^ A RP‐Mdm2‐p53 axis activated by the RPL37 protein (significantly upregulated by HPC in our dataset) was linked to cell cycle arrest in cancer.^[^ ^94^ ^]^

Ulterior mechanistic studies will be necessary to properly elucidate the cause‐effect relationship of these pathways. However, these findings suggest that similar processes might be activated by HPC in cartilage, and validate our OCU‐on‐Chip as a suitable model to highlight new druggable pathways.

Collectively, these data introduce new fundamental knowledge of hACs’ responses to aberrant mechanical stimuli and indicate that transcriptional alterations following HPC, and supposedly OA, go far beyond typically considered metabolic^[^ ^95^ ^]^ and inflammatory^[^ ^96^, ^97^ ^]^ pathways. OA is classically regarded as the result of an imbalance between anabolic and catabolic processes.^[^ ^95^ ^]^ A recent view of the pathology fine‐tunes the anabolism/catabolism paradigm characterizing OA as an acquired ribosomopathy.^[^ ^37^ ^]^ Our data corroborate this interpretation and establish a link between ribosome alterations and aberrant mechanical stimuli.

## 3 Conclusion and Limitation

In this work, we introduced a microscale platform that allows to easily and reliably obtain 3D biphasic microtissues constituted by two directly interfaced, superimposed layers, and to subject them to tissue‐specific hyperphysiological confined compression. Through this approach, we enable the study of mechanical risk factors and cartilage‐osteochondral interface interactions, two interplaying key components of OA pathophysiology. Our data highlight distinct cross‐talks between hACs‐based cartilaginous constructs and bmMSCs‐based subchondral constructs and indicate that, upon hyperphysiological compression, the OCU‐on‐Chip mirrors clinically observed alterations in ribosome biogenesis, cell cycle, and apoptosis, beside chondrocalcinosis and hypermineralization of the osteochondral interface. We also provide comprehensive datasets for the investigation of mechanotransduction in hACs.

From a technological point of view, the newly proposed VBV geometry allows to achieve superimposed 3D constructs (i.e. structures that mimic the OCU functional anatomy) by the simple sequential injection of two hydrogels. The increased control over physical forces which is reachable at the microscale was previously exploited for different purposes. For instance, centrifugal forces were employed for the robust generation of multiple cardiac micro constructs.^[^ ^98^ ^]^ Here, we made use of the prevalence of surface forces (i.e. surface tension) over volumetric ones (i.e. gravity) to translate the CBV concept to a vertical configuration.

The VBV can be coupled with a pneumatic compartment, thus allowing the application of physical stimuli. Previous approaches to achieve vertically stacked microscale tissues required the use of coatings and sacrificial molds,^[^ ^43^ ^]^ which greatly limit the experimental throughput^[^ ^98^ ^]^ and impede the integration of pressure‐dependent components. Additionally, our multi‐layer fabrication methodology permits to customize the features of top and bottom compartments. This makes our platform versatile and suitable for applications beyond the osteochondral interface. Importantly, our OCU‐on‐Chip is strain‐controlled, as we validated with both computational and experimental approaches, meaning that the mechanical properties of cartilage and subchondral bone do not need to exactly match those of in vivo counterparts for the tissues to be exposed to physiologically meaningful deformations. These are determined by the device's geometry. While different biomaterials can mimic osteochondral‐like architectures and mechanical properties, our solution allows the application of compression levels like those of cartilage and bone to a wide range of materials and cellular constructs, irrespective of their mechanical properties and with a high control over the experimental variables. This feature is showcased by our assessment of how cartilage constructs’ response to loading is influenced by the composition of the subchondral layer. By using plastic substrates we could decouple the effects of mechanics from those of inter‐tissues cross‐talks.

From a biological point of view, we introduced a methodology to obtain microscale biphasic osteochondral constructs constituted by hACs (the cartilage layer) and bmMSCs (the mineralized osteochondral interface) possessing distinct transcriptomic profiles that correlate with embryonal development, when hyaline and transient cartilage are selectively exposed to Wnt and BMP signaling.^[^ ^99^ ^]^ At the macroscale, a number of strategies have been introduced to obtain osteochondral constructs, including bi‐phasic scaffolding, bioreactor technologies, and growth factor/gene delivery,^[^ ^61^ ^]^ but these solutions are often difficult to translate to OoC devices. Other authors introduced microscale osteochondral models based on the selective differentiation of bmMSCs in cartilaginous and osseous tissues.^[^ ^15^, ^18^, ^19^ ^]^ However, the investigation of cartilage‐subchondral tissues cross‐talk requires to use of different cell types. As we assessed with clinical samples, cartilage surface and osteochondral interface have a gene expression that varies up to two orders of magnitude in terms of cartilage markers, hypertrophy markers, ossification markers, and genes related to Wnt and BMP pathways. We obtained bi‐phasic tissues using a developmentally inspired endochondral ossification process, where a cartilage structure prefigures the presence of long bones, and we avoided the incorporation of exogenous mineral components (e.g. hydroxyapatite) to better assess de‐novo deposition of mineralized matrix.

We also demonstrated the presence of various cross‐talks: on one end the co‐culture leads to cartilaginous constructs with a higher expression of chondrogenic markers, while on the other it promotes the appearance of hypertrophic populations found in OA. On this regard, our scRNA‐seq analyses revealed a profound effect of the subchondral layer on hACs transcriptome. Co‐culture and HPC are sufficient to preserve the heterogeneity of hACs subpopulations found in OA cartilage,^[^ ^72^ ^]^ maintain progenitor‐like cells,^[^ ^73^ ^]^ obtain a gene expression possibly indicative of early OA,^[^ ^78^ ^]^ and induce hACs hypertrophic differentiation without inflammatory cytokines.

Finally, we provided an overview of the pathways affected by compartment‐specific HPC, demonstrating a good alignment with biological processes identified performing multi‐omics single‐cell analyses in clinical settings.^[^ ^90^ ^]^ Ongoing studies are being performed to provide mechanistic insights into these pathways and link gene expression to functional outcomes.

Some potential limitations of our study need to be discussed. First, our platform is constituted by PDMS, which is prone to the absorption of small, hydrophobic molecules.^[^ ^100^ ^]^ Appropriate coatings or compensation strategies should therefore be adopted when using our OCU‐on‐Chip for drug screening studies. Moreover, PDMS devices are typically single‐use. Approaches to introduce reusable devices could be implemented in the future. Secondly, our OCU‐on‐Chip belongs to the category of multi‐tissue models for which, in general, scaling approaches are required to properly dimension the relative volumes of the different tissues.^[^ ^101^, ^102^ ^]^ In our design, we opted for a similar volume for the cartilage and mineralized constructs, to maximize cross‐talk effects at the OCU interface, but future approaches will have to consider the relative masses of the two tissues.

Thirdly, cartilage and subchondral bone in vivo are subjected to a wide variety of stimuli, including compression but also shear^[^ ^103^ ^]^ and rotation.^[^ ^23^, ^104^ ^]^ Our OCU‐on‐chip remains therefore a simplification of the joint environment. Nevertheless, we purposely designed the device to apply confined compression, which leads to transient hypo‐osmotic stresses due to interstitial fluid motion and enhances chondrocytes' mechanotransduction.^[^ ^51^ ^]^

Finally, properly modeling inter‐tissue crosstalk in OA would imply the incorporation of other joint cells and compartments. Synovial cells are fundamental for the production of inflammatory cytokines that cause the chronic low‐grade inflammation state of OA.^[^ ^105^ ^]^ Subchondral bone remodeling processes depend on the interplay between osteoblasts and osteoclasts, among other cells,^[^ ^106^ ^]^ and vascular cells are necessary to study cartilage vascular invasion.^[^ ^107^ ^]^ Other authors introduce osteochondral OoCs with a higher cellular complexity.^[^ ^15^, ^21^ ^]^ In this work we purposefully decided to use a reductionist approach, to specifically study the crosstalk of hyaline cartilage with calcified cartilage at the osteochondral interface, which is seldom considered in in vitro models. Of note, our approach is compatible with methodologies proposed by other authors for the generation of micro‐physiological models of vascularized bone.^[^ ^108^ ^]^ For instance, OoC systems to investigate the role of vascular invasion in bone development and regeneration^[^ ^109^ ^]^ could be integrated with our OCU‐on‐Chip, in order to assess the effect of mechanical overloading on these processes. Given the complexity of the interplay among these phenomena, however, we believe that the individual investigation of single parameters is necessary before moving to systems with yet a higher degree of complexity.

Collectively, our findings indicate that the OCU‐on‐Chip represents a valuable tool for future investigations on the molecular drivers of OA's mechanical phenotype and on strategies to counteract aberrant calcification processes. Moreover, our data can be further mined to identify new druggable targets for disease‐modifying OA therapy.

## 4 Experimental Section

### Devices’ Design and Fabrication

Microscale systems were realized in PDMS (Sylgard 184; Dow Corning) polymerized with defined casts at a 10:1 weight ratio of base to curing agent. All PDMS parts were cured for at least 2 h at 65 °C. Final devices were produced by bonding four different layers (Figure 2C; Figure S4, Supporting Information). Unpatterned actuation membranes were achieved by pouring defined quantities of PDMS into a Petry dish (Sigma Aldrich; 100 mm x 15 mm polystyrene Petri dishes) containing a clean silicon wafer. The PDMS volume was optimized to obtain a final thickness of 800 µm. Polymerization was achieved on a leveled surface. All other layers were obtained by replica molding of appropriately designed master molds. Layers' schemes were realized through Computer Aided Design (CAD) Software (AutoCad 2022, Autodesk). Master molds were fabricated in a cleanroom environment (Class ISO 6) using multilayer direct laser writing (Heidelberg MLA100) of SU‐8 2035 or SU‐8 2100 (MicroChem) onto 102 mm silicon wafer substrates. A detailed description of design and fabrication procedures is available in Supplementary Methods.

### Devices’ Geometrical Characterization

Devices’ geometrical features were measured through observation of their cross sections. Thin PDMS slices (1–2 mm thick, *n* > 6) were cut with a razor blade and observed through a brightfield microscope connected to a digital camera (AmScope MU500); features’ dimensions were measured from acquired images. Image analysis was performed using Image J. Assembled PDMS layers of top and bottom compartments were also imaged through SEM (LEO 1525 Field Emission Scanning Electron Microscope). PDMS devices were coat‐sputtered with a few nanometers of gold. Images were acquired adopting an Electron High Voltage (EHV) of 10.00 kV.

### PEG Precursor Production and PEG Hydrogel Preparation

PEG hydrogels were produced as previously described.^[^ ^44^ ^]^ 1 ml of FXIII (200 U mL^−1^; Fibrogammin; CSL Behring) was activated with 100 µL of thrombin (20 U mL^−1^; Sigma–Aldrich) for 30 min at 37 °C and the resulting activated FXIII (FXIIIa) was stored in small aliquots at −80 °C. Eight‐arm PEG vinylsulfone (molecular weight: 40 kDa; NOF Europe) was functionalized with peptides that contained either an FXIII glutamine acceptor substrate (Gln peptides; NQEQVSPL‐ERCG‐NH2; Bachem) or an MMP‐degradable FXIII lysine donor substrate (Lys‐MMPsensitive peptides; Ac‐FKGGGPQGIWGQ‐ERCG‐NH2; Bachem), resulting in 8‐PEG‐Gln or 8‐PEG‐MMPsensitive‐Lys precursors, respectively. A stoichiometrically balanced solution of 8‐PEG‐Gln and 8‐PEG‐MMP sensitive‐Lys was mixed in Tris buffer (50 mm Tris (pH 7.6)) containing 50 m calcium chloride for the indicated final dry mass content of hydrogel precursors, leaving a spare volume of 10% v/v for the addition of culture medium and cells. Hydrogel cross‐linking was initiated by adding 10 U mL^−1^ FXIIIa and vigorous mixing. Adopted Hydrogels had a final concentration of 2% PEG precursors and contained cells at a 50 × 10^6^ cells mL^−1^ density.

### Finite Element Analyses

Abaqus 6.14 (Abaqus FEA: Dassault systems), established to be adequate for the biomechanical description of biphasic tissues,^[^ ^47^ ^]^ was adopted to perform computations. A biphasic poroelastic constitutive behavior was considered for PEG‐based hydrogels and cartilaginous samples. PDMS was described as a hyperelastic solid with a Mooney‐Rivlin strain energy function.^[^ ^14^, ^110^ ^]^ Details about the simulations’ specifics, geometry, and boundary conditions are available in Supplementary Methods.

### Indentation Type Atomic Force Microscopy (IT‐AFM) Measurements

AFM indentation was adopted to determine the local E Modulus of i) cartilaginous constructs, ii) the PEG‐based hydrogel adopted to encapsulate chondrocytes, iii) the beads‐laden PEG hydrogels used to assess the effect of substrates’ mechanical properties on cartilaginous constructs’ response to loading, iv) mature OCU‐on‐Chip constructs and v) osteochondral biopsies from OA clinical samples. OoCs devices were glued onto a plastic culture dish (Sigma‐Aldrich, 30 mm diameter) with the exposed cellular constructs facing upward. Two components epoxy resin (Araldite Rapid, Huntsman corporation) mixed with a black dye to increase image contrast was adopted for sample fixation. Measurements were performed at room temperature in degassed PBS. OCU‐on‐Chip constructs’ measurements were performed by selecting, respectively, cartilaginous areas and dark mineralized areas in hACs’ and bmMSCs ‘compartments. Osteochondral samples were obtained from donors undergoing total knee replacement. Biopsies were taken from the femoral condyles through an 8G bone marrow needle (Argon medical devices). Biopsies were then cut in half with a razor blade and IT‐AFM was performed on the flat side of the half‐cylinders.

Measurements on cartilaginous constructs, empty PEG hydrogels, and osteochondral biopsies were conducted using the ARTIDIS ADO (Automated Device Operation) AFM (ARTIDIS AG, Basel, Switzerland). Indentations were performed using a silicon nitride cantilever (DNP‐S10 D, Bruker AFM Probes, Santa Barbara, USA) with a nominal stiffness of 0.06 N m^−1^ and a pyramidal tip of ≈6 µm of height and a typical radius of the uncoated tip of 10 nm. The exact spring constant of each adopted cantilever was determined through the thermal tune method.^[^ ^111^ ^]^ The deflection sensitivity was determined directly on the dish, as previously reported.^[^ ^64^ ^]^ Three constructs were measured for each experimental condition. For each construct, at least 5 different force maps were acquired at random sample locations. Force maps with dimensions of 20 µm × 20 µm were acquired with a submicron resolution, each force map containing 20 × 20 force curves (400 force–displacement curves per map). Each force curve was recorded at an indentation speed of 16 µm s^−1^. Force displacement curves were corrected for tilt and tip‐sample displacement as previously documented.^[^ ^64^ ^]^ Both Forward and Backward force–displacement (*F–D*) curves were recorded. Backward curves were adopted to extract the average Young's modulus of samples making use of the Oliver‐Pharr model, as suitable for hydrogels and soft materials.^[^ ^112^ ^]^ An elastic modulus histogram was derived for each experimental group and mean and standard deviation were calculated. Further details on IT‐AFM measurements of osteochondral biopsies are described in Supplementary Note S3 (Supporting Information).

Measurements on OCU‐on‐Chip constructs were performed through the JPK NanoWizard 4 AFM (Bruker Nano GmbH, Berlin, Germany) with the same above‐described specifications.

Measurements on beads‐laden PEG hydrogels were performed using the JPK NanoWizard 4 AFM (Bruker Nano GmbH, Berlin, Germany) as described below. Indentations were performed with Sharp Silicon Nitride tips (radii of around 10 nm, tip height of 2.5–8 µm) mounted on soft triangular‐shaped Silicon Nitride cantilevers (MSCT‐A, Bruker AFM Probes, Camarillo, USA) with nominal spring constants of 0.07 N m^−1^. Actual spring constants were determined prior to every experiment by using the Sader method. Force spectroscopy was performed with a load of 800 – 1000 pN, with indentation depths ranging between 500 and 1000 nm. Loading and unloading speeds were set to 10 µm s^−1^. Three samples for each condition were probed recording *F–D* curves at two different random locations over a scanning area of 40 × 40 µm (32 × 32 force curves) for each sample. E values were calculated from backward *F–D* curves with the JPK Data Processing software using the quadratic pyramid model. An elastic modulus histogram was derived for each experimental group.

### Human Cartilage Collection, and Chondrocytes Isolation and Expansion

Knee cartilage specimens from a total of 8 patients (age: 56 ± 13 years, 5 males, 3 females) with no clinical history of OA and/or without evident signs of cartilage fibrillation in the harvested area were collected working under general consent of the University Hospital of Basel, after informed consent from the relatives and in accordance with the Ethical Committee of northwest and central Switzerland (EKNZ). Samples were minced and enzymatically digested to extract hACs. hACs were isolated using 10 mL of a 0.15% (w/v) type II collagenase (300 U mg^−1^; Worthington Biochemical Corporation) solution per g of tissue (37 °C, 22 h) and resuspended in Dulbecco's modified Eagle's medium (DMEM) containing, 4.5 mg mL^−1^ d‐glucose, 0.1 mm non‐essential amino acids, 1 mm sodium pyruvate, 100 mm HEPES buffer, 100 U mL^−1^ penicillin, 100 µg mL^−1^ streptomycin, 0.29 mg mL^−1^ l‐glutamine, and 10% fetal bovine serum (FBS), i.e. complete medium. Isolated hACs were counted using Trypan blue (Thermo‐Fisher), plated in culture flasks at a density of 10^4^ cells cm^−2,^ and expanded (in a humidified incubator, 37 °C, 5% CO_2_) in a complete medium supplemented with 1 ng mL^−1^ of transforming growth factor‐β1 (TGF‐β1) and 5 ng mL^−1^ of fibroblast growth factor‐2 (FGF‐2). Passage 3 hACs were used in experiments if not otherwise specified. Further cartilage samples from 3 OA patients (age: 64 ± 8 years, 3 females) undergoing total or unicondylar knee replacement were harvested from preserved areas of the dorsal part of the femoral condyles. Samples were obtained working under the general consent of the hospital after informed consent from the relatives and in accordance with the Ethical Committee of Northwest and Central Switzerland (EKNZ). Cartilage from superficial and deep zone/calcified cartilage were dissected and cells from the two areas were collected separately. Samples were digested as described above and used for RT‐qPCR analyses.

### Human bmMSCs Collection, Isolation, and Expansion

Human bmMSCs were isolated from bone marrow aspirates (20 mL volume) harvested from 5 healthy donors (age: 34 ± 13 years, 3 males, 2 females) during routine orthopedic procedures involving exposure of the iliac crest. Bone marrow harvesting was performed after informed consent and in accordance with the local ethics committee (University Hospital Basel, Switzerland). A bone‐marrow biopsy needle (Argon medical devices) was inserted through the cortical bone and the aspirate was immediately transferred into plastic tubes containing 15 000 IU heparin. Isolated bmMSCs were counted using Crystal violet, plated in tissue culture flasks, and expanded in a humidified incubator (37 °C, 5% CO_2_) in minimum essential medium eagle – Alpha modification (α‐MEM), containing 10% FBS, 4.5 mg mL^−1^ D‐glucose, 0.1 mm nonessential amino acids, 1 mm sodium pyruvate, 100 mm Hepes buffer, 100 UL mL^−1^ penicillin, 100 µg mL^−1^ streptomycin, and 0.29 mg mL^−1^ L‐glutamate, and further supplemented with 5 ng mL^−1^ of FGF‐2 (R&D Systems). The medium was changed twice a week. After ≈10 days, when they were ≈80% confluent, cells were rinsed with phosphate‐buffered saline (PBS), detached using 0.05% trypsin/0.53 mM EDTA, and replated at 5 × 10^3^ cells cm^−2^. Passage 3–4 bmMSCs were used in experiments if not otherwise specified.

### Constructs Maturation

Cartilaginous and mineralized subchondral microconstructs were obtained by embedding hACs or bmMSCs into an enzymatically cross‐linkable and degradable PEG‐based hydrogel^[^ ^44^ ^]^ with a final dry mass content of 2%. The hydrogel formulation was prepared as described above. Biphasic constructs were generated through subsequent injections of appropriate cell‐laden hydrogels in the bottom and top compartments of the OCU‐on‐Chip device. The cell‐polymer precursor solution was mixed with 10 U mL^−1^ of thrombin‐activated factor FXIIIa, immediately injected into microscale devices, and incubated for 10 min (37 °C, 5% CO_2_). The procedure was repeated for the second gel when needed.

An appropriate differentiation medium was injected into the dedicated channels. Chondrogenic medium (CHM) was constituted by DMEM (Sigma–Aldrich) containing, 4.5 mg mL^−1^ D‐glucose, 0.1 mm non‐essential amino acids, 1 mm sodium pyruvate, 100 mm HEPES buffer, 100 U mL^−1^ penicillin, 100 µg mL^−1^ streptomycin, 0.29 mg mL^−1^ l‐glutamine, 1.0 mg mL^−1^ insulin, 0.55 mg mL^−1^ human transferrin, 0.5 µg mL^−1^ sodium selenite, 50 mg mL^−1^ bovine serum albumin, 470 µg mL^−1^ linoleic acid, and 1.25% of human serum albumin, and supplemented with 0.1 mm ascorbic acid 2‐phosphate, 10^−4^ mm dexamethasone, and 10 ng mL^−1^ TGF‐β3. Osteochondral medium (OCM) was obtained by adding 10 mm beta‐Glycerophosphate (β‐Gly, Sigma Aldrich) to the CHM. The culture medium was changed every 2 days. Samples were cultured in static regimen for 14 days, previously demonstrated to be sufficient for constructs maturation,^[^ ^14^ ^]^ before application of compartment‐specific, cyclic HPC or collection for RT‐qPCR, immunofluorescence imaging, or IT‐AFM analyses. bmMSCs‐based constructs cultured in OCM were also harvested after 7 days, to assess the evolution of constructs’ gene expression over time. Brightfield images of the constructs were acquired throughout the culture period.

### Application of Mechanical Compression

After 14 days of maturation, constructs were subjected to compartment‐specific, cyclic HPC for a further 7 days. Loading was applied following a previously tested regimen demonstrated to induce OA traits on‐chip:^[^ ^14^ ^]^ two 2‐h stimulation cycles (1Hz, 50% duty cycle) with a four‐hour stop period in between. Cyclic loading was obtained by applying a pressure of 0.4 Atm to the actuation chamber, sufficient for the actuation membrane to reach the bottom surface of the pillars in the bottom compartment. Mechanical stimulation was achieved by connecting the devices to a pressure regulator (Comnhas) linked to a custom‐made electro‐pneumatic controller able to apply the described loading regimen.^[^ ^14^ ^]^ External connections were realized through Tygon tubing (internal diameter = 0.5 mm, Qosina, NY, USA). The culture medium was changed every second day and collected for analysis. Dexamethasone, which has anti‐inflammatory effects,^[^ ^14^ ^]^ was removed from the culture medium during the mechanical stimulation period. At the end of the stimulation period, constructs were collected for analyses, possibly after digestion and cell sorting.

### Cells Transduction

GFP+ hACs were obtained through lentiviral transduction adopting a protocol previously demonstrated not to affect chondrocytes’ differentiation capacity.^[^ ^113^ ^]^ First passage hACs were seeded in Petri dishes (Sigma Aldrich; 60 mm × 15 mm polystyrene Petri dishes) at a density of 10^4^ cells cm^−2^ and transduced the following day with 2.8 × 10^6^ TU of GFP lentivirus (Multiplicity of Infection, MOI, of 5) in presence of 5 µg mL^−1^ of protamine sulfate. Cells were detached 3 days post‐transduction, and GFP+ cells were selected through FACS (BD FACSAria III Cell sorter). GFP+ hACs were subsequently re‐passaged and used in passages 3–5. mCherry+ bmMSCs were obtained through lentiviral transduction as follows. For lentivirus production, Lenti‐X 293T cells (Clontech, United States) were transfected with lentiviral expression vector (pLVX_mCherry (backbone vector from Clontech)) and 3rd generation packaging plasmids prMDLg/pREE, pRSV‐Rec, and pMD2.G (Addgene, #12251, #12253, and #12259, respectively) using Lipofectamine 2000 (Lifetech, #11668019). After 72 h, the supernatant containing lentiviral particles was collected, and the lentiviral titer was assessed by ELISA using the Quick Titer Lentivirus titer kit (Cell Biolabs, #VPK‐1070). For lentiviral transduction, bmMSCs were seeded in flasks at a density of 10^4^ cells cm^−2^, and the lentivirus was added the following day with an MOI of 1 in the presence of 8 µg mL^−1^ polybrene (Sigma, #107689). Viral particles were removed after 12 h. After 48 h, transduced bmMSCs were selected using G418 (Geneticin, Roche, #4727878001) for 8 days. mCherry+ bmMSCs were subsequently re‐passaged and used in passages 4–5.

### Sorting of GFP+ Chondrocytes

To verify if constructs in the two compartments were effectively subjected to different compression levels, GFP transduced chondrocytes (i.e. GFP+ hACs) were injected in one of the compartments and non‐labeled hACs in the remaining one. Constructs were cultured statically for 2 weeks and subjected to mechanical loading for 7 days, as described above. At the end of the culture period, constructs were extracted from devices and incubated on an orbital shaker for 1 h in a solution of 0.15% type II collagenase (Worthington Biochemical Corporation) in DMEM (300 µL per construct). Cells were then centrifuged, rinsed in a solution of 1mm EDTA and 2% FBS in PBS, incubated for five minutes in 0.05% trypsin/0.53 mm EDTA (37 °C, 5% CO_2_), washed twice in 1mM EDTA‐2% FBS in PBS, and held on ice prior to sorting. GFP+ and GFP‐ populations were sorted (BD FACSAria III Cell sorter) and analyzed separately. To assess the efficiency of the process, RT‐qPCR for *EGFP* was performed on sorted populations and on single GFP+ and GFP‐starting populations, as controls. Sorted populations were also replated in 2D (IBIDI μ‐Slide 8 well) and left to adhere for 48 h in a complete medium (using nonsorted population as controls) to directly visualize the population purity following FACS. After 48 h, samples were rinsed with PBS and fixed with a solution of 4% formalin for 24 h. Population purity after sorting was confirmed through immunofluorescent imaging. Similar procedures were performed seeding OCU‐on‐Chip devices with bmMSCs in the top compartment and GFP+ hACs in the bottom compartment.

### Determination of the Effect of Subchondral Layer's Mechanical Properties on hACs’ Response to HPC

The effect of introducing local alterations in the mechanical properties of the subchondral layer during compression was investigated as follows. hACs were loaded in an enzymatically cross‐linkable and degradable PEG‐based hydrogel formulation^[^ ^44^ ^]^ and injected in the bottom compartment of the device. The top compartment was injected with hydrogels loaded with Polystyrene beads (diameter 10 um, Sigma‐Aldrich) with volumetric fractions of either 0.1% or 5%. Hydrogel mechanical properties were assessed by IT‐AFM. Constructs were cultured statically for 14 days in CHM, before application of the above‐described loading regimen for 7 further days. During stimulation, the culture medium (changed every other day) was collected for biochemical analysis. Constructs were harvested for RT‐qPCR analyses.

### Gene Expression Analysis

Total RNA extraction by TRIzol (Sigma), complementary DNA synthesis, and RT‐qPCR were performed according to standard protocols (7300 AB; Applied Biosystems). The following gene of interests were quantified (Applied biosystems): *FOS* (Hs99999140_m1), *PTGS2* (Hs00153133_m1), *C‐JUN* (Hs01103582_s1), *MMP13* (Hs00233992_m1), *CXCL8* (Hs00174103_m1), *COL2A1* (Hs00264051_m1), *COL1A1* (Hs00164004_m1), *ACAN* (Hs00153936_m1), *PRG4* (Hs00981633_m1), *FRZB* (Hs00173503_m1), *DKK1* (Hs00183740_m1), *GREM1* (Hs01879841_s1), *GDF5* (Hs00167060_m1), *EGFP* (Mr00660654_cn), *COL10A1* (Hs00166657_m1), *IHH* (Hs01081800_m1), *BMP2* (Hs00154192_m1), *BSP2* (Hs00173720_m1), *ALP* (Hs01029144_m1), *OCN* (Hs01587814_g1), *OPN* (Hs00959010_m1), *SOX9* (Hs00165814_m1), *COMP* (Hs01561086_g1), *COL3A1* (Hs00943809_m1), and *THY1* (Hs00264235_s1). *GAPDH* (Hs02758991_g1) was used as housekeeping gene.

### Biochemical Analyses

Supernatants were collected on day 16, 18, 20, and 21, during the mechanical stimulation phase, and pooled together to measure solute accumulation during the whole stimulation period. Culture medium from the top and bottom compartments was collected and analyzed separately to ascribe the release of solutes to only one of the two compartments. IL8 concentration was measured using the Human IL‐8 ELISA Set (BD Bioscience). Measurements were acquired using a configurable multimode microplate reader (synergy H1, BioTek instruments). The concentration of IL6 was analyzed by Luminex® Assay using the Human Premixed Multi‐Analyte Kit (R&D Systems) and acquiring readings with a BioPlex MagPlex Magneti Beads system (Bio‐Rad). (pro)MMP‐13 production was measured using an enzyme‐specific fluorescence substrate kit (SensoLyte 520 MMP‐13 Assay Kit; AnaSpec). All measurements were performed according to manufacturers’ instructions.

### Immunofluorescence Analyses

Immunofluorescence analyses were performed on cellular constructs at day 0, day 14, and day 21, directly within microscale devices. Samples were washed with PBS and fixed in 4% paraformaldehyde overnight at 4 °C. Devices were subsequently disassembled removing the layers corresponding to the actuation membrane and actuation chamber to expose the constructs (for top view images) or cut in sections with a thickness of roughly 1 mm (for side view images). Cells were permeabilized with a solution of 0.5% (v/v) of Triton X (Sigma‐Aldrich); unspecific binding was blocked (1 h at room temperature) with a solution of 0.3% (v/v) Tween 20 and 3% (v/V) Goat serum in PBS. Samples were then incubated overnight at 4 °C with primary antibodies. Aggrecan (ACAN, Abcam, ab36861, dilution 1:200), collagen type II (COL2A1, Abcam, ab185430, dilution 1:200), osteocalcin (OCN, Millipore, ab1857, dilution 1:200), and alkaline phosphatase (ALP, Abcam, ab54778, dilution 1:200) were used to assess tissues’ maturation. Stainings for cluster 5 markers were performed with antibodies for phosphoglycerate dehydrogenase (PHGDH, Abcam, ab236763, dilution 1:200), Myocyte Enhancer Factor 2C (MEF2C, Invitrogen, MA5‐25477, dilution 1:200), Ectonucleotide pyrophosphatase/phosphodiesterase family member 1 (ENPP1, Abcam, ab223268, dilution 1:200), and sulfatase 1 (SULF1, Invitrogen, PA5‐115984, Dilution 1:200). After washing with blocking solution (20 min at room temperature, repeated twice), samples were incubated (1 h at room temperature) with antibodies labeled with Alexa Fluor 488, Alexa Fluor 546, and Alexa Fluor 647 (Invitrogen, dilution 1:200) as appropriate, and subsequently washed with PBS. Staining with 4′,6‐diamidino‐2‐phenylindole (DAPI) was used to identify the cell nuclei. Deposition of hydroxyapatite (HA) was assessed by staining samples with OsteoImage mineralization assay (Lonza) according to the manufacturer's instructions.

Stainings on patients’ tissues were performed as follows. Osteochondral biopsies were obtained as described above, fixed in 4% formalin (Formafix) at 4 °C for 24 h, decalcified in a 15% (w/v) Ethylenediaminetetraacetic acid (EDTA) solution, and embedded in paraffin. Biopsies were then cut into 5 µm thick sections using a Microm HM 355S (Thermo Scientific) and the sections were placed onto Poly‐lysine slides (Thermo Scientific, Waltham, US). Tissues’ sections were de‐paraffinized and re‐hydrated before stainings. Antigen retrieval was performed by incubating samples with hyaluronidase (Sigma, H3884, 2 mg mL^−1^, 1 h at 37 °C) and pronase (Roche, 10 165 921001, 1 mg mL^−1^, 30 min at 37 °C). Stainings were subsequently performed as described above.

Imaging was performed using the following microscopes: Nikon Eclipse Ti 2 widefield microscope, Nikon A1R NALA confocal microscope, Nikon AxR confocal microscope, and Nikon Crest V3 spinning disc microscope. GFP+ and mCherry+ cells were imaged directly without prior stainings. Image analysis was performed using Image J and QuPath software.

### hACs and bmMSCs Culture in 3D Aggregates (i.e. Pellets)

hACs and bmMSCs isolated and expanded as described above were cultured in 3D pellets for 14 days in either CHM or OCM. 2.5 × 10^5^ cells were adopted for each pellet. Cellular aggregation was obtained via centrifugation (1300 RPM, 3 min), in screw top microtubes (Thermo‐fisher). The culture medium, 1 ml for each pellet, was changed twice a week.

### GAG and DNA Quantifications

Samples were digested overnight at 56 °C in 500 µL of proteinase‐K solution (1 mg mL^−1^ proteinase‐K in 50 mm Tris with 1 mm EDTA, 1 mm iodoacetamide, and 10 µg mL^−1^ pepstatin‐A). GAG amounts were measured spectrophotometrically after reaction with dimethylmethylene blue using chondroitin sulfate as a standard.^[^ ^114^ ^]^ DNA was quantified using the CyQuant cell proliferation assay Kit (Molecular Probes, Eugene, OR) according to the manufacturer's instructions.

### Histological Staining and Immunohistochemistry

Cell pellets were washed with PBS, fixed in 4% paraformaldehyde overnight at 4 °C, and embedded in paraffin. After deparaffinization and re‐hydration, sections were stained for Safranin‐O (Fluka) and Alizarin red (Sigma) to assess GAG and calcium depositions respectively.

Immunohistochemical staining, for COL2A1 (MP Biomedicals 63171, 1:1000 dilution) and COL10A1 (Invitrogen 14‐9771‐80, 1:200 dilution) was performed with Ventana Discovery Ultra (Roche Diagnostics (Switzerland), SA) automated slide stainer. Tissue sections were deparaffinized and rehydrated. Antigens were retrieved by protease digestion (Protease 3, 760–2020, Ventana) for 20 to 44 min at 37 °C. Primary antibodies were manually applied and incubated for 1 h at 37 °C. After washing, the secondary antibody (anti‐mouse polymer horseradish peroxidase (HRP), R&D Mouse IgG (VC001‐025, VisUCyte)) was incubated for 1 h at 37 °C. The detection step was performed with the Ventana DISCOVERY ChromoMap DAB (760‐159, Ventana) detection kit. Slides were counterstained with hematoxylin II, followed by the bluing reagent (respectively, 790–2208 and 760–2037; Ventana). Histological and immunohistochemical sections were imaged using a Nikon Eclipse Ti2 microscope with a Nikon DS‐Ri2 camera. Safranin‐O images were adopted for tissue histological grading through the Modified Bern Score.^[^ ^115^, ^116^ ^]^ Grading was performed as previously described,^[^ ^116^ ^]^ through a deep learning‐based automated procedure using tiles of 224 × 224 pixels with a pixel dimension of 0.511 µm.

### Mineral Deposition Quantification

The effect of cyclical loading on osteochondral tissue mineralization was analyzed by quantifying the amount of hydroxyapatite (HA) deposited in OCU‐on‐Chip constructs, as well as the calcium salts deposited in the culture medium channels and reservoirs of both OCU‐on‐Chip and single‐culture control devices.

HA deposition was detected by staining constructs sections (*n* ≥ 20 from *n* ≥ 3 chambers considering three different hACs and three different bmMSCs donors) with OsteoImage (Lonza) as detailed above. DAPI was used to counterstain nuclei. Images were quantified using QuPath. Briefly, areas corresponding to the subchondral compartment (comprehensive of top compartment and VBV necking area) and to the cartilaginous compartment were traced manually on each image. The total number of cells in each compartment was computed using the QuPath cell detection tool. The total HA‐positive area for each of the tissues was computed through a threshold‐based pixel classifier. Subchondral compartment and cartilage compartment were analyzed separately. For each compartment, the HA‐positive area was normalized for the number of cells detected in the area. The amount of calcium salts deposited in culture medium channels and reservoirs of microscale devices was quantified through an Alizarin Red staining. Devices were opened peeling of actuation membrane and actuation chamber layers, and cell constructs were removed. Empty devices were stained with Alizarin Red, completely immersing the chambers in the solution. Stained calcium salts were then dissolved in a 10% (v/v) acetic acid solution (30 min at room temperature with shaking at 300 rpm) which was then collected, incubated at 85 °C for 10 min, put immediately on ice, and centrifuged at 12 000 g for 15 min. Supernatants were analyzed quantifying the optical density at 405 nm in a Synergy H1 Hybrid Multi‐Mode Reader (BioTek Instruments, Winooski, VT, USA).

### Statistical Analysis

Results of FE analyses and biochemical analyses were presented as mean ± standard deviation. RT‐qPCR results were presented as mean + standard deviation. Single data were plotted to account for non‐normal distributions. Population normality was verified using Shapiro‐Wilk and Kolmogorov‐Smirnov tests. Non‐paired double comparisons were performed using two tailed *t*‐test for normal populations and Mann‐Whitney test for non‐Gaussian ones. Paired double comparisons were performed using paired t‐test and Wilcoxon test respectively. Multiple comparisons were performed using ordinary one‐way analysis of variance (ANOVA). Ordinary one‐way ANOVA with Tukey's multiple comparison tests for normal distributions, Kruskal‐Wallis test with Dunn's multiple comparison tests for non‐normal distributions.

### Single‐Cell RNA Sequencing (scRNA‐seq)

scRNA‐seq was performed on hACs after 21 days of culture (i.e. 14 days of static maturation and 7 days of mechanical stimulation, with constructs cultured statically for 21 days used as controls). Single culture hACs and OCU‐on‐Chip constructs were retrieved from microfluidic devices and enzymatically digested on a shaker for 30–45 min as described above. Cells were then transferred in a solution of 10% FBS in PBS and live‐dead sorted using DAPI. Enzymatic digestion time and sorting strategies were optimized to preferentially select hACs over bmMSCs. Moreover, residual bmMSCs present in the single‐cell populations were excluded from analyses based on single nucleotide polymorphism (SNP) analysis, which allowed us to identify and exclude cells originating from bmMSC donors (distinct from hACs donors). See Supplementary Methods for full details of the selection procedure. For each donor and for each condition, cells from 4 cellular constructs were pooled together. A total of 4 donors was considered. Biological replicates originating from different donors and corresponding to the same condition were then pulled together into one sample. Single‐cell capture, and cDNA and library preparation were performed at the Genomics Facility of the University of Basel.

Cells from each sample were loaded on a Chromium Single Cell Controller (10x Genomics, Pleasanton, CA, USA). Single‐cell capture and cDNA and library preparation were performed according to the manufacturer's instructions, using a Chromium Next GEM Single‐Cell 3’ Reagent Kit v3.1 (Dual Index, 10x Genomics, User Guide CG000390, Rev B) with Feature Barcode technology for Cell Surface Protein and Cell Multiplexing. Pair‐end sequencing (with read setup 28‐10‐10‐90) was performed on 1 Lane of a S4 Flowcell with an Illumina NovaSeq 6000, according to 10x specifications. scRNA‐seq data were analyzed using STARsolo (v2.7.10a) and R (v4.2.0), as described in the Supplementary Methods.

### Tissue Samples from Human Subjects

Macroscopically intact human articular cartilage was obtained from the knees of donors with no known clinical history of joint disorders. These donors included cadavers and patients undergoing surgery for traumatic joint lesions, but without a diagnosis of osteoarthritis or signs of cartilage fibrillation. Bone marrow‐derived mesenchymal stromal cells were isolated from bone marrow aspirates harvested from healthy donors during routine orthopedic procedures involving the exposure of the iliac crest. Osteochondral biopsies were collected from leftover surgical material from patients undergoing total or unicondylar knee replacement.

Cartilage samples, bone marrow samples, and osteochondral biopsies were obtained as described in appropriate sections, under the general hospital consent (University Hospital of Basel, Switzerland), following informed consent from patients or relatives, and in accordance with the Ethical Committee of Northwest and Central Switzerland (EKNZ). When applicable, samples were collected under ethical approval EKNZ 2014–199.

## Conflict of Interest

Marco Rasponi and Paola Occhetta hold equities in BiomimX. Marko Loparic and Philipp Oertle hold equities in ARTIDIS. All other authors declare they have no competing interests.

## Author Contributions

A.BA. and M.RA. contributed equally to this work. A.MA. conceived the VBV design. A.MA. and M.RA. conceived the device. A.MA. realized and functionally validated the device. A.MA. planned and performed biological experiments and analyses. A.MA. conceived and performed FE simulations. A.MA., M.LO., and P.OE. performed IT‐AFM measurements. A.MA., P.OE., and M.LO. performed IT‐AFM analyses. M.EH. and L.KR. fabricated and provided the hydrogel. A.MA., A.BA., P.OC., and M.RA. conceived the project. A.MA., P.OC., I.MA., M.RA., and A.BA. wrote the manuscript. A.MA. realized the manuscript figures. R.IV. and A.BO. performed scRNA‐seq analyses. A.MA. and A.BO. discussed scRNA‐seq data. All authors discussed the results, commented on the manuscript, and contributed to its final version.

The gene expression profiles of the samples have been deposited in NCBI's Gene Expression Omnibus^[^ ^117^ ^]^ and are accessible through the GEO Series accession number GSE237786. Additional information concerning scRNA‐seq analyses can be found in Supplementary Data S1–S3.

## Supporting information



Supporting Information

Supplemental Data 1

Supplemental Data 2

Supplemental Data 3

## Data Availability

The authors declare that all data supporting the findings of this study are available within the paper and its Supporting Information, further specifications are available from the corresponding authors upon reasonable request.
