# Supplementary material for: An Organ‐on‐Chip Platform for Strain‐Controlled, Tissue‐Specific Compression of Cartilage and Mineralized Osteochondral Interface to Study Mechanical Overloading in Osteoarthritis
Source: Adv Healthc Mater. 2025 Jun 25;14(23):2501588. doi: 10.1002/adhm.202501588 (PMC12417778; doi:10.1002/adhm.202501588)
Supplement: Supplementary file 1 — Supporting Information [file ADHM-14-0-s001.docx]

Supporting Information

An Organ-on-Chip Platform for Strain-Controlled, Tissue-Specific Compression of Cartilage and Mineralized Osteochondral Interface to Study Mechanical Overloading in Osteoarthritis

*Andrea Mainardi*, Anastasiya Börsch, Paola Occhetta, Robert Ivanek, Martin Ehrbar, Lisa Krattiger, Philipp Oertle, Marko Loparic, Ivan Martin, Marco Rasponi*, Andrea Barbero*

*Corresponding authors

E-mail: [andrea.mainardi@unibas.ch](mailto:andrea.mainardi@unibas.ch), [marco.rasponi@polimi.it](mailto:marco.rasponi@polimi.it)

Table of Contents

[SUPPLEMENTARY METHODS 4](#_Toc201502673)

[Design criteria and fabrication procedure 4](#_Toc201502674)

[Finite Element (FE) computational modelling. 5](#_Toc201502675)

[Single-cell data analysis 7](#_Toc201502676)

[SUPPLEMENTARY FIGURES 9](#_Toc201502677)

[Figure S1. Gene expression of superficial and deep zone/calcified cartilage from human knees. 9](#_Toc201502678)

[Figure S2. Device geometry. 10](#_Toc201502679)

[Figure S3. Classic CBV and VBV working principle. 10](#_Toc201502680)

[Figure S4. Device fabrication protocol*.* 11](#_Toc201502681)

[Supplementary note 1: Compartments’ alignment optimization 12](#_Toc201502682)

[Figure S5. Top compartment’s alignment: precision assessment and optimization. 12](#_Toc201502683)

[Supplementary note 2: Determination of devices’ actuation pressure 13](#_Toc201502684)

[Figure S6. Determination of the actuation pressure. 13](#_Toc201502685)

[Figure S7. Characterization of diffusion kinetics between top and bottom compartments. 14](#_Toc201502686)

[Figure S8. FE model setup. 15](#_Toc201502687)

[Figure S9. Establishment of bi-layer mature human cartilaginous constructs on-chip. 15](#_Toc201502688)

[Figure S10. hACs from top and bottom compartments can be separated based on GFP expression. 16](#_Toc201502689)

[Figure S11. Gene expression baseline of cartilaginous tissue in top and bottom compartments. 17](#_Toc201502690)

[Supplementary note 3: Osteochondral biopsies IT-AFM measurements 17](#_Toc201502691)

[Figure S12. Mechanical properties of beads laden hydrogels and OA osteochondral biopsies. 18](#_Toc201502692)

[Figure S13. OCM’s effects on hACs and bmMSCs. 19](#_Toc201502693)

[Figure S14. Co-culture effects on hACs’ maturation. 20](#_Toc201502694)

[Figure S15. Effect of compartment-specific HPC on OCU-on-Chip’s ECM deposition. 21](#_Toc201502695)

[Figure S16. Effect of HPC on hACs’, bmMSCs’, and OCU-on-Chip constructs’ gene expression. 22](#_Toc201502696)

[Figure S17. Mapping statistics and Principal Component Analysis (PCA). 23](#_Toc201502697)

[Figure S18. Characterization of clusters’ marker genes. 24](#_Toc201502698)

[Figure S19. Feature plots of classic chondrogenic markers. 25](#_Toc201502699)

[Figure S20. Expression of marker genes of OA and healthy chondrocyte subpopulations across clusters. 26](#_Toc201502700)

[Figure S21. Characterization of CPCs. 27](#_Toc201502701)

[Figure S23. KEGG pathways gene sets enrichment analyses from *in silico* bulk samples. 28](#_Toc201502702)

[Figure S24. Differentially expressed genes (static vs HPC dynamic samples) across clusters. 29](#_Toc201502703)

[References 29](#_Toc201502704)

# **SUPPLEMENTARY METHODS**

# Design criteria and fabrication procedure

The structure of OCU-on-Chip devices was conceived to provide stacked bi-layer constructs with compartment-specific confined compression. Schematics of the compartments’ inner structures are reported in Figure S2. Top compartments’ features had a nominal height of 150 µm and were realized by the sequential realization of the narrow canal layer (narrow canals: 10 µm width, 50 µm height, 50 µm separation between them), and the central channel layer (height 100 µm, width 300 µm). Three versions, with a central channel width of, respectively, 300 µm, 400 µm, and 500 µm were realized. After assessing the precision of aligning top and bottom compartments, (Supplementary note 1, Figure S5), top compartments with central channels of 300 µm were used for following experiments.

Features in the bottom compartment had a cross section of 1800 µm (width) x 293 µm (height) and consisted in three different layers. The three layers were realized sequentially: gap layer (height: 43 µm), pillars layer (height 100 µm) and VBV necking layer (height 150 µm). The ratio between the height of the pillar layer and that of the gap one defines the entity of the compression. Pillars in the compression chamber layer, positioned 30 µm apart, were designed with a T cross section, each arm of the shape being 300 µm long and 100 µm wide. The T-shaped cross section was chosen to minimize the pillars’ outward bending upon compression. The distance between two adjacent pillars was selected to confine 3D constructs during injection and polymerization phases, as well as to limit their lateral expansion upon compression. T’s tails, which face the culture medium channels, were rounded to avoid air entrapment during the medium filling phase. The central gel channel had a height 143 µm, and a width 300 µm. The necking connecting top and bottom compartments had a width of 100 µm and a height 150 µm.

The actuation chamber layer was designed so that three actuation chambers could be connected with the same actuation inlet (Figure 2A, D). Actuations chambers had a cross section of 3397 µm (width) x 50 µm (height). Six rows of round pillars (diameter 28 µm) were introduced to prevent the chambers from collapsing.

Top compartment and actuation chamber were realized pouring PDMS on master molds, to get heights of roughly 3 mm and 1.5 mm, respectively. PDMS stamps were then detached from the master molds, cut, and appropriate holes bored for hydrogel inlets (diameter 1mm), culture medium reservoirs (diameter 4 mm or 3 mm), and actuation tube inlet (diameter 1.5 mm).

The bottom compartment layer was realized pouring a small quantity of PDMS (roughly 4 ml) on the master mold and then spinning the master itself so that a thin PDMS layer would cover everything but the top surface of the necking geometry. The spinning procedure consisted in a 5 second ramp to 500 rpm, held for 15 seconds and followed by 30 seconds at 230 rpm.

PDMS stamps of the top culture chamber were bonded directly on this thin spinned PDMS layer before removing it from the master mold, to ease its handling. Subsequently, holes for hydrogels’ inlets and outlets and for culture medium reservoirs (3mm) were bored through both layers. A thick PDMS slab was bonded on the unpatterned membrane and a hole (1.5 mm) bored through both layers in correspondence of the inlet of the actuation chamber layer. The increased thickness provided by the slab assures retention of the tube providing the mechanical actuation pressure. Actuation chamber and actuation membrane (plus PDMS slab) were bonded first and united with the top compartment-bottom compartment assembly in a second moment. All procedures were performed treating surfaces to be united with air plasma (Harrick plasma) and bringing them in conformal contact for at least 30 min at 80°C, to achieve irreversible bonding. Devices were sterilized through autoclavation and further cured at 70 °C for at least 24h. This final curing step was demonstrated to minimize leachates from uncured PDMS.^[1]^ Single culture CoC microfluidic devices were produced as previously described.^[2]^

# Finite Element (FE) computational modelling.

A FE model was introduced to (i) confirm the application of two different and defined strain levels in the constructs hosted in top and bottom compartments, and (ii) estimate the effective strain field applied when compressing mature cartilaginous constructs.

Preliminary numerical simulations (Figure S8A-D) were performed considering both top and bottom compartments as filled with only the PEG based hydrogel. Subsequent computations were executed using the mechanical properties of mature cartilaginous constructs obtained after 14 days of hACs chondrogenic differentiation in the device (Figure 3C, D).

Cartilaginous constructs/PEG hydrogels were defined as having a BPE constitutive relation, which describes the mechanical response of a homogeneous continuous constituted by an elastic solid phase and an incompressible inviscid liquid phase. The BPE model accounts for the strain and time-dependent constitutive behavior due to the interaction between the two phases, but neglects the intrinsic viscoelasticity of the solid phase.^[3]^ As such, it underestimates short term-reaction forces of cartilage-like constructs and hydrogels upon compression but allows an accurate prediction of their strain field.^[3,4]^

Abaqus 6.14 (Abaqus FEA: Dassault systems), established as suitable to describe the biomechanical behavior of biphasic tissues,^[4]^ was adopted in computations. Given the repetitive pattern of the device inner geometry, in order to reduce the computational cost, a minimal unit volume constituted by the PDMS region corresponding to two T-shaped posts facing each other and the constructs volume in-between was adopted in simulations (Figure 3A). The volume of cartilaginous constructs/PEG hydrogels was divided in two regions to simulate the presence of two different layers (Figure 2A, green and red areas). The PDMS region was described as comprehensive of two posts in the bottom compartment, the VBV necking geometry, and the corresponding narrow canals volume in the top compartment.

Given the symmetries presented by the chosen volume, this repetitive unit was further reduced to a ROI constituted by half of a post and a quarter of the hydrogel using cinematic boundary conditions. The final region used in simulations is the shaded one in Figure 2A. It has a thickness of 165 μm along Y, accounting for half a post and half of the hydrogel region between posts (nominally 30 μm). The total height of the PDMS portion was 400 μm, given by 100 μm of the post, 150 μm of the necking area, and 150 μm of the top compartment. The hydrogel region was divided as follows: bottom construct (height 143 μm), and VBV necking plus top construct (height 150 μm + 150 μm respectively). Edges of PDMS structures were rounded with a 9 μm curvature radius according to measurements performed on physical devices.

The PDMS region was represented using either twenty-node quadratic elements with hybrid formulation (C3D20H elements: Abaqus) or ten-node quadratic modified tetrahedral elements with hybrid formulation (C3D10MH elements: Abaqus). Hydrogels were meshed using eight-node linear hexahedral elements with hybrid formulation and trilinear pore pressure (C3D8PH elements: Abaqus), as required for porous materials. The characteristic dimensions of the elements varied across the hydrogel volume. Smaller elements were adopted in the lower part of bottom constructs, which are subjected to higher strain levels (Figure S8A). The dimensions of PDMS elements varied according to the one of the hydrogel elements at the interface. A mesh sensitivity analysis was conducted to assure the consistency of the solutions (Figure S8B). The ratios between the dimensions of the elements in the different areas were kept constant, varying the total number of the elements. A final total number of 51282 element was adopted for the hydrogel region, using elements with average characteristic dimensions of 12 μm, 5 μm, and 3 μm in the different regions of the hydrogel volume. A total of 32930 elements was adopted to describe the PDMS parts.

Interactions between model parts were modelled using a surface-to-surface contact. A hard contact between cartilaginous constructs/PEG hydrogels and PDMS was modelled assuming perfect tangential lubrication. The top surface of the PDMS compartment was fixed with an Encaster boundary condition to model its continuity with the thick PDMS top compartment layer.

Displacements along the Z direction were impeded on the top surface of the hydrogel compartment, simulating the presence of the PDMS ceiling. A zero-pore pressure was assumed on the hydrogel lateral portions initially not in contact with the PDMS, to allow fluid outflow.

PDMS was described as an hyperelastic solid with a Mooney-Rivlin strain energy function^[2,5]^ with the equation below:

|  | $W=C_{1}\left( I_{1}-3 \right)+C_{2}\left( I_{2}-3 \right)+\frac{1}{D} {(J-1)}^{2}$ | (1) |
| --- | --- | --- |

Where I_1_ and I*_2_* are the first and second invariant of the right Cauchy-Green strain tensor C, J is the determinant of the deformation gradient tensor F, representing the ratio between the deformed and initial volumes of the object, and C_1_, C_2_, and D are the constitutive parameters of the model. C_1_ and C_2_ were assumed, respectively, equal to 254 kPa and 146 kPa, as reported in the literature for PDMS with a 10:1 base to curing agent ratio.^[5]^ D was set equal to zero, assuming a perfectly incompressible material.

As introduced, hydrogel and cell laden constructs were described with a BPE constitutive behavior. Poisson’s ratio was fixed at 0.33, as reported for similar materials,^[6]^ the specific weight of the permeating fluid was set to 9.956 x 10^-6^ N mm^-3^.^[7]^ The description of a BPE model in Abaqus makes use of the specific material permeability, defined as $K_{s}=\gamma_{w}k$, where $\gamma_{w}$ is the permeating fluid specific weight and k is the absolute permeability. Ks was set to 3x10^-4^ mm s^-1^, as previously done for similar hydrogels.^[7]^ The initial void ratio *e*=dVw / dVg, where dVw is the volume of the fluid phase and dVg is the volume of the solid phase, depended on the considered material. An initial value of 45 was adopted for the 2% dry mass PEG-based hydrogel formulation; an *e* value of 7.5 was used for mature cartilaginous-like constructs, accounting for the presence of both hydrogel initial dry mass and cellular volume fraction. Complete fluid saturation was assumed on all occasions.

The E moduli of PEG-based hydrogel and cartilaginous constructs, measured through IT-AFM, were set, respectively, to 1.9 kPa and 3.66 kPa.

Abaqus’ solid consolidation option was adopted to perform a transient analysis, implementing an automatic $\Delta t$ incrementation with a minimum time increment of 10^-8^ seconds and a maximum pore pressure incrementation allowed for time increment of 10^-5^ MPa. The volumetric strain compatibility tolerance for hybrid elements was set to 10^-4^. Compression was applied imposing a 43 μm vertical displacement of the hydrogel bottom surface, mimicking the deflection imposed by the membrane in the physical device. Displacement was ramped in a time frame of 0.5 seconds, accounting for the 1 Hz frequency adopted in cyclical mechanical stimulation during experiments.

Strain fields were evaluated through nominal strain components along the X, Y, and Z directions, which were calculated according to Abaqus definition as:

|  | $\varepsilon^{N}=\boldsymbol{V}-\boldsymbol{I}= \sum_{i=1}^{3} \left( \lambda_{i}-1 \right)n_{i}n_{i}^{T}$ | (2) |
| --- | --- | --- |

Where $\boldsymbol{V}=\sqrt{\boldsymbol{F}\boldsymbol{F}^{\boldsymbol{T}}}$ is the left stretch tensor (F being the deviatoric deformation gradient), $\lambda_{i}$ are the principal stretches, and $n_{i}$ are the principal stretch directions in the current configuration.

The overall strain field was evaluated through Nominal strains (NE) along the principal directions X (i.e. NE11), Y (i.e. NE22), and Z (i.e. NE33). Mean nominal strains in bottom and top compartments were calculated averaging the elements strains calculated in the centroid. To compensate the bias introduced by the usage of higher mesh densities in certain model regions (Figure S7A), the average strains were calculated separately for each of the areas with different mesh dimensions and subsequently averaged, weighting for the volumetric fraction of the areas.

# Single-cell data analysis

cDNA reads were aligned to the ‘hg38’ genome using Ensembl 104 gene models employing the STARsolo tool (v2.7.10a) with default parameter values exception made for the following ones: soloBarcodeReadLength = 0, clipAdapterType = CellRanger4, outFilterType = BySJout, outFilterMultimapNmax = 10, outSAMmultNmax = 1, soloType= CB_UMI_Simple, outFilterScoreMin = 30, soloCBmatchWLtype = 1MM_multi_Nbase_pseudocounts, soloUMIfiltering = MultiGeneUMI_CR, soloUMIdedup = 1MM_CR, soloCellFilter = None, soloMultiMappers = EM, soloCBstart = 1, soloCBlen =1 6, soloUMIstart = 17, soloUMIlen = 12.

Merged samples were demultiplexed based on present single nucleotide polymorphisms (SNPs) using the cellsnp-lite^[8]^  and vireo^[9]^ tools. BAM files with mapped reads and the SNP database HapMap 3.3 (<https://www.sanger.ac.uk/resources/downloads/human/hapmap3.html>) were used as input. Resultingly, cells were either assigned to a particular genotype of individual donors or not assigned to any genotype. By comparing the genotypes of hACs single cultures and those of cells from co-cultures, we were able to identify and exclude residual bmMSCs from downstream analyses.

A total of 24579 cells from 4 different human donors passed quality control steps and underwent further analyses. Subsequent analysis steps were performed using R (v4.2.0). Cells were clustered based on detected SNPs by building a shared nearest-neighbour (SNN) graph (k=10) ^[10]^ and using the ‘cluster_louvain’ method from the R igraph package.^[11]^ Most clusters were composed of cells of the same genotype. A cluster of cells that was characterized by mixed genotypes was assigned as composed of low-quality cells; a cluster of cells with no genotype was assigned as composed of doublets. Cells from these clusters, as well as residual cells from bmMSCs donors used for OCU-on-Chip constructs, were removed from further analyses. The final data set consisted of 24579 cells.

Multiple Bioconductor (v3.15) packages including DropletUtils (v1.16.0), scDblFinder (v1.10.0), scran (v1.24.1), scater (v1.24.0), scuttle (1.6.2) and batchelor (v1.12.3) were applied for the gene expression analyses of remaining cells. Analyses were mostly performed following the steps of the workflow presented at <https://bioconductor.org/books/release/OSCA/>. Raw counts were normalized by deconvolution.^[12]^ The function ‘computeDoubletDensity’ did not point at any group of cells, which could be potential doublets, confirming the efficiency of removing doublets while demultiplexing samples.

The most variable genes were derived by applying the function ‘getTopHVGs’ with the following parameters values: fdr.threshold = 0.01 and var.threshold = 0.1. The expression of the most variable genes was then used to perform principal component analysis. Principal components were corrected by removing the batch effect based on the genotype (i.e. the effect of individual donors). Afterwards the ‘runUMAP’ function from the Scater package was applied (with default parameters) to the corrected principal components to perform UMAP dimensionality reduction, which was adopted to visualize single cells on two dimensions.

Cells were then re-clustered based on the corrected principal components coordinated by building a shared nearest-neighbour (SNN) graph (k=100) and using the ‘cluster_louvain’ method. The ‘scoreMarkers’ function of the scran package was applied to find clusters characteristic marker genes. The standardized log-fold change across all pairwise comparisons ‘mean.logFC.cohen’>1 was used as the significance threshold. Marker genes common for two or more clusters were removed from the analysis.

The dataset was subjected to cell-type annotations using the Bioconductor package SingleR (v1.10.0). Publicly available annotated scRNA-Seq data sets^[13,14]^ were used as reference.

Differential expression analyses in static and mechanically stimulated samples, considering both single culture hACs and hACs that were co-cultured in OCU-on-Chip constructs, were performed aggregating single cells into *in silico* bulk samples created by summing up counts per gene across all cells belonging to a particular cluster, condition, and replicate.^[15]^ The function ‘filterByExpr’ excluded lowly expressed genes from the analysis. Combinations containing at least 20 cells underwent analysis with edgeR (3.38.4). Genes were considered as differentially expressed if they had a false discovery rate (FDR)<0.05. A gene ontology (GO) analysis of differentially expressed genes was performed using STRING Database (<https://string-db.org/>)^[16]^, Version 11.5.

Gene set enrichment analysis (GSEA, v4.2.3) was applied to differentiate pathways affected by mechanical loading in hACs form single cultures and OCU-on-Chip co-cultures.^[17]^ Reported pathways were based on the KEGG database ([http://www.kegg.jp](http://www.kegg.jp/)). For each contrast, expressed genes were ranked by their log-fold changes as reported by edgeR. For each pathway and each comparison, the FDR accounting for the significance of the enrichment was calculated. A pathway was reported if the corresponding FDR was less than 0.1.

# **SUPPLEMENTARY FIGURES**

**
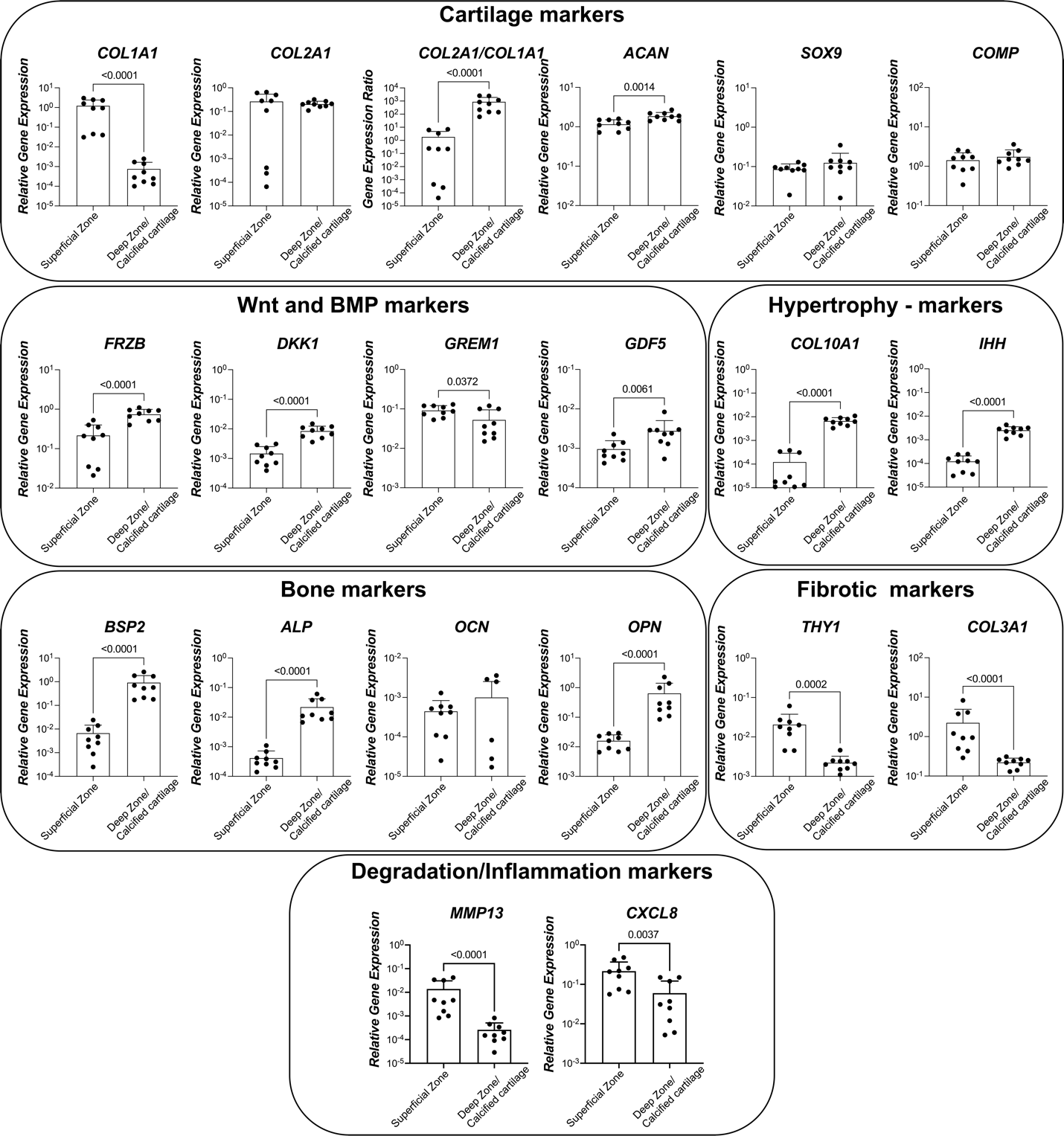
**

**Figure S1. Gene expression of superficial and deep zone/calcified cartilage from human knees.** The gene expression of knee cartilage from clinical OA patients was quantified through RT-qPCR. Cartilage samples were harvested from the femoral condyles of patients undergoing knee replacement for OA, from areas without signs of cartilage degradation or surface fibrillation. Cartilage from the superficial zone and from the deep zone/calcified cartilage were collected and analyzed separately (n=3 patients, for each patient n=3 separate areas were harvested). Statistical significance was determined by paired t-test (normal populations) and Wilcoxon test (non-gaussian populations). (Adjusted) p-values < 0.05 are reported on the graph. Expression levels of all genes were normalized to GAPDH expression. Values are reported as mean + s.d. Populations’ normality was assumed if both Shapiro-Wilk and Kolmogorov-Smirnov tests resulted positive. *COL2A1/COL1A1*, *ALP*, *COL10A1*, and *MMP13* data are the same as the ones reported in Figure 1D.


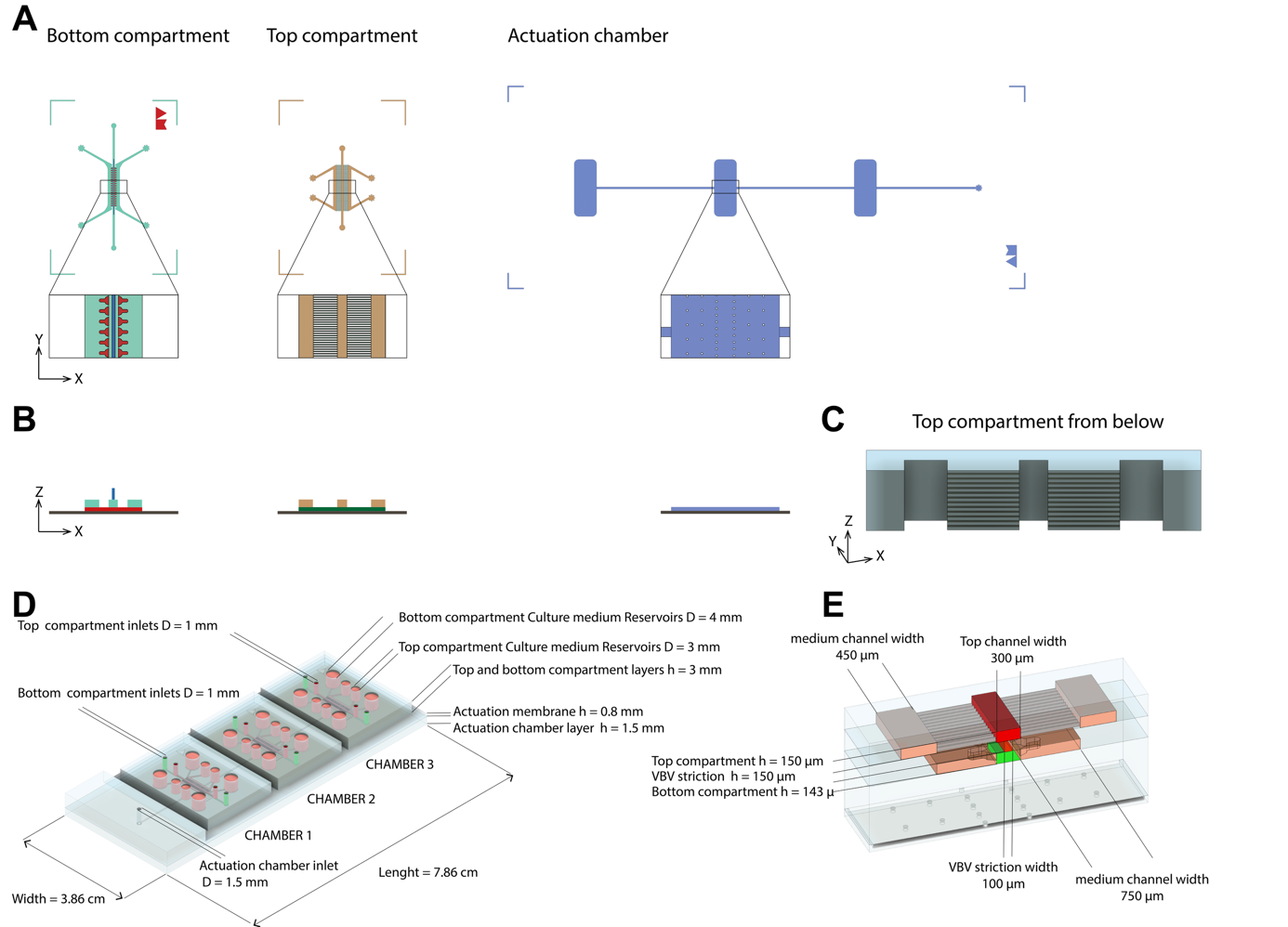


**Figure S2. Device geometry.** A, B) Layouts of master molds used to produce the device (bottom and top compartments show one of the three functional units). The different layers used during the multi-layer photolithography process are color-coded. The bottom compartment was realized through three layers: gap layer (in red), pillars layer (in aqua green), and VBV necking layer (in blue). The top compartment was realized through two layers: the narrow canals layer (in dark green), and the central channel layer (in beige). The Actuation chamber was realized through a single layer, portrayed in purple. C) Schematic detailing the inner structure of the device top compartment and of the narrow canals as seen from below. D) and E) Schematics detailing the external and internal dimensions of the device.


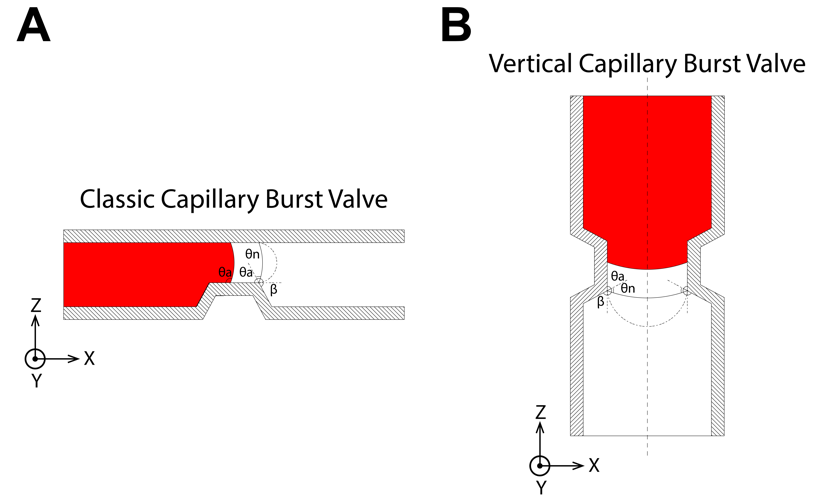


**Figure S3. Classic CBV and VBV working principle.** A) Classic CBV schematization; B) Vertical CBV (i.e., VBV) schematization. Assuming a liquid is moving in a narrow channel along the Y direction (i.e. perpendicularly to the represented XZ plane), it can be prevented from invading a neighboring channel through a striction, or a necking, followed by a sudden aperture, which according to the Young-Laplace Equation^[18]^ creates an increase in the pressure required for the fluid to proceed. This geometry is usually defined as a CBV. Given the limited contribution of gravitational forces with respect to the surface tension at the microscale, it was possible to translate the CBV to a vertical geometry. The walls of the channel are indicated by the etching, the advancing liquid is portrayed in red. The advancing contact angle is indicated with θa, the angle change caused by the valve with β, and the contact angle that the liquid interface assumes with the new wall after the striction with θn = θa – β. The interface required for the liquid to have a contact angle θa with the new interface so to resume its advancement is indicated by the dashed line.


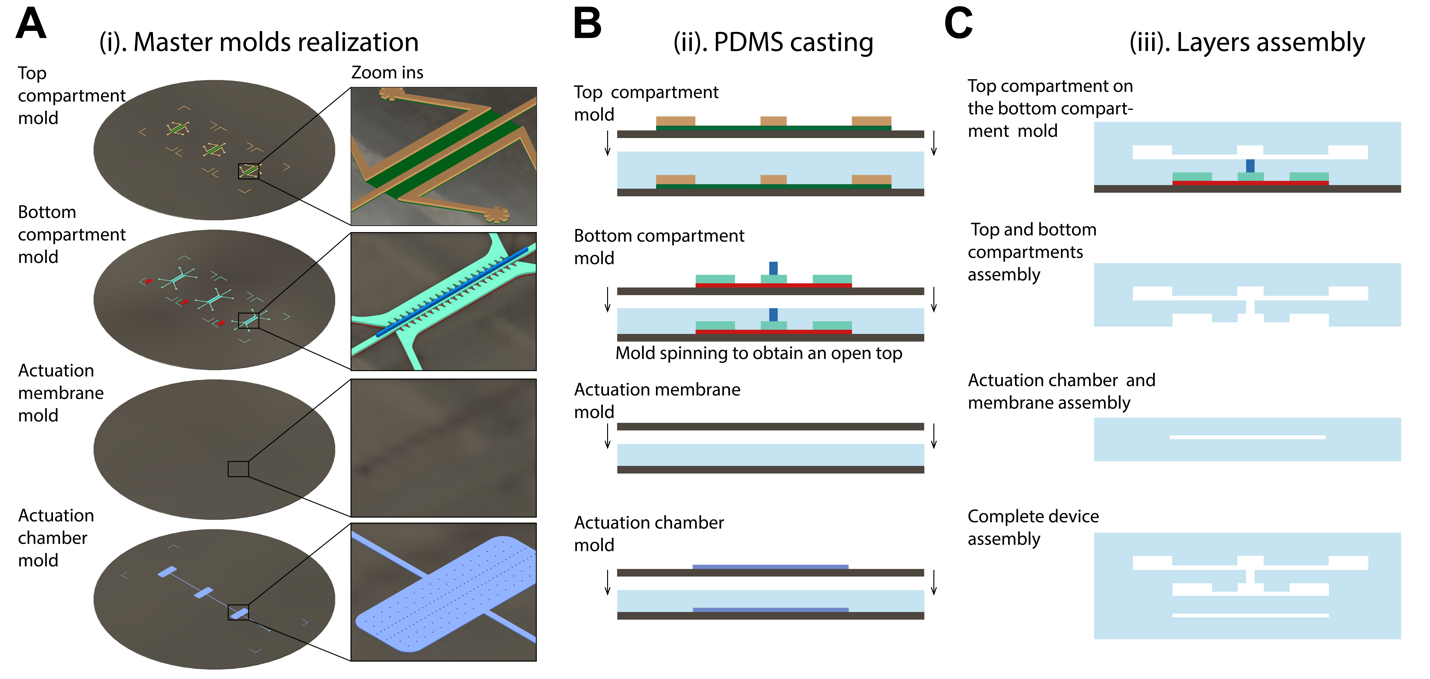


**Figure S4. Device fabrication protocol*.*** A) Schematic representing the master molds required to produce the device’s PDMS layers. The insets highlight the photoresist structures required to obtain the final molds. The actuation membrane was constituted by a simple PDMS layer, produced from a plain silicon wafer. B) Schematic depicting the PDMS casting procedure. The bottom compartment layer was obtained by spinning the mold with a spin coater, to obtain an open top in correspondence of the VBV necking. C) Schematic of the layers’ assembly. The top compartment layer was peeled off the mold and bonded to the bottom compartment layer while this was still attached to its mold. The top compartment - bottom compartment assembly was then peeled of the mold altogether (thus obtaining the VBV tilted-H geometry) and bonded with the actuation chamber - membrane assembly. This last procedure was repeated for each of the three functional units

# **Supplementary note 1: Compartments’ alignment optimization**

Achieving through photolithography monolithic closed channels or partially free-standing structures (like the VBV) requires complex and error prone methodologies. These include the use of sacrificial materials^[19]^ or multi-layer processes based on the selective cross-linking of photoresist strata.^[20]^ Achievement of the VBV structures with the abovementioned processes was attempted and abandoned due to the unshapely geometry of obtained features (data not shown); final devices were produced aligning multiple PDMS layers as described in Figure 2D.

Given the necessity to align top and bottom compartments to produce each device, the precision, accuracy, and efficiency of the alignment procedure were assessed and optimized coupling top compartments with different dimensions (central channel width of 300 µm, 400 µm, and 500 µm) with bottom compartments with a fixed central channel measuring 300 µm. Manually positioning the two compartments might result, in fact, in partially aligned chambers (Figure S5A). A wider central channel in the top compartment facilitates centering, reducing the risk of not covering the VBV necking area—the sole contact surface between compartments. However, it also decreases the portion of the top construct in direct interface with the bottom compartment.

The alignment precision was evaluated using top-view images of assembled devices. The portion of each central channel which was aligned with the corresponding counterpart was calculated as:

|  | $Overlapping area ratio\%=\frac{Overlapping area}{Total layer area}*100$ | (3) |
| --- | --- | --- |

Where the Overlapping area was defined as the area between the two innermost pillar/narrow canals rows in top view images (regardless of which layer they belonged to), while the Total layer area was measured as the distance between the pillar rows of each layer (which might slightly differ from the nominal values due to the imaging angle). The product of the Overlapping area ratio% of the two layers was adopted as a final criterion to evaluate which configuration maximized the production success.

Resulting values were respectively 82% ± 8 %, 75% ± 11%, and 77% ± 6% for the 300 μm, 400 μm, or 500 μm top culture chambers (Figure S5B), being statistically significantly higher in the narrower device version. The top chamber with a central channel of 300 μm was therefore adopted in the final design. The assessment was performed with n=6 devices for each configuration. Devices in which the VBV necking area did not overlap with the top compartment central channel were excluded from the analysis. Considering n=30 devices, less then 10% of devices were discarded, proving the practicability of the fabrication procedure.


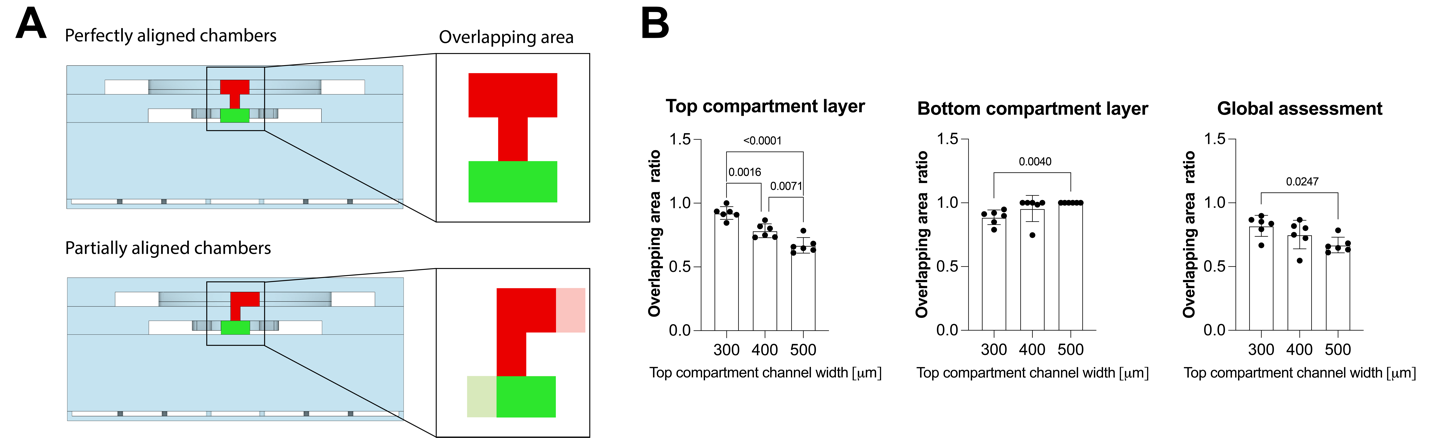


**Figure S5. Top compartment’s alignment: precision assessment and optimization.** A) Side view schematization of perfectly aligned (top) and partially aligned (bottom) top and bottom compartments. In the insets, the portion of the aligned central channels is represented in bold colors, non-overlapping areas are depicted in fainter colors. B) Three top compartment versions were realized, with the central channel measuring respectively 300 µm, 400 µm, and 500 µm (while the compression chamber gel channel remained of 300 µm) to determine if a wider top channel was necessary to achieve overlapping top and bottom compartments. Graphs represent the ratio of the overlapping area with respect to the chamber area calculated according to equation (3) (N=6 devices for each dimension). Populations’ normality was assumed if both Shapiro-Wilk and Kolmogorov-Smirnov tests resulted positive. Statistics by Ordinary One-Way Anova with Tukey’s multiple comparison test for normal populations and Kruskal-Wallis test with Dunn’s multiple comparison test for non-normal populations. Results are reported as mean ± s.d.

# **Supplementary note 2: Determination of devices’ actuation pressure**

The actuation pressure, i.e. the pressure necessary to obtain contact of the actuation membrane with the pillars in the bottom compartment, was determined as follows.

Actuation chambers were slowly filled with PBS, applying a mild continuous positive pressure (i.e. 0.2 Atm) until all air bubbles were removed. Subsequently, top and bottom compartments were filled with blue dye, both the central channel and lateral medium channels. At atmospheric pressure, pillars in the bottom compartment appear blue because of the color filling the gap between pillars and actuation membrane. When the membrane bends upward due to the increased pressure in the actuation chamber, the gap narrows and the pillars start appearing whiter, that is to say the Mean Grey Intensity Value increases. A correlation between the Mean Grey Intensity Value measured in correspondence of the pillars and the provided pressure could be established. Once contact is reached, a further increase in pressure does not correspond anymore to an increase in Mean Grey Intensity Value (i.e. there is a plateau). The actuation pressure was defined as the pressure at the beginning of the plateau in the Mean Grey intensity vs Pressure curve (i.e., 0.4 Atm, Figure S6A, B). At least three chambers were considered in analyses. For each chamber, the Mean Grey Intensity Value of three different pillars was measured and results averaged. Images were analyzed with Image J.


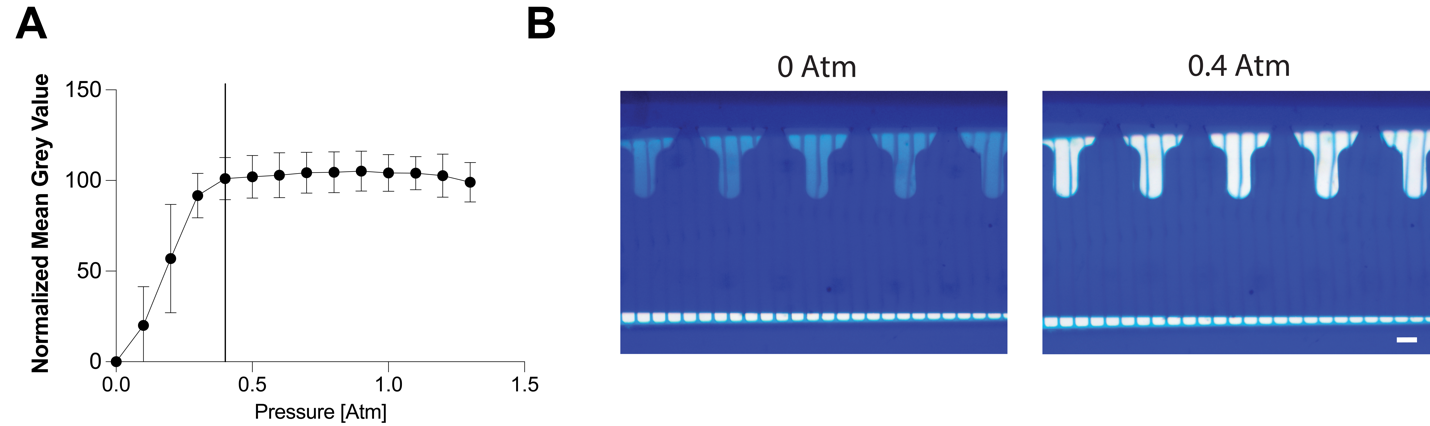


**Figure S6. Determination of the actuation pressure.** A) Normalized Mean Grey Intensity Value of T-shaped pillars versus actuation chamber pressure. The actuation pressure (i.e. the onset of the plateau) is indicated by the black vertical line. Results are reported as mean ± s.d. B) Examples of top view images of the device at atmospheric pressure (0 Atm) and at the actuation pressure (0.4 Atm). Scale bar, 100 µm.


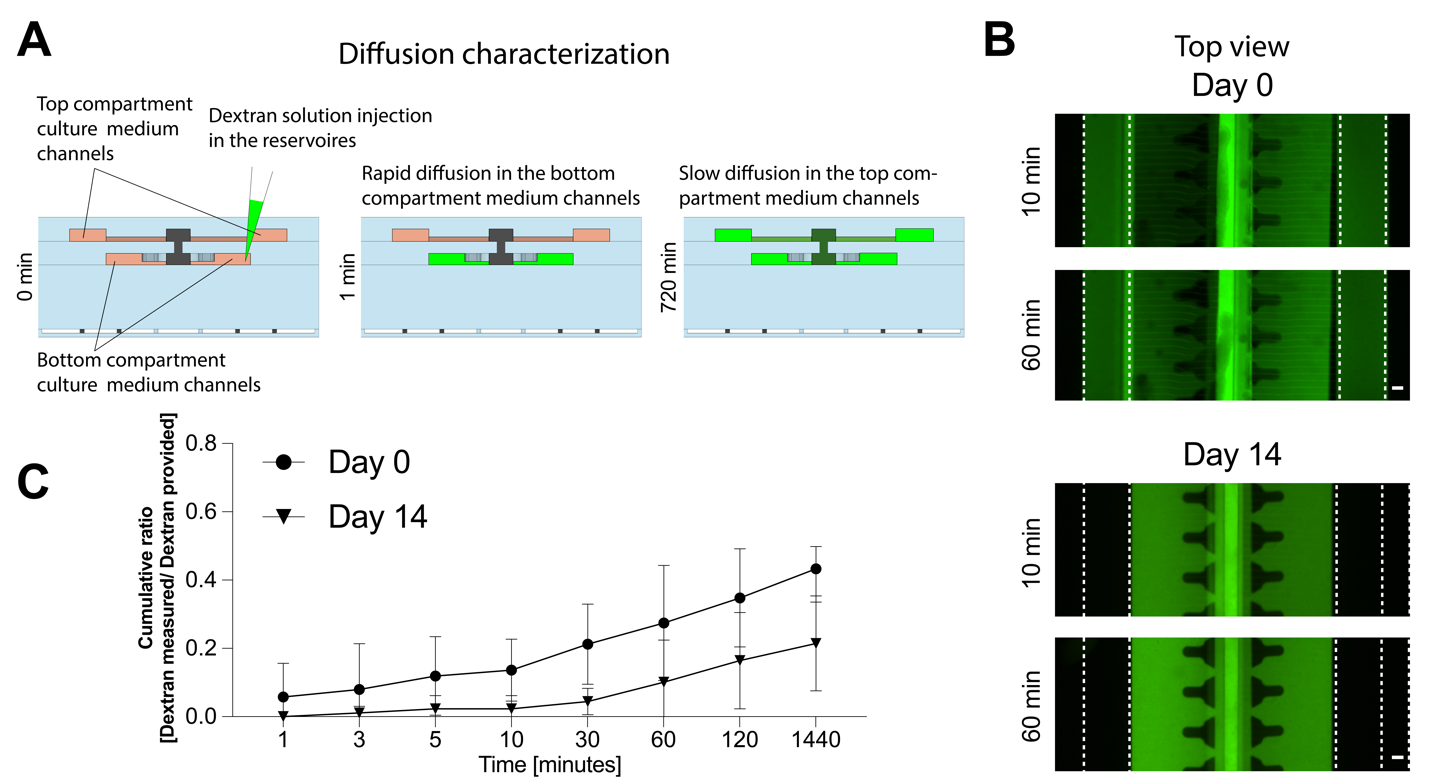


**Figure S7. Characterization of diffusion kinetics between top and bottom compartments.** A) Schematic of the experimental setup. The bottom channels’ culture medium reservoirs were filled with a dextran solution (20 kDa, 1 mg ml^-1^), and the dextran concentration in the top compartment culture medium reservoirs was measured at selected time points. The diffusion process was also observed though time lapse imaging. hACs were laden in 2% PEG based hydrogels and seeded in both top and bottom compartments’ central channels. The diffusion assay was performed either on the same day of hACs seeding (i.e. Day 0) or after 14 days of static maturation in chondrogenic medium (i.e. Day 14). B) Representative fluorescence images of FITC dextran diffusion after 10 mins and 60 mins from injection in the bottom compartment’s culture medium reservoirs (n=3 independently cultured samples per time point). Top compartment’s culture medium channels are highlighted by dotted lines. Scale bar, 100 μm. C) The diffusion rate of 20 kDa FITC-dextran was quantified by measuring FITC fluorescence intensity in the top compartment's culture medium reservoirs. Concentration over time was expressed as the cumulative ratio of dextran injected into the bottom compartment to the amount measured in the top compartment at selected time points (reaching 0.5 at equilibrium) (n= 3 independently cultured samples per time point). Cumulative ratios after 60 mins resulted of 0.27 ± 0.17 and 0.1 ± 0.12 for Day 0 and Day 14, respectively.


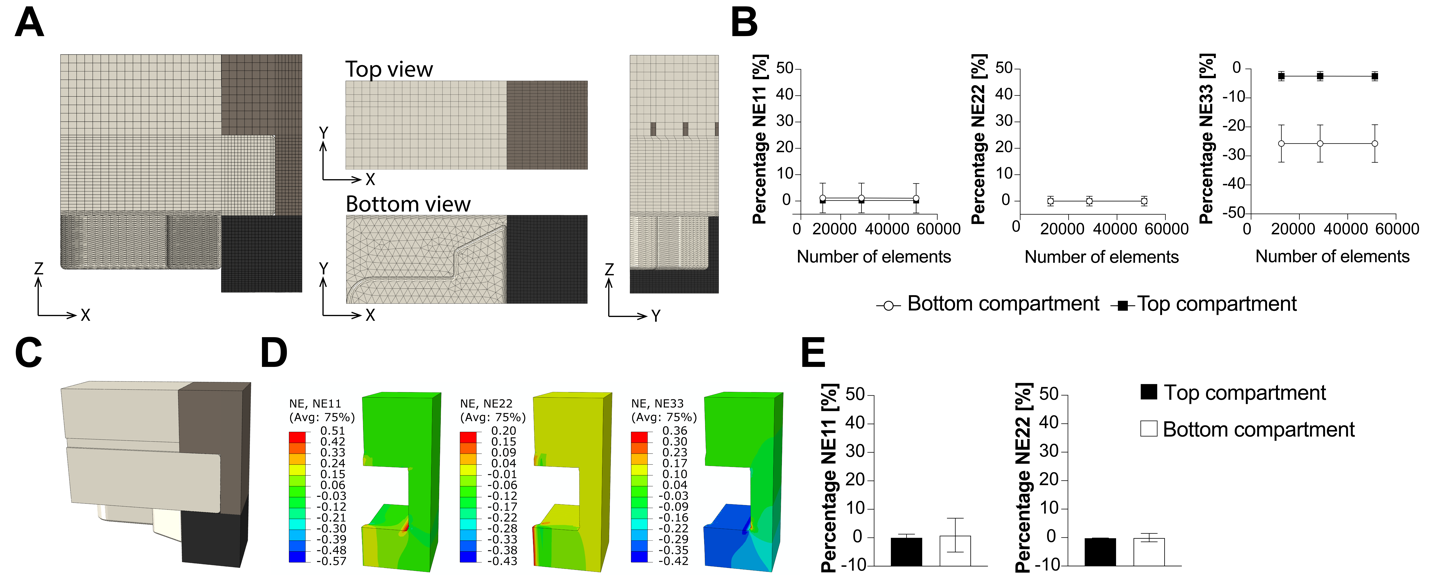


**Figure S8. FE model setup.** A) Orthogonal plane views showing 3D geometry and elements’ mesh used in computations. Images represent the Region of Interest (ROI) volume, i.e. the minimal volume used in computations. Symmetry boundary conditions were adopted to account for the geometry of the whole device. PDMS regions are depicted in light grey, the top construct is represented in grey, and the bottom constructs in black. B) Mesh sensitivity analysis: Nominal strains along the principal directions (i.e. NE11, NE22, and NE33) were evaluated in top (including the VBV necking area) and bottom constructs as a function of the elements’ number. Values, extrapolated from the elements’ centroids, are reported as mean ± s.d. C) Geometry of the ROI volume adopted in computations, complete of constructs (in dark grey and black) and PDMS structures (in light grey). D) Contour plots showing the nominal strains along the principal directions obtained using the 2% PEG gel properties (E= 1.9 kPa). E) Quantifications of NE11 and NE22 in top and bottom constructs referred to simulations reported in Figure 2C (E = 3.66 kPa). Values, extrapolated from the elements’ centroids, are reported as mean ± s.d.

**
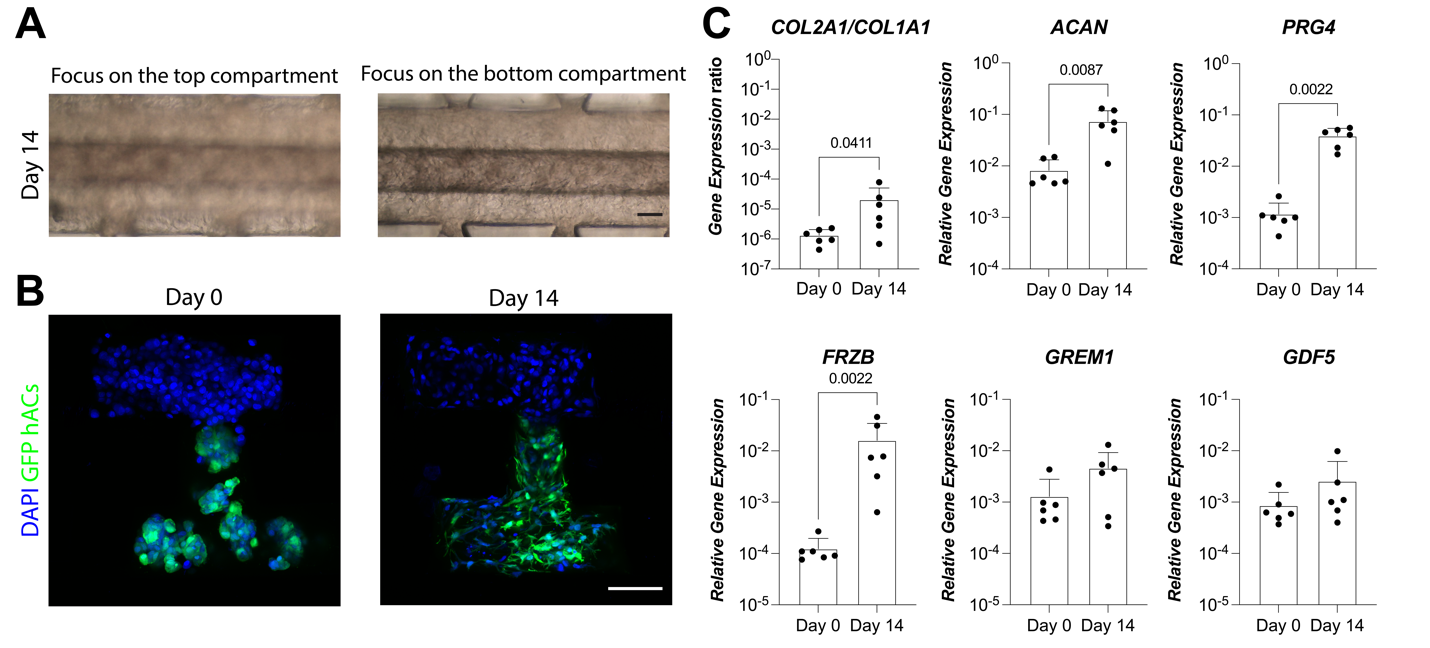
**

**Figure S9. Establishment of bi-layer mature human cartilaginous constructs on-chip.** A) Representative brightfield pictures of hACs statically cultured in devices for 14 days. After 2 weeks of culture, cells are still confined in the central channels. Similar results were obtained with more than 60 devices. Scale bar, 100 µm. B) Immunofluorescence images of constructs sections immediately after seeding (Day 0) or after 14 days of static culture (Day 14). Constructs’ layers have a direct interface in correspondence of the VBV necking and maintain a stratified structure throughput the culture period. hACs were seeded in the top compartment, GFP+ hACs in the bottom one (n=3 independently cultured devices from one donor for each compartment). Scale bar, 100 µm. C) Gene expression of known chondrogenic markers after 14 days of static culture in the OCU-on-Chip device. Gene expression was quantified through RT-qPCR (n≥6 independently cultured samples from n=2 donors). Statistical significance was determined by Mann-Whitney test. Populations’ normality was assumed if both Shapiro-Wilk and Kolmogorov-Smirnov tests resulted positive. p-values < 0.05 are reported on the graph. Expression levels of all genes were normalized to GAPDH expression, values are reported as mean + s.d.


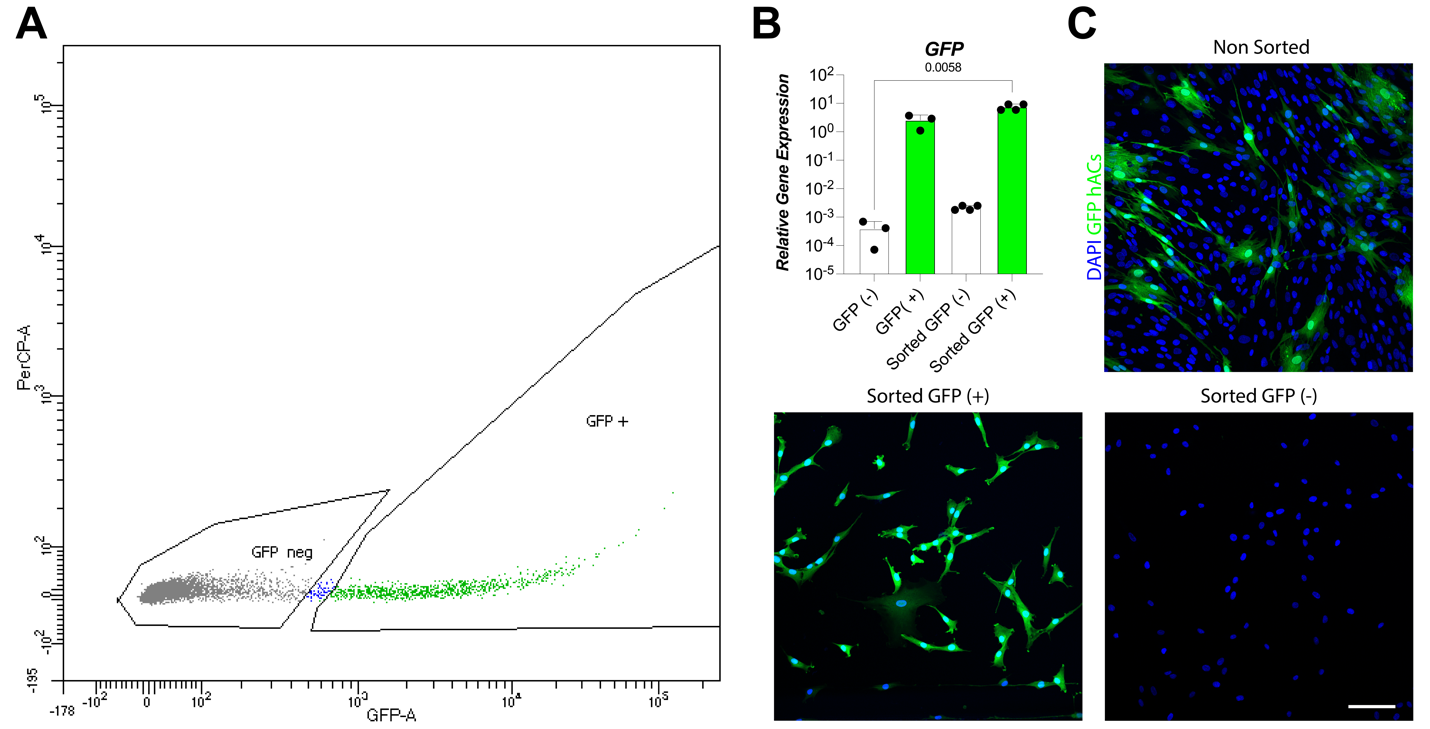


**Figure S10. hACs from top and bottom compartments can be separated based on GFP expression.** A) Example of populations selection during sorting. B) *EGFP* expression of sorted and pure populations as quantified by RT-qPCR. Pure populations, i.e. populations which were never in contact with each other, are indicated as GFP (+) and GFP (-), populations sorted after culture and constructs’ enzymatic digestion are indicated as Sorted GFP (-) and Sorted GFP (+) (n≥3 independently cultured samples from n=1 donor for each population). Statistics by Kruskal-Wallis test with Dunn’s multiple comparison test. Populations’ normality was assumed if both Shapiro-Wilk and Kolmogorov-Smirnov tests resulted positive. (Adjusted) p-values < 0.05 are reported on the graph. Expression levels of all genes were normalized to GAPDH expression, values are reported as mean + s.d. C) Representative immunofluorescence images of cellular populations obtained after constructs digestion. At least n=3 independent constructs from n=1 donor for each population were considered for each condition. Constructs were digested and sorted (or not) and cells plated in 2D on an IBIDI 8 well µ-Slide. Cells were left to adhere for 48 hours, then fixed and imaged. Scale bar, 100 µm.

**
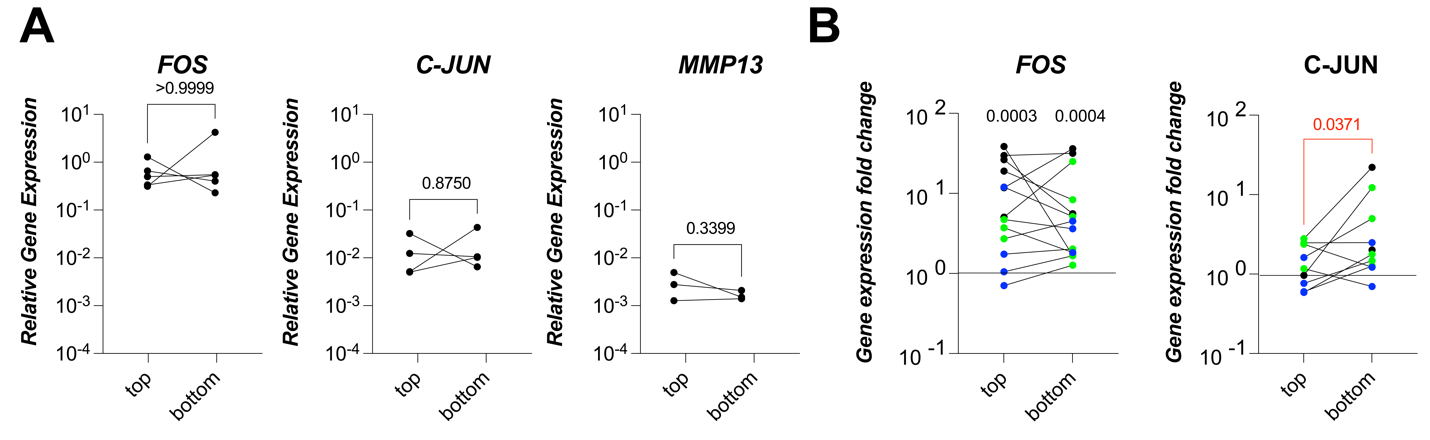
**

**Figure S11. Gene expression baseline of cartilaginous tissue in top and bottom compartments.** A) Cartilaginous tissues cultured statically in top and bottom compartment for 21 days do not show differences in gene expression as quantified by RT-qPCR (n≥4 independently cultured samples from n≥2 donors; samples cultured in the same device were compared directly). Statistical significance was determined by paired t-test for normal populations and Wilcoxon test for non-gaussian populations. (Adjusted) p-values are reported on the graph, no statistically significant differences (i.e., p-value < 0.05) were detected. Gene expression levels referring to top and bottom constructs coming from the same chamber are connected by black lines. Expression levels of all genes were normalized to GAPDH expression. Populations’ normality was assumed if both Shapiro-Wilk and Kolmogorov-Smirnov tests resulted positive. B) Replication of RT-qPCR graphs of figure 2G. Samples are color-coded depending on the adopted donor. GFP+ hACs are represented in green. Experiments were performed seeding GFP+ hACs in both top and bottom compartment, to exclude transduction-induced biases.

# **Supplementary note 3: Osteochondral biopsies IT-AFM measurements**

The nano/microscale mechanical properties of osteochondral interface tissues were measured as follows.

Full osteochondral biopsies were collected from explants of patients undergoing total (TKA, n=18) or unicondylar (UKA, n=4) knee arthroplasty. A total of 22 patients (9 females, 13 males; average age: 63 years ± 10 years) was considered. Full knee explants were placed in sterile plastic containers and submerged in phosphate buffered saline (PBS). After transportation (performed within 2 hours after surgery) samples were kept at 4 °C. Biopsies were obtained from the distal femur condyles through an 8G bone marrow needle. Using a surgical scalpel, biopsies were cut in half along their thickness to obtain a flat surface. Samples were positioned horizontally and carefully oriented so that the cut flat surface faced upward. IT-AFM indentations were performed on the lateral side of OCU biopsies, at room temperature, in degassed PBS.

Measurements were conducted using the ARTIDIS ADO (Automated Device Operation) AFM (ARTIDIS AG, Basel, Switzerland) as described in the Method section. Coupling of IT-AFM with brightfield microscopy allowed to correlate indentation maps with a definite location on the samples (Figure S12D).

Immediately after completion of IT-AFM measurements, osteochondral biopsies were fixed with 4% paraformaldehyde for 48 hours and decalcified with a 15% (w/v) ethylenediaminetetraacetic acid (EDTA) solution (Sigma). Samples were subsequently dehydrated and embedded in paraffin so that the first layers obtained during sectioning corresponded to the sample face probed with IT-AFM. Sections (5 μm thick) were cut through a microtome (Microm HM 340E). Safranin-O (Saf-O)/fast green staining (Fluka) was performed according to standard protocols, to visualize the glycosaminoglycan (GAG) content of the biopsies. Samples were graded according to the OARSI grading system.^[21]^ Only samples with a grade 1-3 were used for further analyses. Brightfield images adopted during the spot selection procedure were manually superimposed with Saf-O stainings (Figure S12E). Overlapped images were adopted to determine whether areas surrounding indentation spots were positive or negative for GAG.

The mechanical properties of osteochondral tissues vary in OA and depend on various unclear factors,^[22,23]^ making a complete assessment beyond the scope of this study. However, by grouping indentation spots based on the GAG positivity or negativity of their surrounding area, we identified distinct differences in E modulus at the osteochondral interface (Figure S12F).


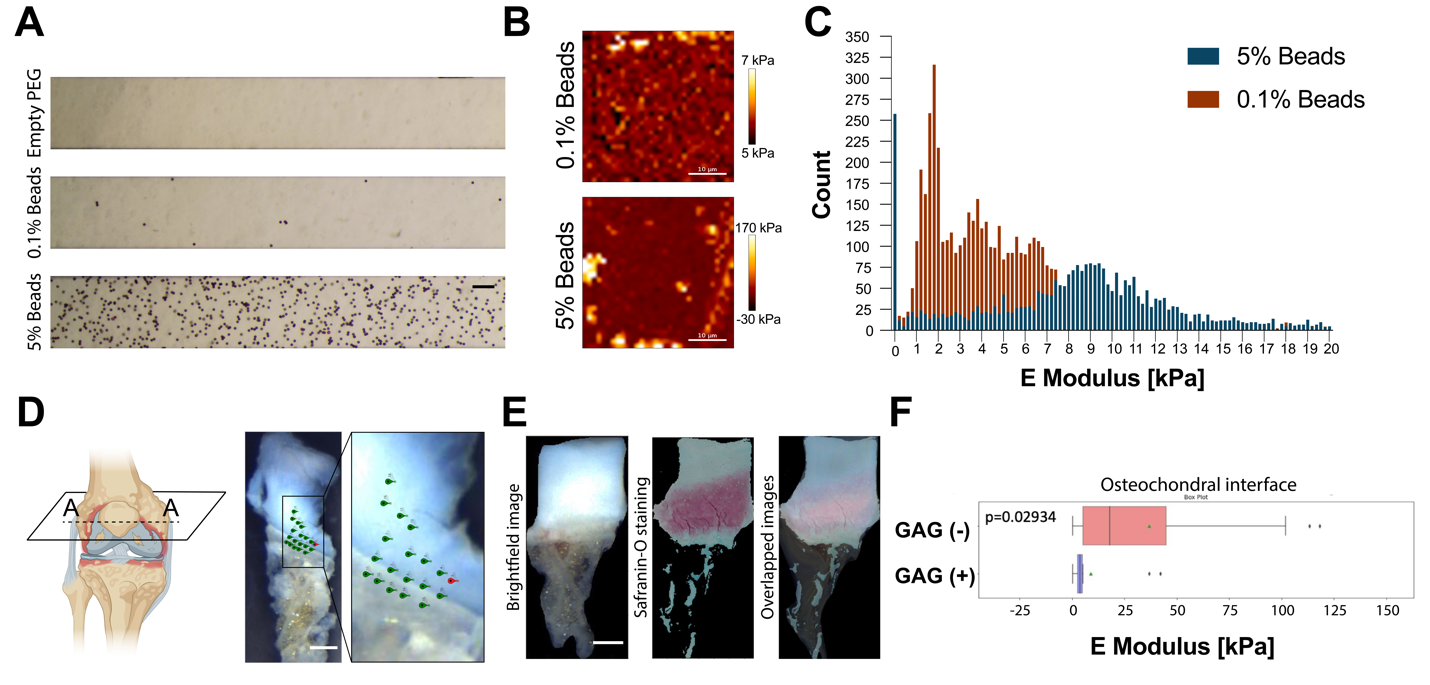


**Figure S12. Mechanical properties of beads laden hydrogels and OA osteochondral biopsies.** A) Brightfield images of the top compartment’s central channel seeded with empty PEG or with PEG hydrogels loaded with different volumetric fractions of polystyrene beads. Scale bar, 100 µm. B) Examples of IT-AFM indentation maps of beads laden hydrogels. E modulus values are color coded. Scale bar, 10 µm. C) Frequency histogram of gels’ E modulus as assessed through IT-AFM. D) Schematization of the experimental procedure adopted for IT-AFM measurements of OA patients’ osteochondral biopsies. Brightfield images indicate examples of selected indentation points. The zoom-in highlights the spots line (nr 6-10) at the osteochondral interface. Scale bar, 1mm. E) Example of Brightfield and Saf-O images used to determine the GAG content of areas near indentation points. Scale bar 1mm. F) Box plot of the E modulus of GAG positive and negative indentation spots. Statistics by paired t-test for normal populations and by Wilcoxon test for non-normal populations. Population normality was assessed trough Kolmogorov-Smirnov test. p=computed p-value (n= 15 donors). Results are plotted as median ± interquartile range, outliers are indicated by rhombi.


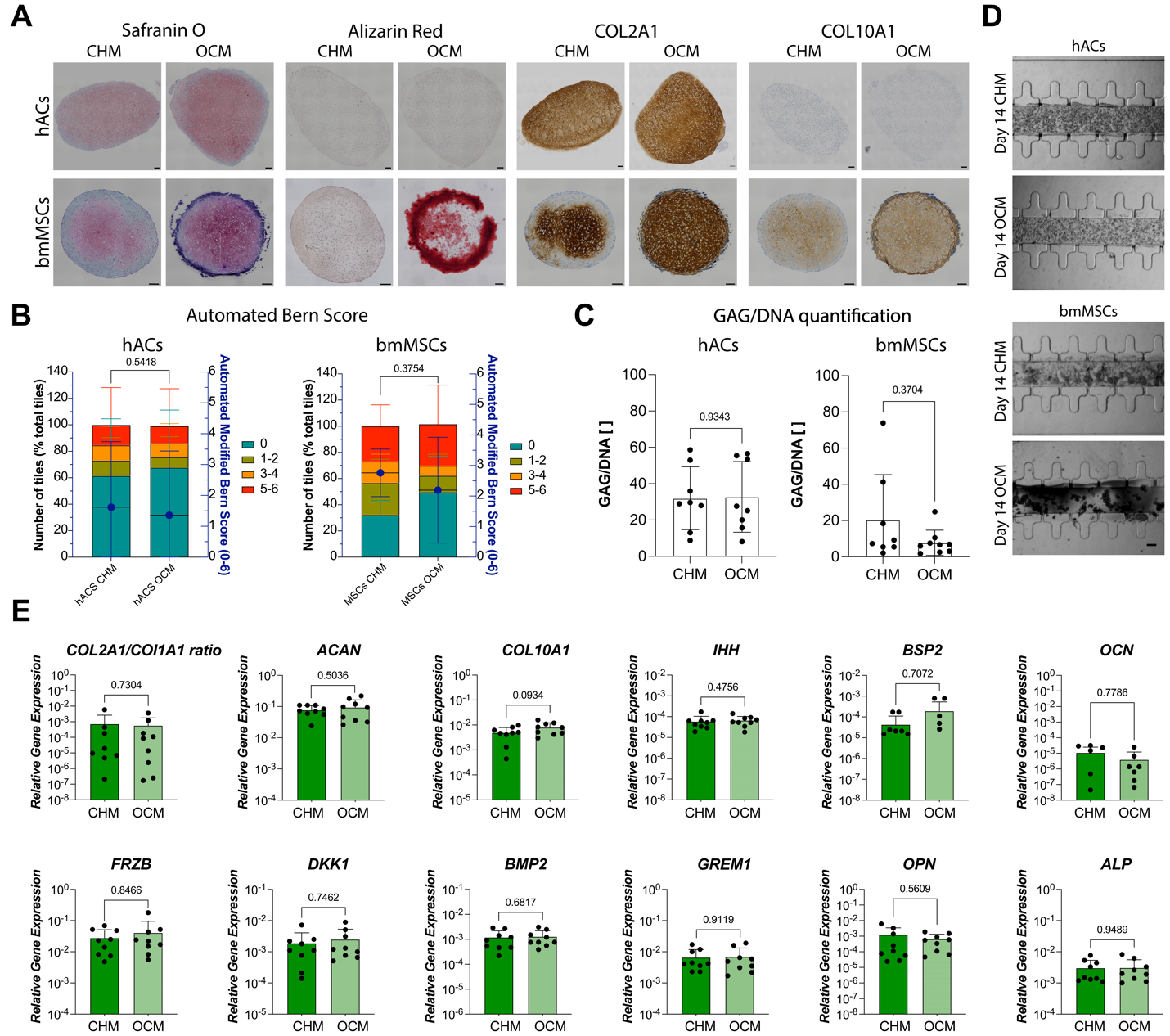


**Figure S13. OCM’s effects on hACs and bmMSCs.** A) Histological stainings of hACs and bmMSCs aggregates (i.e. pellet) after 14 days of culture. Safranin-O/fast-green staining was used to indicate GAGs deposition, Alizarin Red to stain calcium deposits, immunohistochemistry stainings of COL2A1 and COL10A1, respectively, as markers of hyaline and hypertrophic cartilage (n≥2 independently cultured samples from each of n≥5 donors). Scale bar, 100 μm. B), Grading of hACs pellets Safranin O/fast-green stainings based on the Automated Modified Bern score performed as described in.^[24]^ Images were divided in squared tiles of a given dimension; each tile was scored from 0 to 6 (according to positivity for Safranin-O staining and cell morphology) and an average score was computed. Automated scoring was performed adopting tiles of 224x224 pixels with a pixel dimension of 0.511 µm. Results are reported as mean ± s.d. Tiles percentages are color-coded according to their score, average values are indicated by blue dots (n≥2 independently cultured samples from each of n≥5 donors). Statistical significance was determined by Mann-Whitney test. C) GAG/DNA quantifications in hACs and bmMSCs pellets after 14 days of culture (n≥1 independently cultured samples from each of n=4 donors for each cell type). Statistical significance was determined by Mann-Whitney test. Results are reported as mean ± s.d. D) Brightfield pictures of hACs and bmMSCs constructs after 14 days of static maturation on-chip (n≥50 independently cultured samples from n≥5 donors). Scale bar, 100 μm. E) Assessment of OCM effects on hACs gene expression after 14 days of static culture in CoC devices. Gene expression was quantified through RT-qPCR (n≥9 independently cultured samples from n≥3 donors). Statistics by two-tail unpaired t-test for normal populations and by Mann-Whitney for non-normal populations. Expression levels of all genes were normalized to GAPDH expression. Values are reported as mean + s.d. For all graphs, populations’ normality was assumed if both Shapiro-Wilk and Kolmogorov-Smirnov tests resulted positive. No significant differences were detected between CHM and OCM.


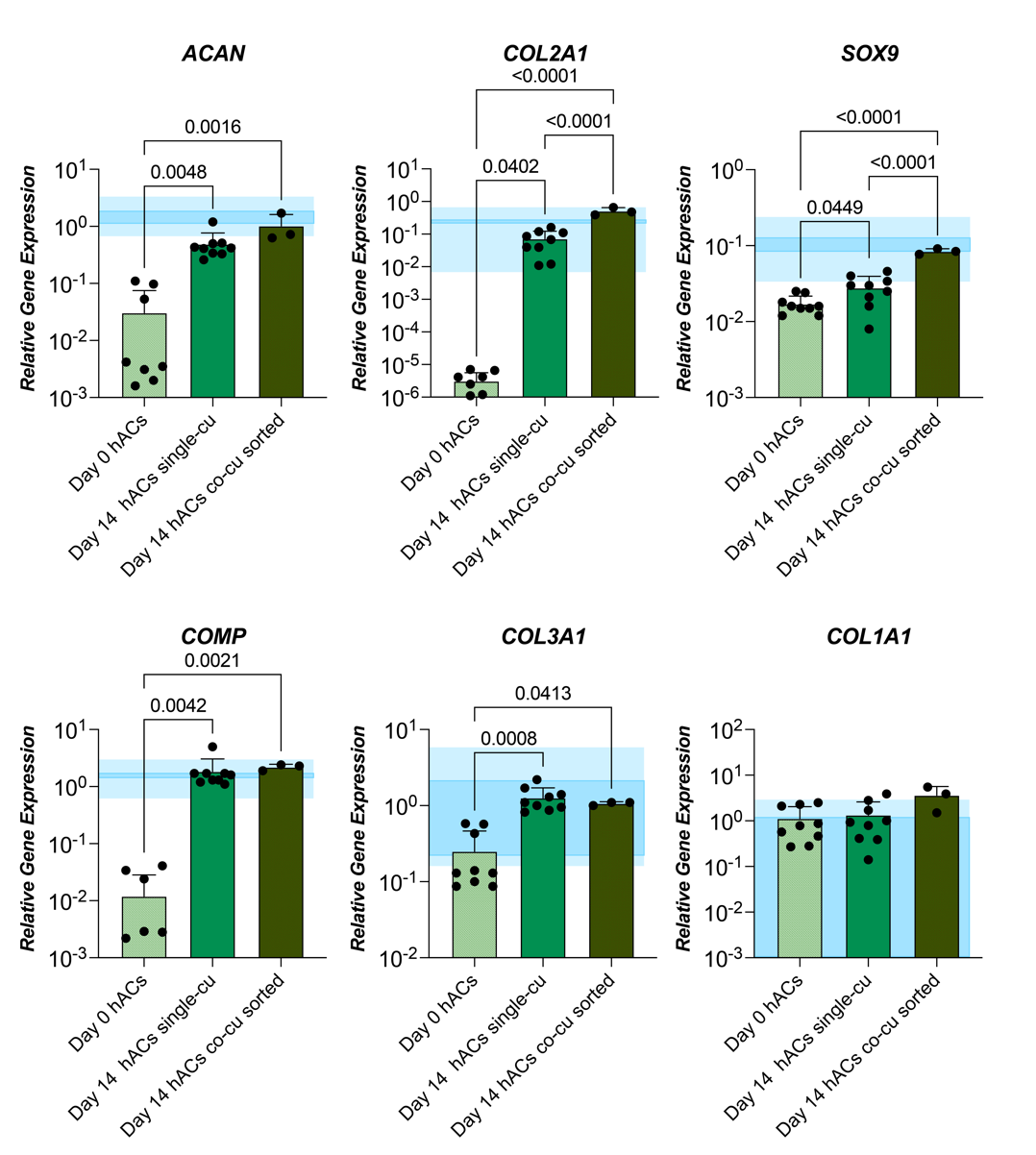


**Figure S14. Co-culture effects on hACs’ maturation.** RT-qPCR quantification of hACs’ gene expression from Day 0 (i.e. of 2D expanded hACs used to establish on-chip cultures), to Day 14. At Day 14, both hACs cultured in single cultures and sorted GFP+ hACs from OCU-on-Chip co-cultures were considered (n=9 independently cultured samples from n=3 donors, exception for the Day 14 hACs co-cu sorted condition for which n=3 independently cultured constructs from n=1 hACs donor were evaluated). Samples’ gene expression was also compared to the one of human knee cartilage (considering both the expression of cartilage superficial zone and deep zone/calcified cartilage), which is indicated by the cyan band on the graphs (the average range is indicated by the darker color, the standard deviation range by the lighter one). Statistical significance was determined by one way ANOVA with Tukey’s test for multiple comparisons for normal populations, and by Kruskal-Wallis test with Dunn’s post hoc test for multiple comparisons for non-Gaussian populations. Populations normality was assessed through Shapiro-Wilk and Kolmogorov-Smirnov tests. (Adjusted) p- values < 0.05 are reported on the graph. All genes expression was referred to GAPDH expression. Values are reported as mean + s.d.


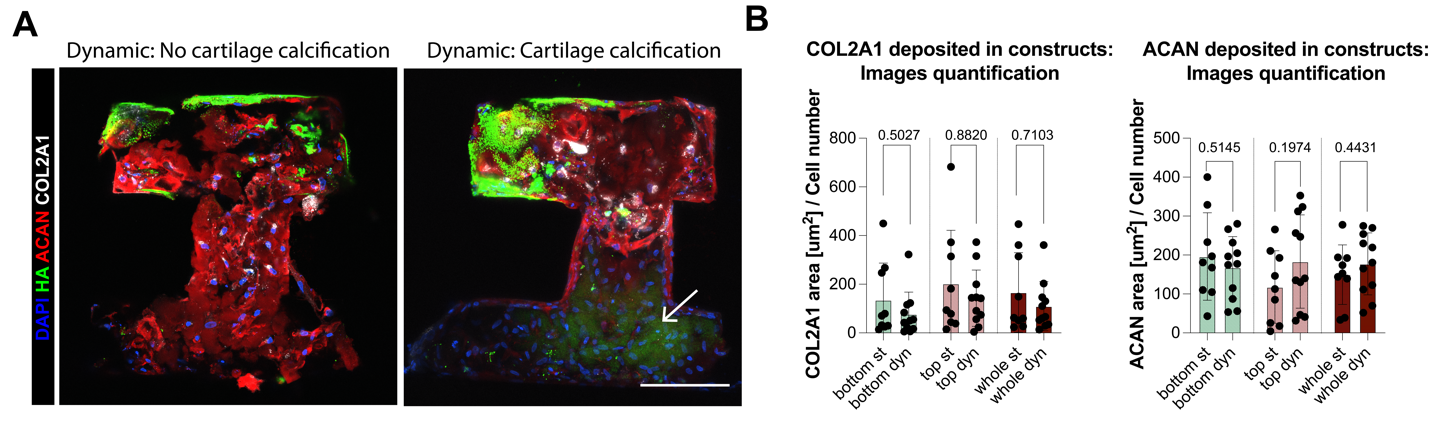


**Figure S15. Effect of compartment-specific HPC on OCU-on-Chip’s ECM deposition.** A) Representative immunofluorescence images of OCU-on-Chip sections after 14 days of static maturation and 7 days of cyclical mechanical loading (i.e. Day 21). The arrow points to an HA rich and ACAN/COL2A1 poor region in the cartilage compartment. Dynamically loaded constructs were characterized by the stochastic appearance of mineralized portions of the hyaline cartilage layer, which were negative for cartilage ECM markers. Scale bar, 100 µm. B) Quantifications of ACAN and COL2A1 stainings in OCU-on-Chip constructs performed using QuPath. Top areas were considered comprehensive of the VBV necking area. Statistical significance between static and cyclically loaded constructs was determined by two tailed unpaired t-test for normal populations and by Man-Whitney test for non-gaussian populations (N≥3 images per condition for each of n= 3 hACs and bmMSCs donors). Populations normality was assessed through Shapiro-Wilk and Kolmogorov-Smirnov tests. Values are reported as mean ± s.d.


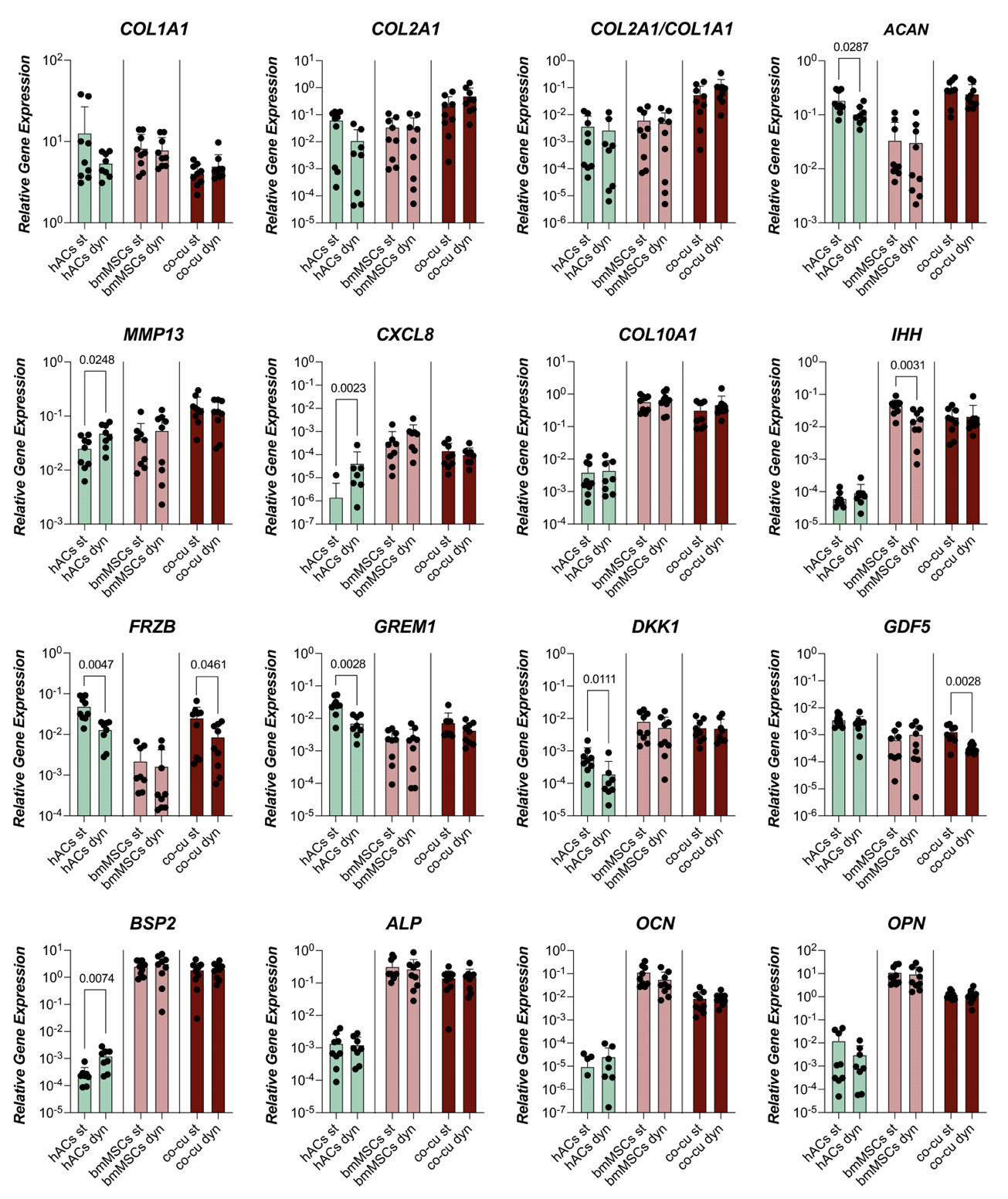
**Figure S16. Effect of HPC on hACs’, bmMSCs’, and OCU-on-Chip constructs’ gene expression.** RT-qPCR based quantification of the effect of cyclical HPC on hACs and bmMSCs single cultures and on whole OCU-on-Chip constructs (N=9 independently cultured samples from n=3 independent donors/experiments for each condition). Co-cu gene expression reefers to whole OCU-on-Chip constructs, comprehensive of both hACs and bmMSCs, and analyzed together. Statistical significance was determined by paired t-test for normal populations and Wilcoxon test for non-gaussian populations, respectively. (Adjusted) p-values ≤ 0.05 are reported on the graphs. Expression levels of all genes were normalized to *GAPDH* expression, values are reported as mean + s.d. Populations’ normality was assumed if both Shapiro-Wilk and Kolmogorov-Smirnov tests resulted positive.


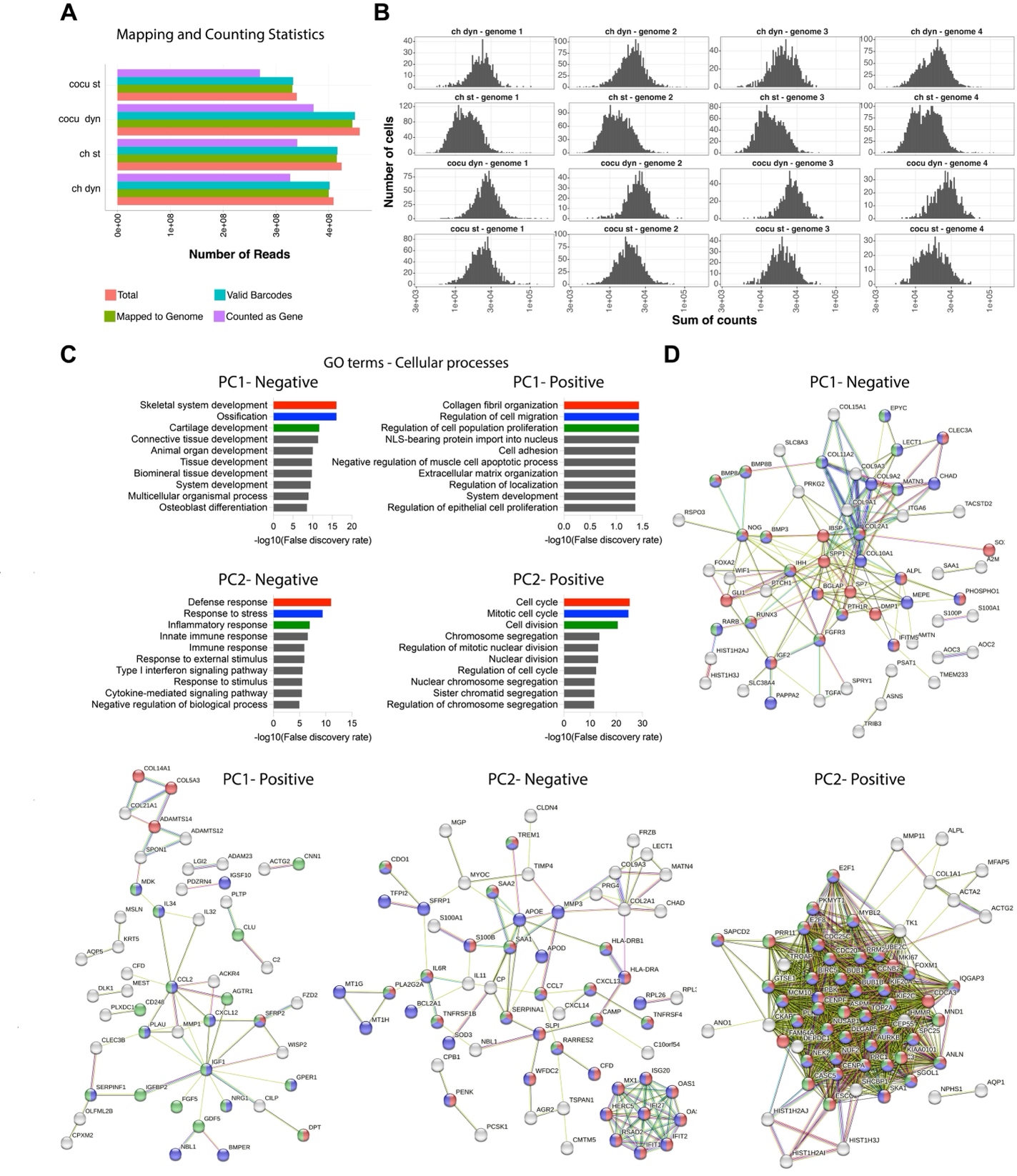


**Figure S17. Mapping statistics and Principal Component Analysis (PCA).** A) Mapping and counting statistics divided by samples’ culture conditions. B) Distribution of the sum of counts across cells used in downstream analyses divided by culture condition and genotype. C) Enriched GO terms (biological processes) obtained using the 100 most positively correlated and the 100 most negatively correlated genes along PC1 and PC2. GO enrichment analysis was performed using STRING. Graphs report the first 10 GO terms ordered according to the lowest False Discovery Rate. D) STRING-based protein-protein interaction network obtained considering the 100 most positively correlated and the 100 most negatively correlated genes along PC1 and PC2 respectively. Nodes are either grey or colored according to their relation to the GO terms with the lowest false discovery rate as reported in panel A. Non-connected network nodes are not reported.


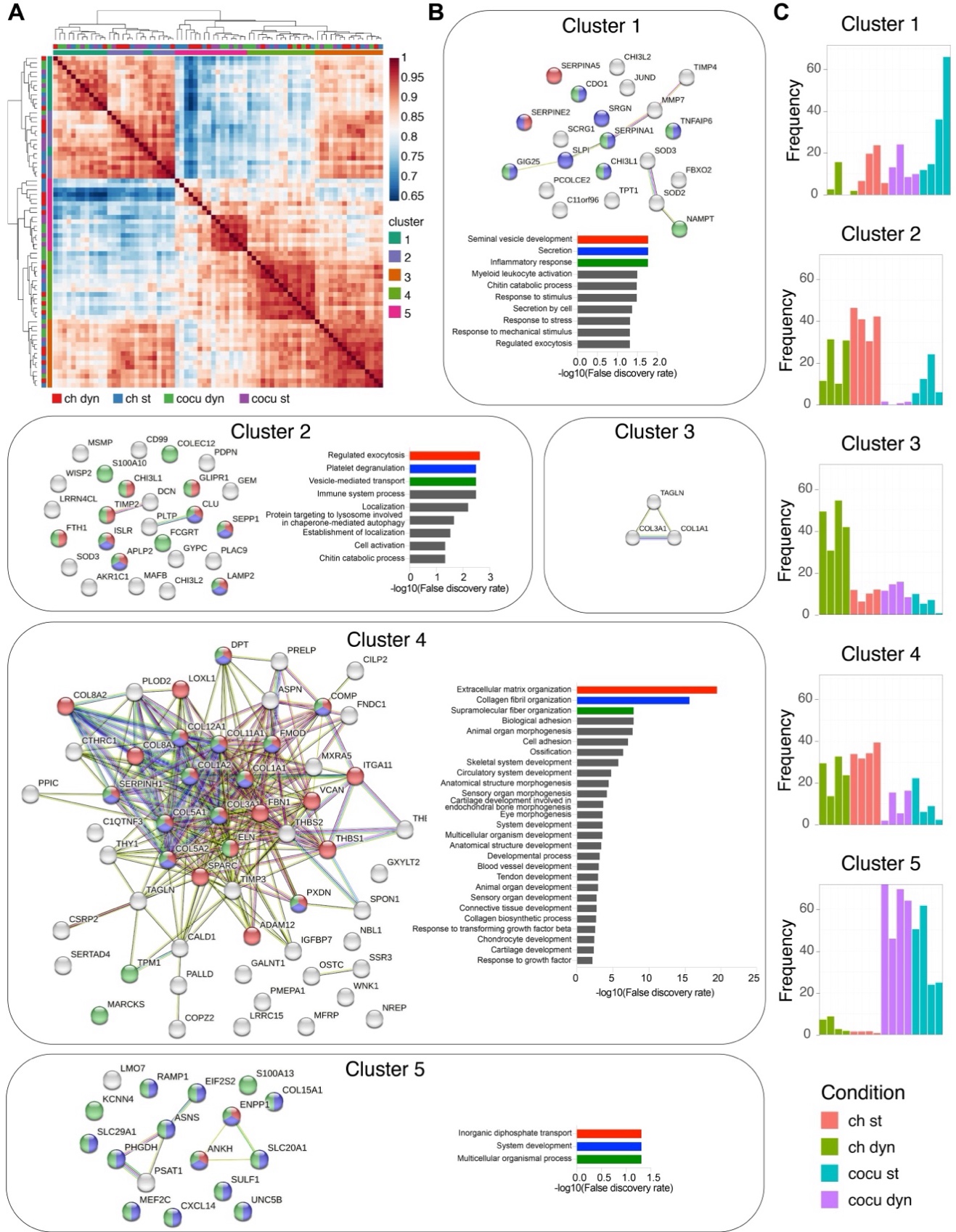


**Figure S18. Characterization of clusters’ marker genes.** **A)** Hierarchical clustering of gene expression correlation profiles. Assigned clusters and experimental conditions are indicated on the edges, following the color codes in the legend. **B)** Protein-protein interaction network and enriched GO terms (biological processes) for cluster marker genes, as identified using STRING. Network branches are colored based on interaction evidence. Nodes are either gray or color-coded according to their association with GO terms, as shown in the corresponding bar graphs. No statistically significant enriched GO terms for biological processes were found for cluster 3.
**C)** Relative frequency (%) of cells from different culture conditions in highlighted clusters. Each condition is represented by four bars, corresponding to the four donors used in the experiment.


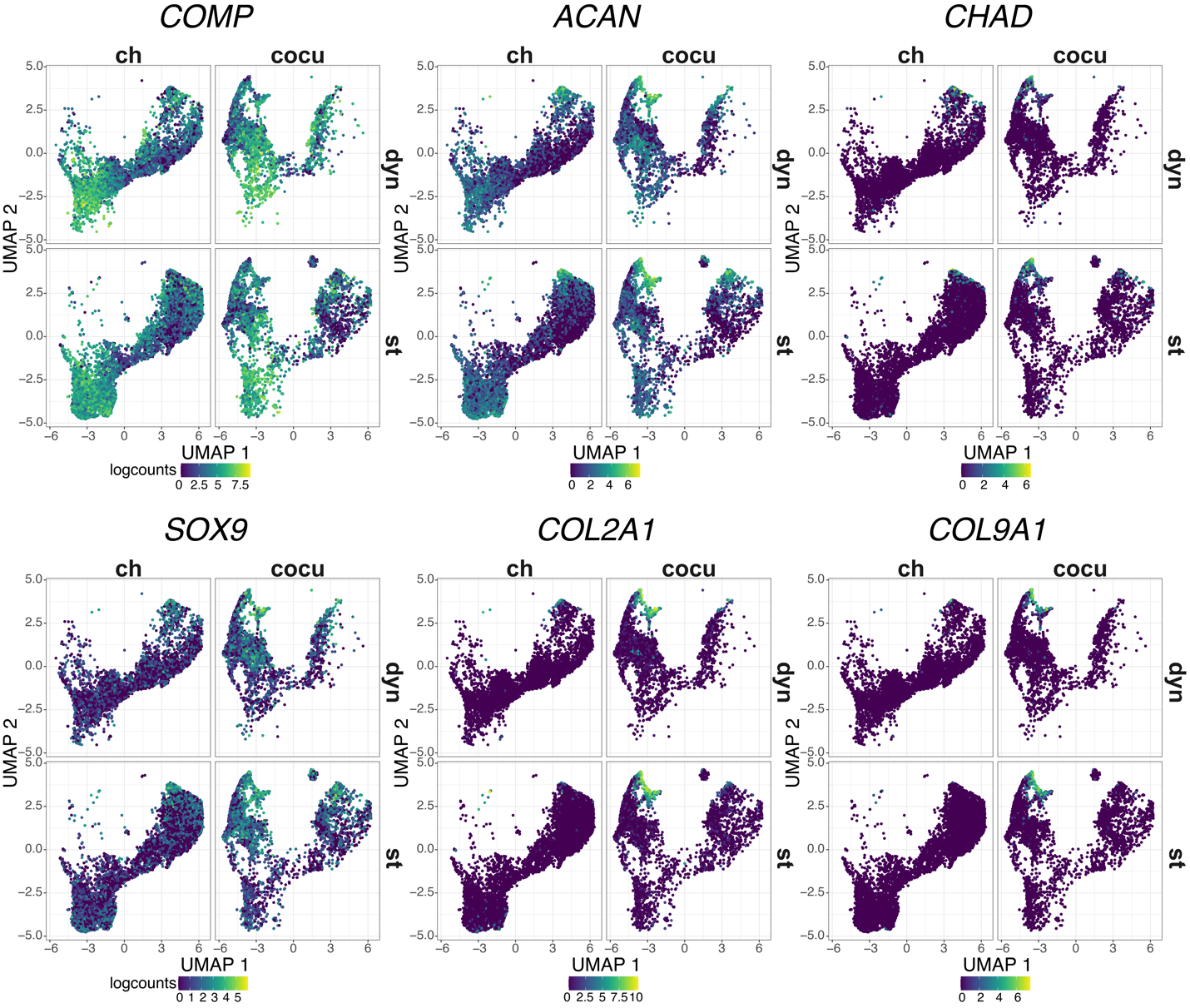


**Figure S19. Feature plots of classic chondrogenic markers.** Panels are divided by stimulation state (rows) and culture conditions (columns).


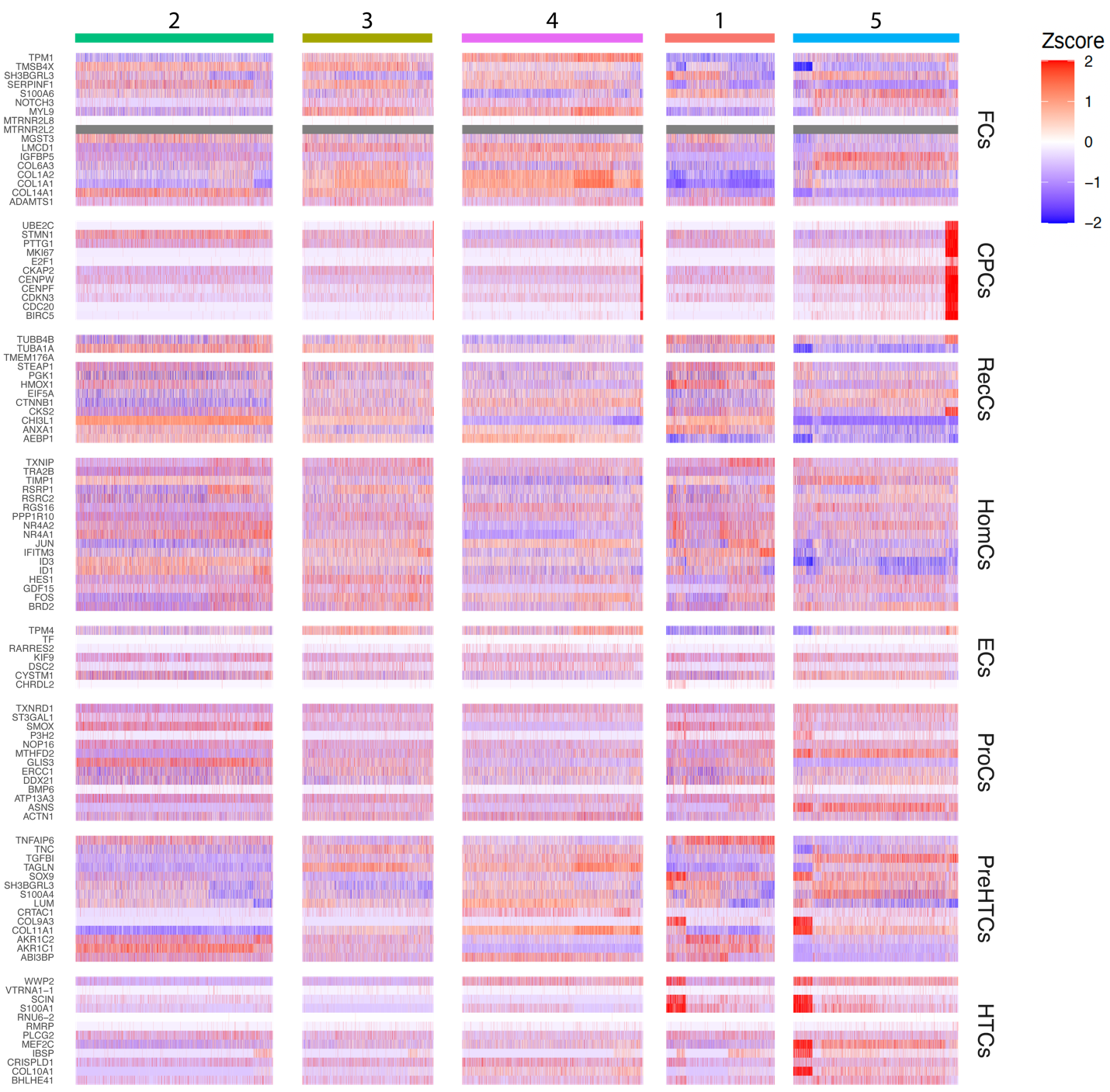


**Figure S20. Expression of marker genes of OA and healthy chondrocyte subpopulations across clusters.** Heatmap depicting the gene expression of chondrocytes subpopulations marker gens grouped by cluster. Each row represents a gene, and each column represents a cell. Marker genes were compiled from population marker genes defined by,^[13]^,^[25]^ and.^[14]^ For each subpopulation, the 16 most highly expressed marker genes from existing datasets were considered (when available). Subpopulation-rich clusters 1 and 5 are grouped together on the right.


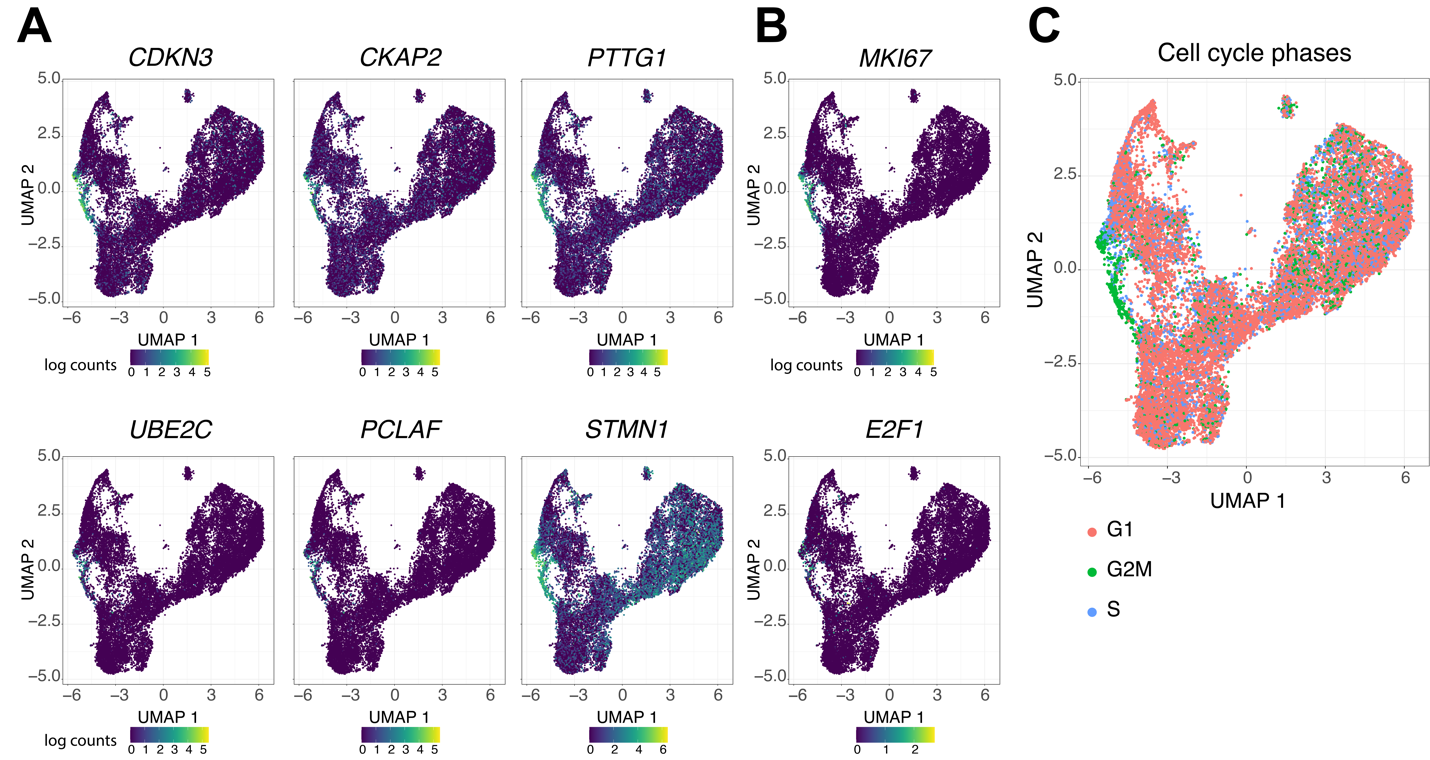


**Figure S21. Characterization of CPCs.** A) Feature plots of CPC markers as reported in.^[14]^ B) Feature plots of proliferation markers. C) UMAP embedding, cells are annotated according to the cell cycle phase.

**
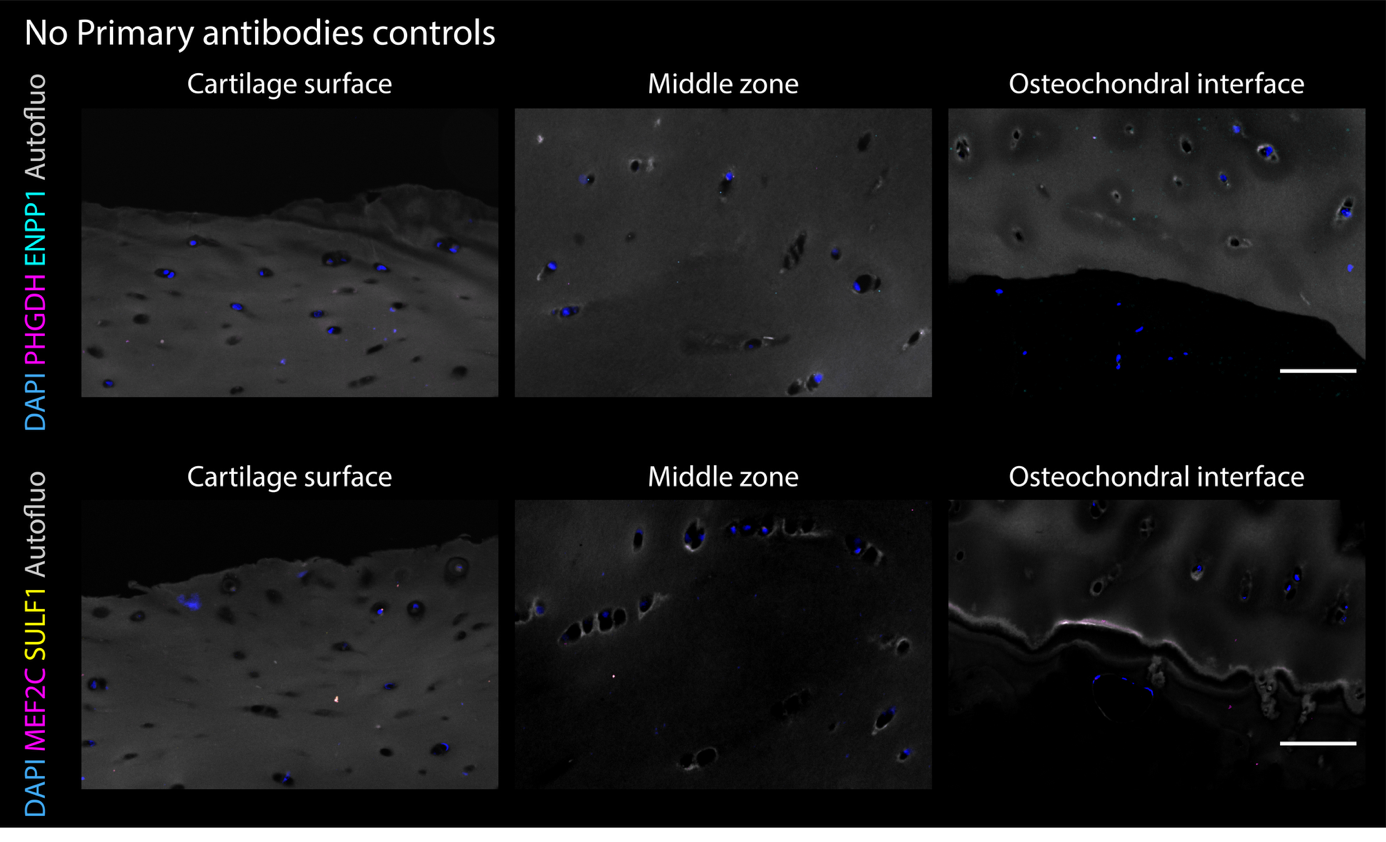
Figure S22. Cluster 5 markers stainings on human osteochondral samples: Controls without primary antibodies**. Immunofluorescence images of osteochondral biopsies taken from the distal femur of OA patients undergoing total knee arthroplasty performed without using primary antibodies. The images reveal the specificity of the stainings in Figure 7C. Scale bars, 100 µm.


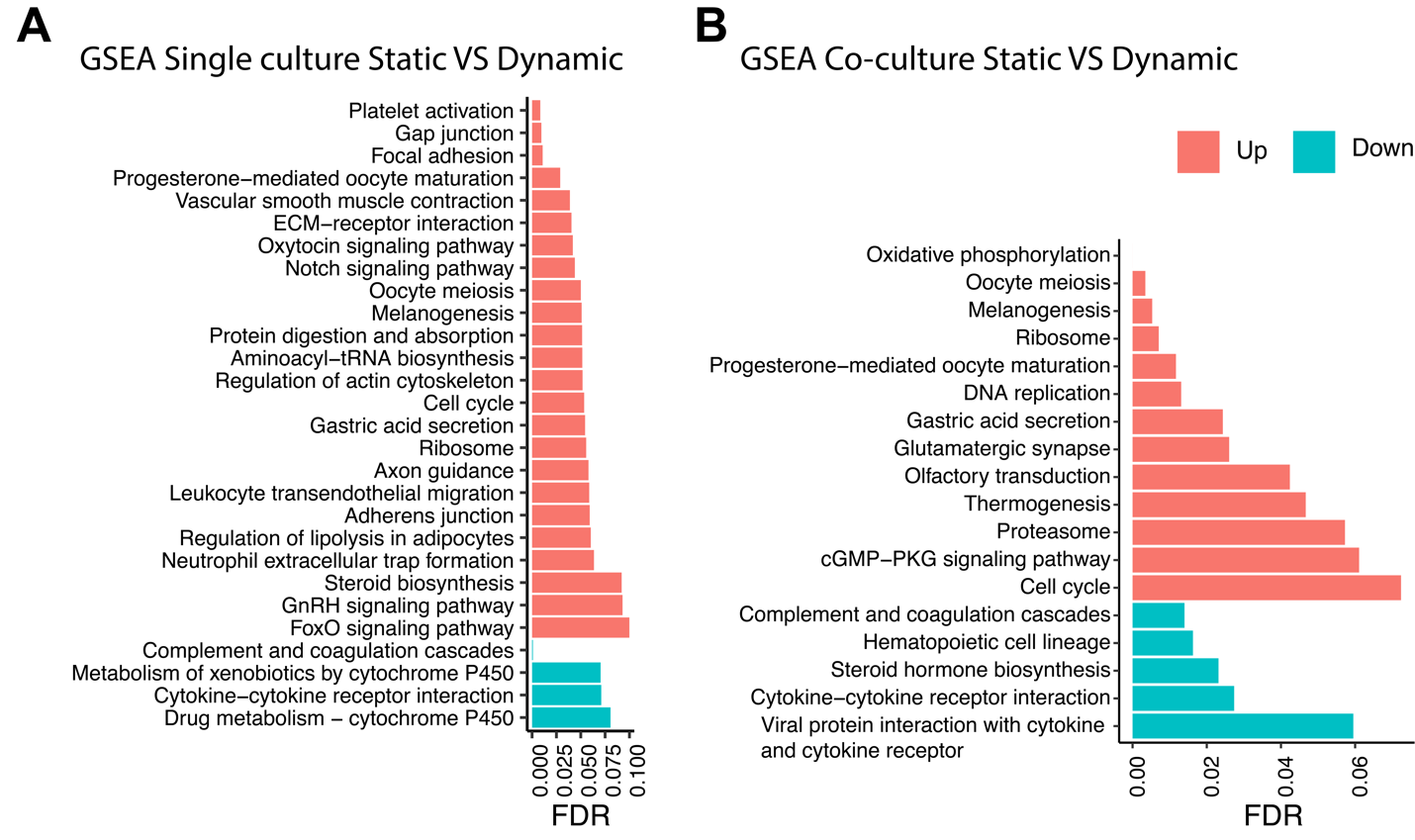


**Figure S23. KEGG pathways gene sets enrichment analyses from *in silico* bulk samples.** A) Single culture. B) Co-culture.


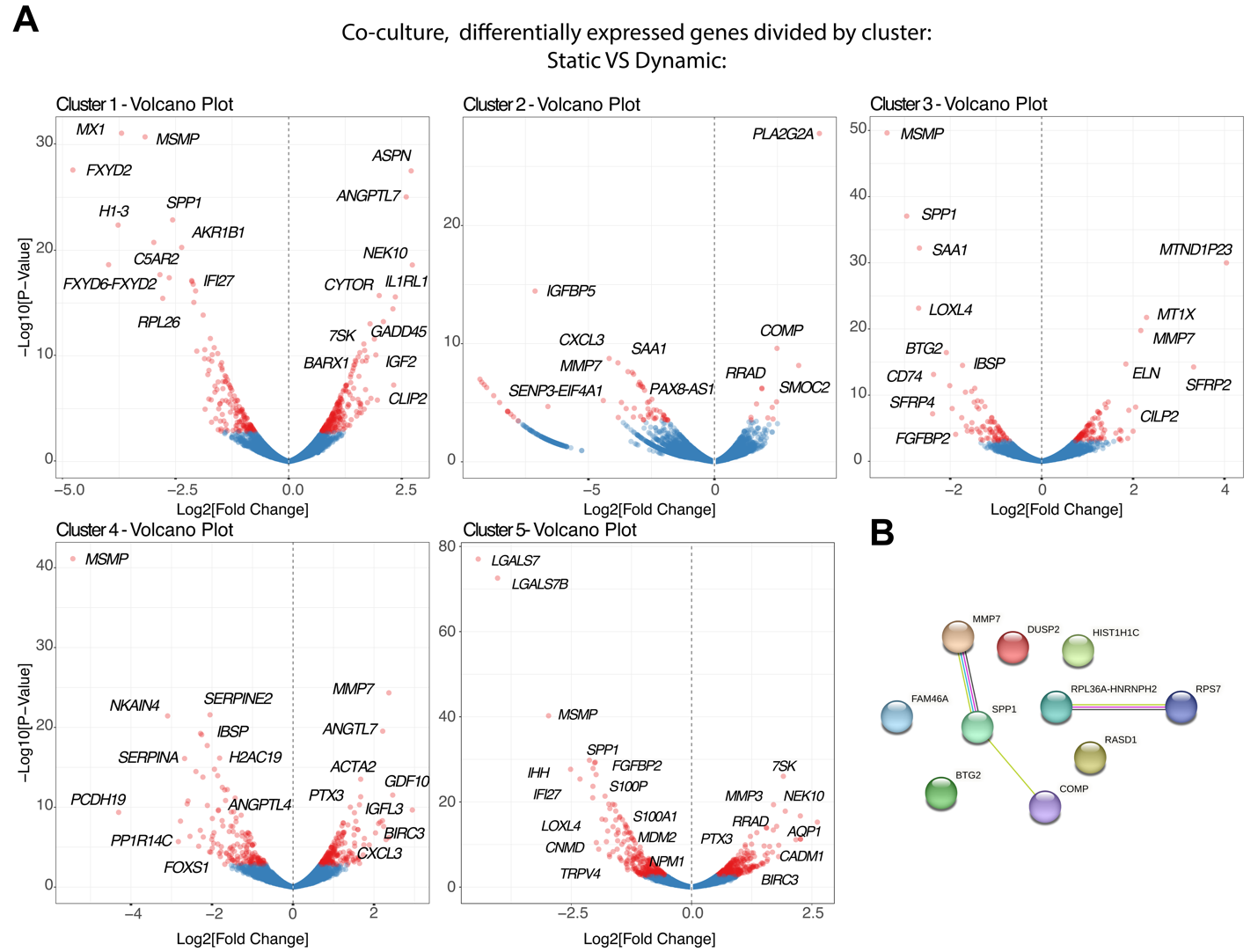


**Figure S24. Differentially expressed genes (static vs HPC dynamic samples) across clusters.** A) Volcano plot of DE genes obtained comparing static samples and samples subjected to HPC. DE expression refers to *in silico* bulk samples obtained summing the expression of all cells belonging to a given cluster and retrieved from OCU-on-Chip co-cultures. DEGs with and adj.p-value < 0.05 are indicated in red. Selected genes are labelled on the graphs. B) Protein-protein interaction network relative to DEGs that were common to all clusters. The network was obtained through STRING, including non-connected nodes in the representation. Branches in the network are colored according to the interaction evidence.

# **References**

[1] M. M. S. Nicholas D Evans, Caterina Minelli, Eileen Gentleman, Vanessa LaPointe, Sameer N Patankar, Maria Kallivretaki, Xinyong Chen, Clive J Roberts, *Eur Cell Mater* **2009**, *18*, 1.

[2] P. Occhetta, A. Mainardi, E. Votta, Q. Vallmajo-Martin, M. Ehrbar, I. Martin, A. Barbero, M. Rasponi, *Nat. Biomed. Eng.* **2019**, *3*, 545.

[3] M. R. DiSilvestro, J. K. F. Suh, *J. Biomech.* **2001**, *34*, 519.

[4] M. R. DiSilvestro, Q. Zhu, M. Wong, J. S. Jurvelin, J. K. F. Suh, *J. Biomech. Eng.* **2001**, *123*, 191.

[5] S. Hosmane, A. Fournier, R. Wright, L. Rajbhandari, R. Siddique, I. H. Yang, K. T. Ramesh, A. Venkatesan, N. Thakor, *Lab Chip* **2011**, *11*, 3888.

[6] D. E. A. Phelps, M. N. O. Enemchukwu, M. V. F. Fiore, D. J. C. Sy, P. N. Murthy, P. T. A. Sulchek, P. T. H. Barker, P. A. J. García, *Adv. Mater.* **2012**, *24*, 64.

[7] M. M. Blum, T. C. Ovaert, *J. Mech. Behav. Biomed. Mater.* **2012**, *14*, 248.

[8] X. Huang, Y. Huang, *Bioinformatics* **2021**, *37*, 4569.

[9] Y. Huang, D. J. McCarthy, O. Stegle, *Genome Biol.* **2019**, *20*, 1.

[10] C. Xu, Z. Su, *Bioinformatics* **2015**, *31*, 1974.

[11] V. D. Blondel, J. L. Guillaume, R. Lambiotte, E. Lefebvre, *J. Stat. Mech. Theory Exp.* **2008**, *2008*, P10008.

[12] A. T. L. Lun, K. Bach, J. C. Marioni, *Genome Biol.* **2016**, *17*, 1.

[13] Q. Ji, Y. Zheng, G. Zhang, Y. Hu, X. Fan, Y. Hou, L. Wen, L. Li, Y. Xu, Y. Wang, F. Tang, *Ann. Rheum. Dis.* **2019**, *78*, 100.

[14] X. Wang, Y. Ning, P. Zhang, B. Poulet, R. Huang, Y. Gong, M. Hu, C. Li, R. Zhou, M. J. Lammi, X. Guo, *Cell Death Dis.* **2021**, *12*, DOI 10.1038/s41419-021-03832-3.

[15] A. T. L. Lun, J. C. Marioni, *Biostatistics* **2017**, *18*, 451.

[16] B. Snel, G. Lehmann, P. Bork, M. A. Huynen, *Nucleic Acids Res.* **2000**, *28*, 3442.

[17] A. Subramanian, P. Tamayo, V. K. Mootha, S. Mukherjee, B. L. Ebert, M. A. Gillette, A. Paulovich, S. L. Pomeroy, T. R. Golub, E. S. Lander, J. P. Mesirov, *Proc. Natl. Acad. Sci. U. S. A.* **2005**, *102*, 15545.

[18] H. Cho, H. Y. Kim, J. Y. Kang, T. S. Kim, *J. Colloid Interface Sci.* **2007**, *306*, 379.

[19] K. S. Yun, E. Yoon, *Lab Chip* **2008**, *8*, 245.

[20] Y. Moser, R. Forti, S. Jiguet, T. Lehnert, M. A. M. Gijs, *Microfluid. Nanofluidics* **2011**, *10*, 219.

[21] P. KP, G. S, J. SA, O. K, P. JP, R. PA, S. D, van den B. WB, *Osteoarthr. Cartil.* **2006**, *14*, 13.

[22] Y. chun Chen, C. P. Brown, *J. Mech. Behav. Biomed. Mater.* **2020**, *104*, 103663.

[23] N. H. Varady, A. J. Grodzinsky, *Osteoarthr. Cartil.* **2016**, *24*, 27.

[24] L. Power, L. Acevedo, R. Yamashita, D. Rubin, I. Martin, A. Barbero, *Osteoarthr. Cartil.* **2021**, *29*, 433.

[25] Y. Qu, Y. Wang, S. Wang, X. Yu, Y. He, R. Lu, S. Chen, C. Meng, H. Xu, W. Pei, B. Ni, R. Zhang, X. Huang, H. You, *Comput. Biol. Med.* **2023**, *160*, 106926.
